# The relationship between chronic air pollution exposure, neighborhood environmental vulnerability, and adverse COVID-19 morbidities among hospitalized New York City residents

**DOI:** 10.1016/j.envint.2025.109660

**Published:** 2025-07-02

**Authors:** Sneha Kannoth, Cong Zhang, Mehr Shafiq, Sandra S. Albrecht, Alexander Azan, Earle C. Chambers, Min Qian, Perry E. Sheffield, Azure Thompson, Jennifer A. Woo Baidal, Stephanie Lovinsky-Desir, Jeanette A. Stingone

**Affiliations:** a Columbia University, Mailman School of Public Health, New York, NY, USA; b New York University Langone Health, Department of Population Health, New York, NY, USA; c Albert Einstein College of Medicine, Department of Family and Social Medicine, Bronx, NY, USA; d Icahn School of Medicine at Mount Sinai, Department of Environmental Medicine, New York, NY, USA; e SUNY Downstate Health Sciences University, School of Public Health Brooklyn, NY, USA; f Stanford University, Stanford School of Medicine Stanford, CA, USA; g Columbia University, Vagelos College of Physicians and Surgeons, New York, NY, USA

**Keywords:** Long-term air pollution, COVID-19 morbidities, Environmental vulnerability, Social vulnerability, Health disparities

## Abstract

**Introduction:**

Communities disproportionately burdened by adverse neighborhood-level social and structural factors may experience greater vulnerability to environmental exposures, contributing to health inequities, including adverse COVID-19. We assessed the effects of chronic air pollution on COVID-19 morbidities in NYC and examined whether these effects varied by neighborhood-level vulnerability.

**Methods:**

We used NYC COVID-19 hospitalization records (3/1/2020–2/28/2021) and conducted analyses in the full sample and within hospital catchment. Chronic air pollution (particulate matter (PM_2.5_), nitrogen dioxide (NO_2_), black carbon (BC), ozone (O_3_)) was assigned using residential ZIP Code (NYC Community Air Survey; 2009–2019). Modified Poisson regression estimated risk of acute respiratory distress syndrome (ARDS), pneumonia, ventilation, and dialysis, and Cox regression estimated risk of discharge, adjusting for age, sex, BMI, smoking, asthma, diabetes, and hypertension. We assessed effect modification by neighborhood-level environmental vulnerability index (NEVI) tertiles.

**Results:**

From March to June 2020 (within hospital catchment), adjusted estimates generally suggest greater chronic NO_2_, PM_2.5_, and BC was associated with increased risk of ARDS, pneumonia, and dialysis, and not associated with discharge and ventilation; inverse estimates found for chronic O_3_. Relationships between air pollution and adverse COVID-19 were generally stronger among those with greater neighborhood environmental vulnerability. For example, chronic NO_2_ and pneumonia’s relationship was stronger in individuals within higher NEVI tertiles (T1: aRR: 1.13, 95%CI: 1.02–1.25; T2: aRR: 2.11, 95%CI: 1.73–2.56; T3: aRR: 6.36, 95%CI: 4.71–8.60).

**Discussion:**

Differences in neighborhood-level social and structural factors contribute to unequal health burdens associated with air pollution. Public health resources targeted toward neighborhoods with greater environmental vulnerability can encourage population-level pandemic preparedness.

## 1. Introduction

The COVID-19 pandemic highlighted existing population-level health inequities on a US and global scale (Nana-Sinkam et al., 2021; Adhikari et al., 2020; Bassett et al., 2020; Tan et al., 2020; Zhang and Schwartz, 2020), warranting further focus in examining how population-level exposures, such as chronic air pollution exposure, may influence adverse COVID-19 morbidities. Chronic ambient air pollution exposure is defined as long-term air pollution exposure (e.g., measured across years to decades) in outdoor environments (Sidell et al., 2022; Landrigan, 2017), and may stem from multiple sources, including traffic-related pollution and industrial emissions (Landrigan, 2017). Current ecological and cohort studies suggest that greater air pollution exposure is associated with greater burden of severe COVID-19 morbidities measured on a group (Cole et al., 2020; Liang et al., 2020; Marques and Domingo, 2022; Wu et al., 2020; Sahu et al., 2021; Paez-Osuna et al., 2022; Semczuk-Kaczmarek et al., 2022; Yao et al., 2020; Zang et al., 2022; Perone, 2022; Filippini et al., 2021; Hutter et al., 2020; Mathys et al., 2023; Liu and Li, 2020) and individual level (Zhang et al., 2023; Bozack et al., 2022; Lopez-Feldman et al., 2021; Bowe et al., 2021a,b; Chen et al., 2022; Mendy et al., 2021; Beloconi and Vounatsou, 2023). In better understanding the relationship between chronic ambient air pollution exposure and adverse COVID-19 morbidities, it is integral to consider a population’s level of vulnerability to environmental exposures. A population’s level of vulnerability can be defined by neighborhood-level social and structural factors, such as population-level health behaviors (Bowe et al., 2021), median population-level household income (Chen et al., 2022), and population density (Bowe et al., 2021).

It is important to explore the role of neighborhood-level social and structural factors (Vlahov et al., 2007) in influencing the vulnerability to environmental exposures on health. Firstly, defining the role of neighborhood-level social and structural factors in the relationship between air pollution and adverse COVID-19 has important ramifications for understanding the observed health disparities in COVID outcomes. Populations, where the majority of individuals are racial or ethnic minorities or are low-income, may experience greater vulnerability to environmental exposures, by experiencing adverse social and structural factors (Vlahov et al., 2007; Smith et al., 2022); thereby, inducing greater risk of adverse COVID-19 outcomes. Populations’ differential vulnerability to environmental exposures stems from systemic racism, which contribute to a stark disinvestment in housing and paths to class mobility (Hwa Jung et al., 2022). This disinvestment may have led communities to experience greater vulnerability to environmental exposures, via low housing quality, and greater proximity to pollution sources, such as vehicular traffic, chemical factories, and electric plants (Lane et al., 2022; Cushing et al., 2023). Secondly, understanding the role of neighborhood-level social and structural factors in influencing environmental vulnerability can help inform public health interventions and resource allocation, to reduce the burden of air pollution’s effect on health among target populations.

Some studies have examined the contribution of social and structural factors in explaining the relationship between air pollution and adverse COVID-19 morbidities. For example, studies have examined how individual factors such as population density (Lopez-Feldman et al., 2021; Chen et al., 2022; Beloconi and Vounatsou, 2023; Zhang et al., 2023), average household size (Beloconi and Vounatsou, 2023), income per resident (Chen et al., 2022; Beloconi and Vounatsou, 2023; Zhang et al., 2023), number of hospital beds (Beloconi and Vounatsou, 2023), and number of healthcare workers in the health sector (Beloconi and Vounatsou, 2023), may influence the relationship between air pollution and adverse COVID-19 morbidities. The first limitation of existing literature is that existing studies have not fully explored how neighborhood-level social and structural factors influence the relationship between air pollution and adverse COVID-19 (Bowe et al., 2021; Chen et al., 2022; Beloconi and Vounatsou, 2023; Zhang et al., 2023; Huang et al., 2021). In contrast, we hypothesize that neighborhood-level social and structural factors may modify the relationship between air pollution and adverse COVID-19. We propose that neighborhood-level social and structural factors may predispose individuals to experience greater vulnerability to the effects of air pollution exposure on adverse COVID-19. Thus, we aimed to compare the relationship between air pollution and adverse COVID-19 morbidity across different levels of neighborhood environmental vulnerability. We hypothesize that the relationship between air pollution and adverse COVID-19 may be stronger among those with adverse neighborhood-level social and structural factors that lead to greater experience of environmental vulnerability. The second limitation is that previous literature that looked at the relationship between air pollution and adverse COVID-19 morbidities often individually adjusted for each social and structural factor. Given that populations are often simultaneously exposed to a range of co-occurring, adverse factors in the context of disinvested neighborhoods and communities, we also hypothesize that it is more reasonable to look at the combination of social and structural factors that operationalize neighborhood-level vulnerability to environmentally-related health outcomes, using an index measure (Ferreira et al., 2021; Rogers and Lange, 2013). In aiming to explain the relationship between air pollution and adverse COVID-19, there is a need to better operationalize and define the neighborhood-level vulnerability to environmental exposures.

Overall, our study aim is to estimate the relationship between chronic air pollution exposure and an individual’s risk of adverse COVID-19 morbidity in NYC and to determine if these effects vary by neighborhood-level environmental vulnerability, as defined by an index of neighborhood-level demographic, economic, residential and health features. We constructed a retrospective cohort study of COVID-19 related hospital admissions in 2020 and 2021 using a harmonized repository of electronic health record (EHR) data from multiple healthcare institutions in New York City (NYC). NYC is the ideal setting for the investigation of the relationship between chronic air pollution exposure, adverse COVID-19 morbidities, and neighborhood environmental vulnerability due to the large number of COVID-19 cases, variability in chronic air pollution exposures, and the existence of structural drivers of environmental vulnerability that are related to the diverse racial, ethnic, and socioeconomic make-up of NYC neighborhoods. Accordingly, we hypothesize that greater chronic air pollution exposure would be associated with adverse COVID-19 morbidities and the relationship between chronic air pollution exposure and adverse COVID-19 outcomes would be stronger among NYC neighborhoods with greater vulnerability to environmental exposures, as defined by an index of neighborhood-level demographic, economic, residential, and health features.

## 2. Methods

The Columbia University Institutional Review Board approved all study procedures (IRB AAAT2933).

### 2.1. Study population

We used harmonized EHR data from metropolitan healthcare systems in NYC, compiled within the INSIGHT clinical research network (INSIGHT-CRN) (Kaushal et al., 2014). One of the largest clinical data networks in the United States, INSIGHT-CRN receives data from the following large healthcare systems: Albert Einstein School of Medicine/Montefiore Medical Center, Columbia University and Weill Cornell Medicine/New York-Presbyterian Hospital, Icahn School of Medicine/Mount Sinai Health System and New York University Grossman School of Medicine/Langone Medical Center. Each institution agreed to harmonize their clinical records to a common ontology and then deposit their data into a centralized repository that can be accessed by the scientific community for research purposes. This enabled the linkage of individuals’ data across time and across institutions in NYC. The INSIGHT-CRN created a COVID-19 extract that included all individuals with a COVID-19 test or diagnosis. In addition, 10-year historical INSIGHT-CRN EHR records (i.e., INSIGHT data collection began in 2011) were included in the data extract.

Our study population was restricted to all hospital admission records for individuals with a COVID-19 diagnostic code from March 1, 2020, through February 28, 2021, who reported a NYC ZIP Code of residence. The study population was stratified based on date of admission into three categories that correspond to phases of the pandemic in NYC: the first wave: March 2020-June 2020 (Phase 1), the reduced transmission period after the first wave: July 2020-October 2020 (Phase 2), and the beginning of the second wave before the widespread introduction of COVID-19 vaccines: November 2020-February 2021 (Phase 3). Adjacent admissions (i.e., when the discharge date of the 1st hospitalization encounter was the same date as the admission date of the 2nd hospitalization encounter) were merged together into a single encounter. Repeat admissions with at least one full day between discharge and admission were retained as repeat, separate admissions.

### 2.2. Outcomes and hospital-based data

We examined hospital-based outcomes and procedures that generally correspond to more severe morbidity during admission for COVID-19 (Attaway et al., 2021; Tzotzos et al., 2020; Grasselli et al., 2021). These morbidities include longer length of stay, acute respiratory distress syndrome (ARDS), pneumonia, use of mechanical ventilation, and use of dialysis. ARDS is a severe sequela of COVID-19 that is characterized by hypoxemic respiratory failure due to inflammatory injury in the lung. This damage results in the accumulation of fluid in lung airspaces, reducing the delivery of oxygen to vital organs (Batah and Fabro, 2021). Pneumonia characterizes an infection in the lungs. Mechanical ventilation procedures include the placement of an endotracheal tube and life-sustaining respiratory support for individuals who have respiratory failure (Mohlenkamp and Thiele, 2020). Dialysis procedures are implemented in response to kidney failure due to COVID-19, and help supplement the kidney’s role in helping clean toxins, and removing excess water and waste from the blood (Durvasula et al., 2020). Individuals with a record of dialysis treatment prior to their COVID-related admission were removed from the dialysis analyses.

We identified if a patient experienced adverse COVID-19 morbidities by using ICD10 and Current Procedural Terminology (CPT) codes. The corresponding ICD10/CPT codes for adverse COVID-19 morbidities are as follows: ARDS (ICD10 Codes: J80, B97), viral pneumonia (ICD10 Codes: J12.8, J12.81, J12.82, J12.9) (DuBose et al., 2023; Rao et al., 2023), mechanical ventilation procedures (ICD10-PCS (procedure coding system) codes: 5A1935Z, 5A1945Z, 5A1955Z; CPT codes: 94002), and dialysis procedures (ICD10 codes: R88.0, Z49.01, Z49.02, Z49.31, Z49.32; CPT Codes: 90935, 90937, 90939, 90940, 90941, 90942, 90943, 90944, 90945, 90996, 90997, 90998, 90999). Individual-level patient length of stay was calculated as the difference between admission and discharge hospitalization dates (AAPC, 2025).

Individual characteristics, including age, sex, race/ethnicity, body mass index (BMI), and smoking status were noted in everyone’s admission record at the time of COVID-19 admission. When BMI or smoking status were missing from the COVID-19 admission record, data from prior hospitalizations were used, when available. As noted above, INSIGHT contains hospital-based records data back to 2011. Similarly, individuals with chronic diseases noted anywhere within INSIGHT prior to the COVID-19 related admission were recorded as having those conditions. Chronic disease variables were created for diabetes, asthma and hypertension using ICD10 Codes.

### 2.3. Exposure assessment: chronic air pollution at residential ZIP code

NYCCAS was developed by the NYC Department of Health and Mental Hygiene (DOHMH) and Queens College of City University of New York (Clougherty et al., 2013). Since December 2008, NYCCAS has been measuring multiple pollutants, temperature, and humidity using data loggers, at 155 systematically-distributed sites throughout NYC. Monitors are mounted at 10 to 12 feet on public utility poles, and samples are collected for two-week periods, once per season. These sampling data are combined with land-use variables within a land-use regression model to construct annual averages of pollutants, represented geospatially at a 300-meter resolution.

For this study, we aggregated ambient concentrations to the ZIP Code level, as the patients’ residential ZIP Code was the smallest geographic indicator available for the clinical data. For each ZIP Code, we calculated 11-year averages (2009–2019) for the following four pollutants: fine particulate matter (PM_2.5_), black carbon (BC), nitrogen dioxide (NO_2_) and ozone (O_3_). Note that due to drastic seasonal fluctuations, O_3_ estimates are modeled as a seasonal average during summer months as opposed to an annual average (Clougherty et al., 2013). We examined these four ambient air pollutants as NYCCAS collected sampling data pertaining to PM_2.5_, BC, NO_2_, and O_3_ from 2009 to 2019. Furthermore, existing literature suggests that these key ambient air pollutants are strongly associated with adverse COVID-19 morbidities and adverse respiratory health outcomes, providing the basis for the focus on these pollutants for this study (Zhang et al., 2023; Bozack et al., 2022; Lopez-Feldman et al., 2021; Bowe et al., 2021a,b; Chen et al., 2022; Mendy et al., 2021; Beloconi and Vounatsou, 2023).

Pollutant variables were scaled to correspond to an interquartile range (IQR) contrast and categorical variables using quartiles were constructed to assess linearity. Each individual was assigned exposure based on the residential ZIP Code within the hospital admission record. We also created 5-year pollution averages (2015–2019), and the prior 1-year average (2019) for each ZIP Code but did not see differences in the rank of chronic air pollution exposure metrics at the ZIP Code level with shorter time averages.

We defined our exposure as chronic air pollution exposure from 2009 to 2019 as we were interested in assessing long-term air pollution exposure prior to the pandemic period, and we did not include 2020, given the dramatic reduction in air pollution in 2020 due to the pandemic. If we had included the pandemic period, the estimation of chronic air pollution exposure, especially for areas in NYC with more stringent lockdowns, would be greatly reduced. As a result, we would not have accurately captured the typical levels of chronic air pollution exposure levels in NYC by ZIP Code; thereby, biasing the associations between chronic air pollution exposure and adverse COVID-19 outcomes.

### 2.4. Neighborhood environmental vulnerability measure

A primary aim of this study was to determine if neighborhood-level environmental vulnerability modifies the relationship between chronic air pollution exposure and adverse COVID-19 morbidities. Therefore, we constructed the neighborhood environmental vulnerability index (NEVI) for each residential ZIP Code in NYC using a profiling and clustering approach known as the toxicological prioritization index (ToxPi) (Reif et al., 2010). Using ToxPi, data from the 2015–2019 US Census American Community Survey, and the 2020 CDC PLACES Project, we quantified factors associated with neighborhood-level environmental vulnerability for NYC (Uong et al., 2023). More specifically, we quantified overall vulnerability and in 4 primary domains (demographic, economic, residential, and health status), 24 subdomains, and 54 distinct area-level features for NYC (see Supplement Table 1). The overall NEVI score represents a composite index of demographic, economic, residential, and health characteristics at the neighborhood-level. In characterizing the domain-specific NEVI scores, the demographic domain-specific score includes variables such as the percent of the neighborhood population that is 65 years and older, the economic domain-specific score includes variables such as the percent of the neighborhood that is unemployed, the residential domain-specific score includes variables such as the population density of a neighborhood, and the health domain-specific score includes variables such as the percent of neighborhood without health insurance. Domain-specific scores allow us to characterize the specific source of a neighborhood’s vulnerability to environmental exposures, such as whether the neighborhood environmental vulnerability may primarily stem from economic sources, as opposed to demographic, residential, and health sources.

In choosing the domains, subdomains, and distinct area-level features, we conducted a literature search on social and structural drivers of vulnerability to environmental pollution. We additionally examined published vulnerability indices, such as HGBEnviroScreentool (Bhandari et al., 2020) and the NIEHS COVID Index (Marvel et al., 2021), and used our research team’s subject matter knowledge to identify the final set of features to be included in the NEVI. Regarding scoring, overall and domain-specific indices were calculated by summing standardized feature values within the subdomains and then aggregating and weighting based on the number of features within each subdomain for each equally-weighted primary domain. Scores were originally calculated at the census-tract level and then aggregated to the ZIP Code level using population-based weights. Each individual was assigned overall NEVI and domain-specific scores based on their residential ZIP Code within the hospital admission record. Scores ranged from 0 to 1, with a higher value indicating greater neighborhood vulnerability. We have demonstrated that the NEVI is correlated with other area-level vulnerability measures, such as the Neighborhood Deprivation Index and the Social Vulnerability Index (Stephen et al., 2023). Additionally, NEVI is associated with area-level measures of environmentally related diseases, such as pediatric asthma exacerbation, across metropolitan cities in the US (Kannoth et al., 2023).

### 2.5. Main analyses

#### 2.5.1. Analyses within harmonized EHR data

Cox proportional hazards models were constructed to examine associations between chronic air pollution exposure and length of stay within the INSIGHT data. In the length of stay analyses, the outcome was discharge and deaths were censored. Note that we found some violations of Cox proportional hazards assumption across the 2020–2021 period (specifically, p < 0.05 for PM_2.5_, NO_2_, and O_3_), given that the effect of chronic ambient air pollution exposure on risk of discharge is not constant in this time period. We do not expect a constant effect of chronic ambient air pollution exposure on risk of discharge during this time period, given that the lockdown in the spring of 2020 likely coincided with reduced ambient air pollution emissions, and the late 2020 and early 2021 time period was marked by changing healthcare practices that may have influenced patient risk of discharge. Therefore, in an effort to account for the violations of the proportional hazards assumption, we stratified the analyses by phase. We additionally chose to construct Cox proportional hazards regression models for the length of stay analyses, given that Cox proportional hazards regression is semi-parametric, and does not assume a specific survival distribution; whereas, accelerated failure time models are parametric models that require knowledge of the survival distribution.

Modified Poisson regression models with robust standard errors were used to estimate the risk ratios corresponding to chronic air pollution exposure and binary measures of adverse COVID-19 morbidity, including ARDS diagnosis, pneumonia diagnosis, use of mechanical ventilation, and use of dialysis. (Bozack et al., 2022) We additionally provided overdispersion diagnostics for the Poisson regression models. For all models, chronic air pollution exposure was represented as a continuous linear term unless categorical analysis revealed evidence of departures from linearity. Analyses were performed within phases of the pandemic. We combined Phases 2 and 3, due to the low sample size of admissions during Phase 2. Furthermore, Phases 2 and 3 are more operationally similar due to the clinical lessons learned during the first wave of the COVID-19 pandemic.

For all presented results, pollutants were modeled as continuous linear terms as categorical analyses did not reveal considerable evidence of non-linearity (Supplement Tables 2a–d). Pollutant contrasts were constructed to approximately align with the interquartile range. O_3_ and PM_2.5_ were modeled as 1-unit differences in parts per billion (ppb) and micrograms per cubic meter respectively. BC was scaled to be a 2-unit difference in absorbance units while NO_2_ was scaled to represent a 5-ppb change. In addition, all presented results used the overall NEVI index. Models were also constructed with each domain individually (Supplement Tables 3a–d), and results were generally consistent across all domains except residential. Supplement Table 4 provides the dispersion statistics for the Poisson regression models examining the relationship between chronic ambient air pollution exposure and adverse COVID-19 morbidities, including ARDS, pneumonia, ventilation use, and dialysis use. The dispersion statistic is estimated as the ratio of the residual deviance to the corresponding degrees of freedom, and a dispersion statistic greater than 1 suggests overdispersion. All Poisson regression models yielded a dispersion statistic less than 1, suggesting no evidence of overdispersion.

#### 2.5.2. Confounding

Relevant confounders were identified through directed acyclic graph analysis (Shrier and Platt, 2008). Using Dagitty (Textor et al., 2016), we obtained our minimally sufficient adjustment set that included age, chronic disease (asthma, diabetes, hypertension), sex, smoking and BMI in order to obtain an estimate of the association.

#### 2.5.3. Effect measure modification

Effect measure modification between single air pollutants and NEVI was formally assessed on the multiplicative scale (using interaction terms at an alpha-level of 0.05). We provided estimates corresponding to the models adjusted for neighborhood environmental vulnerability, and the corresponding p-value for evidence of multiplicative interaction. We additionally stratified by tertile of the NEVI distribution within NYC and reported stratum-specific estimates of the relationship between chronic air pollution exposure and adverse COVID-19 morbidities. We constructed NEVI tertiles using the full NEVI distribution in NYC. In stratifying by NEVI tertiles, we help increase the interpretability of the results, instead of reporting the interaction coefficients between two continuous features. All study analyses were performed using the R statistical software (v4.1.2) (R Core Team, 2021).

### 2.6. Sensitivity analyses

We performed several sensitivity analyses to better examine our study findings.

#### 2.6.1. Two-pollutant analyses

Given the strong correlation between the pollutants which may suggest similar underlying processes, we did not conduct a mixture analysis across all pollutants (e.g., PM_2.5_, NO_2_, BC). As O_3_ was inversely correlated with the other pollutants given that O_3_ is a secondary pollutant to NO_2_, we conducted two-pollutant analyses where we simultaneously adjusted for O_3_ to account for potential competing effects of O_3_. All models were adjusted for age, sex, BMI, smoking status, asthma, diabetes, hypertension, and NEVI.

#### 2.6.2. Racial/ethnic subgroup analyses

We assessed the persistence of racial/ethnic disparities by conducting analyses within racial and ethnic subgroups. We examined the relationship between chronic air pollution exposure and adverse COVID-19 morbidities, after adjusting for age, sex, BMI, smoking status, asthma, diabetes, hypertension, and NEVI, within racial subgroups (Black; White); and ethnic subgroups (Hispanic; Non-Hispanic). If there was statistically significant evidence of interaction between the racial and/or ethnic subgroups (p < 0.05), then we presented the stratified estimates by racial subgroup and/or ethnic subgroup. We provided the p-value for interaction between subgroup level and pollutant exposure for adverse COVID-19 risk. If the hypothesized effect modification by neighborhood-level social and structural factors fully explains racial disparities in associations between air pollution and adverse COVID-19 outcomes, we would expect no stratum-specific differences by race and ethnicity in these analyses. Analyses were conducted from March to June 2020, within areas of greater hospital catchment.

#### 2.6.3. Additive interaction analyses

In addition to examining multiplicative interaction, we examined additive interaction between chronic air pollution exposure and neighborhood environmental vulnerability in influencing the risk of adverse COVID-19 morbidities. We operationalized NEVI as a dichotomous variable (High NEVI: upper tertile (score 3.49–4.99); Low NEVI: lower two tertiles (score 2.14–3.48)), for ease of interpretability. We operationalized chronic air pollution exposure continuously, using an interquartile range (IQR) increase. Studying an increase in IQR in chronic air pollution exposure on the risk of adverse COVID-19 morbidity allows for comparability with other studies that examines the relationship between chronic air pollution exposure and adverse COVID-19, considering that different study populations have different ranges of chronic air pollution exposure. Furthermore, in examining the relationship between chronic air pollution exposure when operationalized categorically using quartiles, and adverse COVID-19 morbidity, we did not see departures of additivity, allowing us to use chronic air pollution as a continuous term. Overall, we examined whether the combined effect of both the presence of an IQR increase in ambient air pollutant exposure and presence of high NEVI is greater than the sum of these individual effects on adverse COVID-19 morbidities, suggesting synergistic interaction between ambient air pollution exposure and neighborhood environmental vulnerability in influencing risk of adverse COVID-19 morbidities. We estimated the relative excess risk due to interaction (RERI), and a RERI greater than 0, yielding statistical significance at an alpha of 0.05, suggested synergistic interaction. All models were adjusted for age, sex, BMI, smoking status, asthma, diabetes, and hypertension. Analyses were conducted from March to June 2020, within areas of greater hospital catchment.

#### 2.6.4. Selection bias analyses

Selection bias could have occurred in that patients may seek treatment at specific hospitals, based upon the severity of their illness, even if they lived outside of that hospital’s typical catchment area. To account for this, we obtained total hospitalization counts per ZIP Code for NYC from the NYC DOHMH (Montesano et al., 2021). We then restricted our analyses to only ZIP Codes where at least 40 % of the total hospitalized cases were included within the INSIGHT harmonized data repository. The selection of 40 % enabled adequate sample size while also reducing the potential for selection bias. All analyses were repeated with this restricted population, identified as areas of greater hospital catchment.

To perform a more thorough analysis of selection bias, we used the R package AscRtain developed by Griffith et al. (2020) and Hemani and Using (2020) to simulate the set of selection effects that would have to occur to give rise to the observed effect estimate, under a true null effect. First, we assumed our sample was approximately 11 % of the target population. Approximately 26.6 % of cases were hospitalized during the first phase of the pandemic in NYC (Thompson et al., 2020), and we used estimates only from areas where we knew that we had at least 40 % of the total inpatient population (i.e. 26.6 % * 40 % ~ 11 %). Accordingly, we used the risk ratio (RR) that we observed for ARDS associated with the highest quartile of NO_2_ exposure in categorical analyses within the Phase 1, 40 % subsample, as our effect estimate. Given that the exposure had to be binary, we used the highest quartile and assumed 25 % of the population of NYC was exposed (quartiles were created from the full NYC population data, not only within our analytic sample). We estimated a COVID-19 prevalence of 5 %, based on estimates during Phase 1 of the pandemic (Thompson et al., 2020). We then allowed the selection effects to vary differentially in either direction (i.e., we could have greater or lower selection risk than baseline based on exposure and outcome). We allowed for the interaction between exposure and outcome on selection to again vary in either direction by 10 % and allowed the baseline probability of selection into the sample to range from 0 to 20 %. We simulated approximately 5.7 million parameter combinations.

To assess selection bias related to COVID-19 testing access and use, we stratified our data by the ZIP Code level testing rate, as reported by the NYC DOHMH and then repeated analyses for length of stay within each tertile stratum of testing rate, given the greater statistical power available for the length of stay analyses. Given that the testing data were only available for the time period that overlaps with Phases 2 and 3, we performed this sensitivity analysis within data from this time period.

## 3. Results

### 3.1. Sociodemographic, health, and environmental characteristics of the study population

Our study population, overall and stratified by phase, is summarized in Table 1. The study population represents the racial/ethnic diversity of NYC (QuickFacts: New York City, New York: United States Census Bureau, 2021). COVID-19 patients in Phase 1 were more likely to be male, Black, and have greater incidence of COVID morbidity than in Phases 2 and 3. The exception was pneumonia, which was more common among individuals hospitalized during Phases 2 and 3 of the pandemic.

**Fig. 1.**
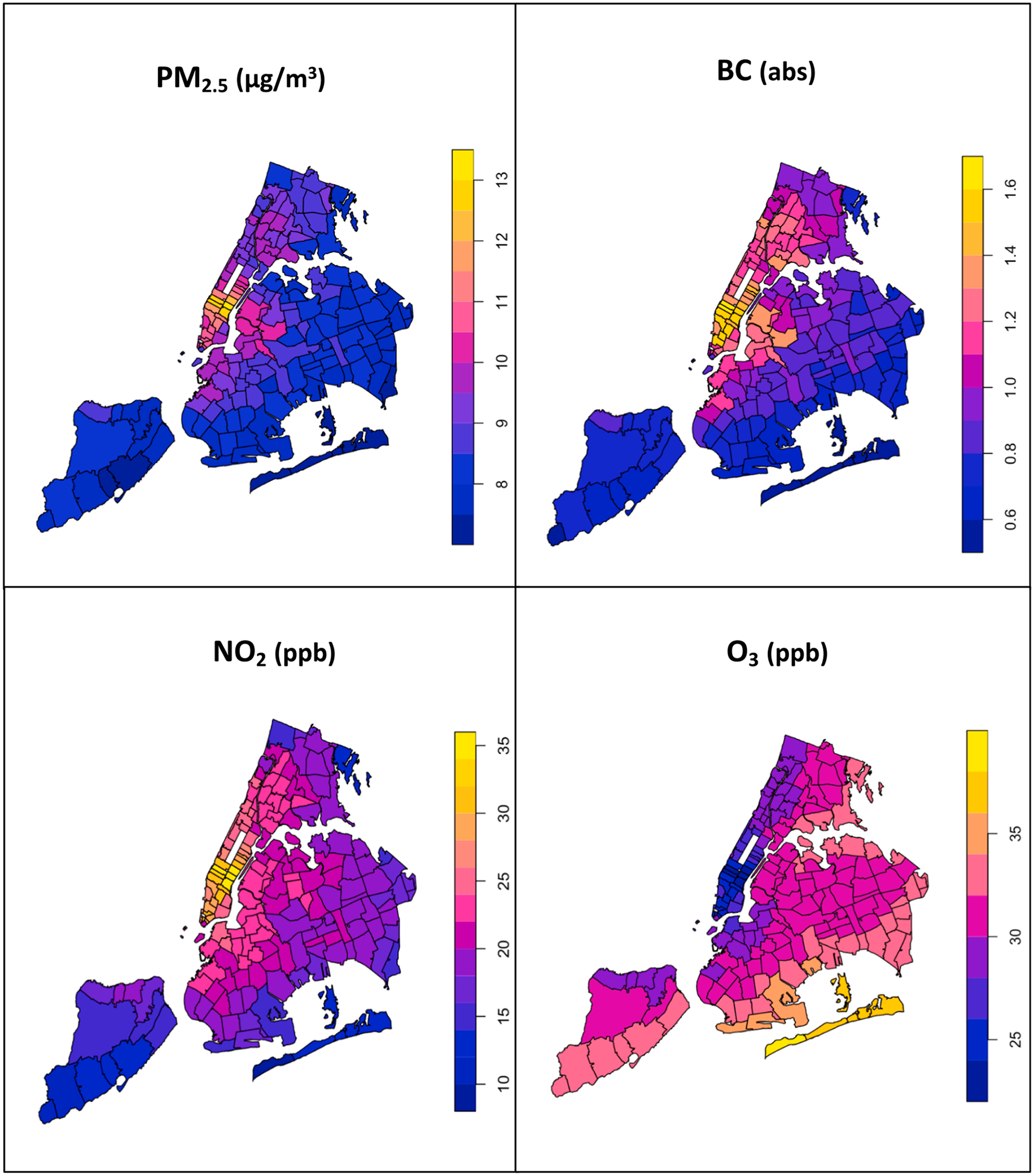
Spatial distribution of 11-year averages (2009–2019) of four air pollutants in NYC, by ZIP Code.^1 1^Abbreviations: NEVI – Neighborhood Environmental Vulnerability Index; BMI – body mass index; PM_2.5_ – fine particulate matter; BC – black carbon; NO_2_ – nitrogen dioxide; O_3_ – ozone; μg/m^3^ – micrograms per cubic meter; abs – absorbance units; ppb – parts per billion.

**Fig. 2.**
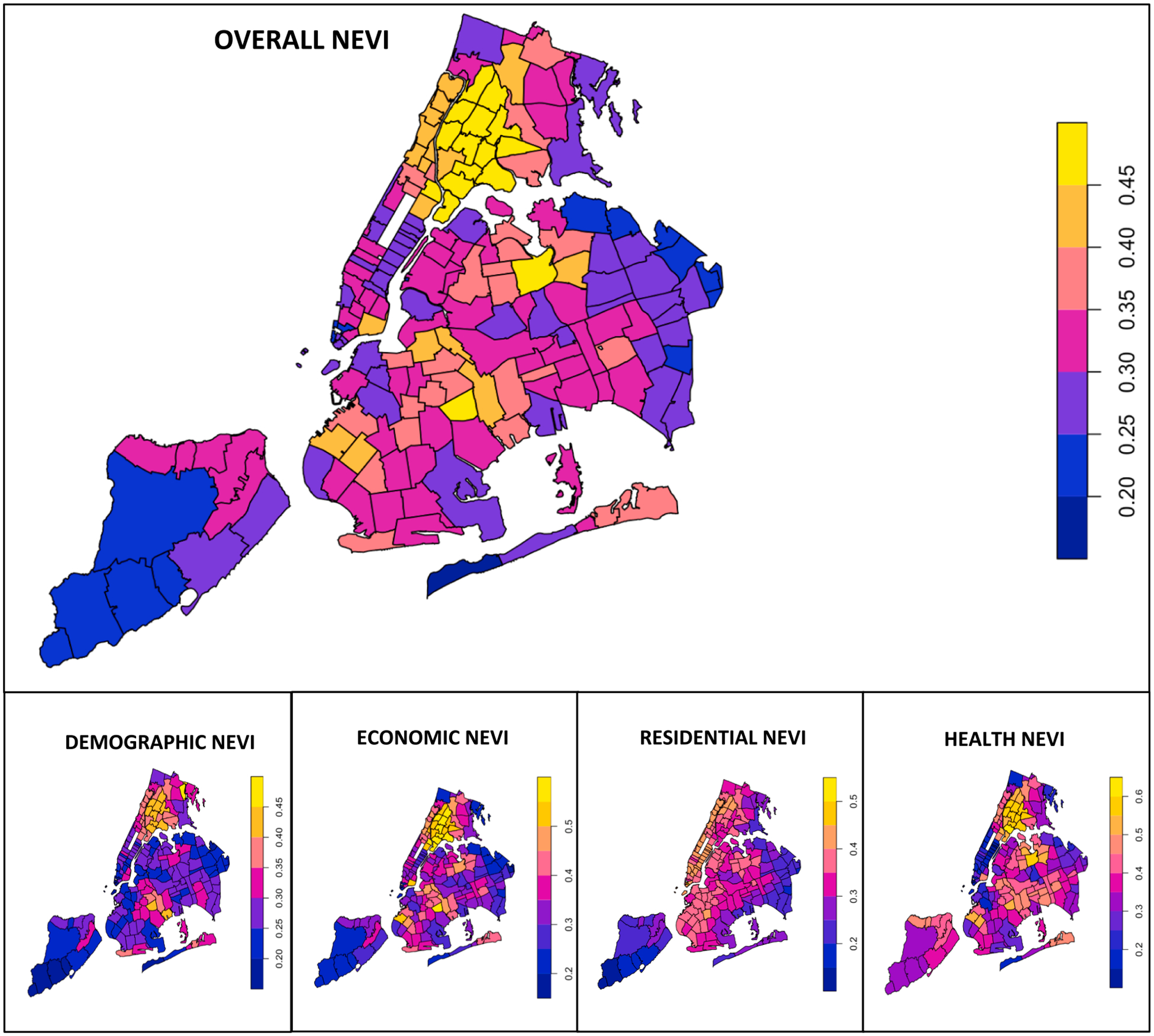
Spatial distribution of Neighborhood Environmental Vulnerability Index (NEVI), overall and individual domains, in NYC, by ZIP Code.^1 1^Data sources included the 2015–2019 US American Community Survey and 2020 CDC Places.

Fig. 1 demonstrates the spatial distribution of 11-year averaged (2009–2019) chronic air pollution exposure estimates pertaining to BC, PM_2.5_, NO_2_, and O_3_, across NYC, by ZIP Code. We found that there was more spatial variability in the NO_2_ measures, compared to the other pollutants. Fig. 2 demonstrates the spatial distribution of overall NEVI scores and domain-specific NEVI scores across NYC, by ZIP Code. We found that there was similar spatial variability across the overall NEVI and domain-specific NEVI scores. We additionally examined the correlation between the air pollutant and NEVI metrics in Supplement Fig. 1. We found that the pollutants were strongly correlated, as the magnitude of the Spearman rank correlation coefficients between the ambient air pollutants was greater than 0.8. We found that while BC, PM_2.5_ and NO_2_ were positively correlated, O_3_ was consistently inversely associated with the other pollutants. The Spearman correlation coefficient estimates between the air pollutants and the overall NEVI score and its individual domains tended to be low, as the estimates were generally less than 0.3.

### 3.2. Air pollution and adverse COVID-19 morbidity analyses

#### 3.2.1. Length of hospital stay analyses

**Fig. 3.**
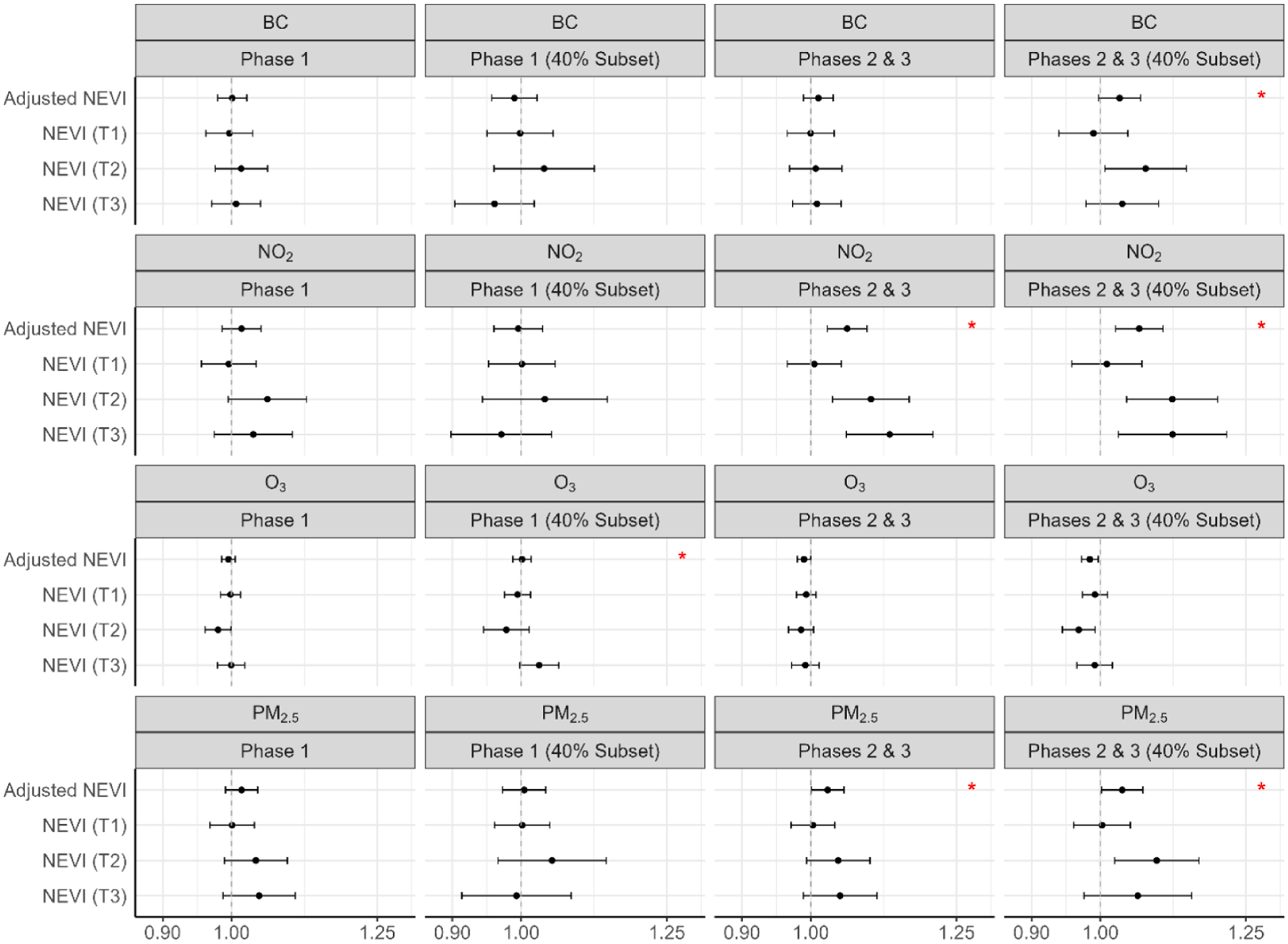
Chronic air pollution exposure (2009–2019) and length of hospital stay, stratified by NEVI and phase.^1–2 1^Cox proportional hazards regression model adjusted for age, sex, body mass index (BMI), smoking status, asthma, diabetes and hypertension; red stars identify interaction between pollutant and NEVI (alpha < 0.05) (Adhikari et al., 2020). ^2^Abbreviations: PM_2.5_ – fine particulate matter; BC – black carbon; NO_2_ – nitrogen dioxide; O_3_ – ozone; NEVI – Neighborhood Environmental Vulnerability Index; T1/T2/T3 – NEVI tertile strata with T1 as the lowest; Phase 1 – 3/2020–6/2020; Phases 2 & 3 – 7/2020–2/2021; 40 % Subset – greater hospital catchment.

Fig. 3 shows the hazard ratios for length of stay associated with each pollutant, by phase and in the subpopulation living in areas with 40 % coverage of total COVID-19 hospitalizations. The estimates regarding the relationship between length of stay and chronic air pollutant exposure, stratified by phase and NEVI tertile, were mostly null in Phase 1. In Phases 2 and 3, there was evidence that greater chronic exposure to NO_2_ and PM_2.5_ is associated with shorter length of stays (i.e. larger HRs) (NO_2_: 1.13 (1.06–1.21); PM_2.5_: 1.05 (0.99–1.11)), within the highest tertile of NEVI. See Supplement Table 5 for numerical estimates.

#### 3.2.2. ARDS analyses

**Fig. 4.**
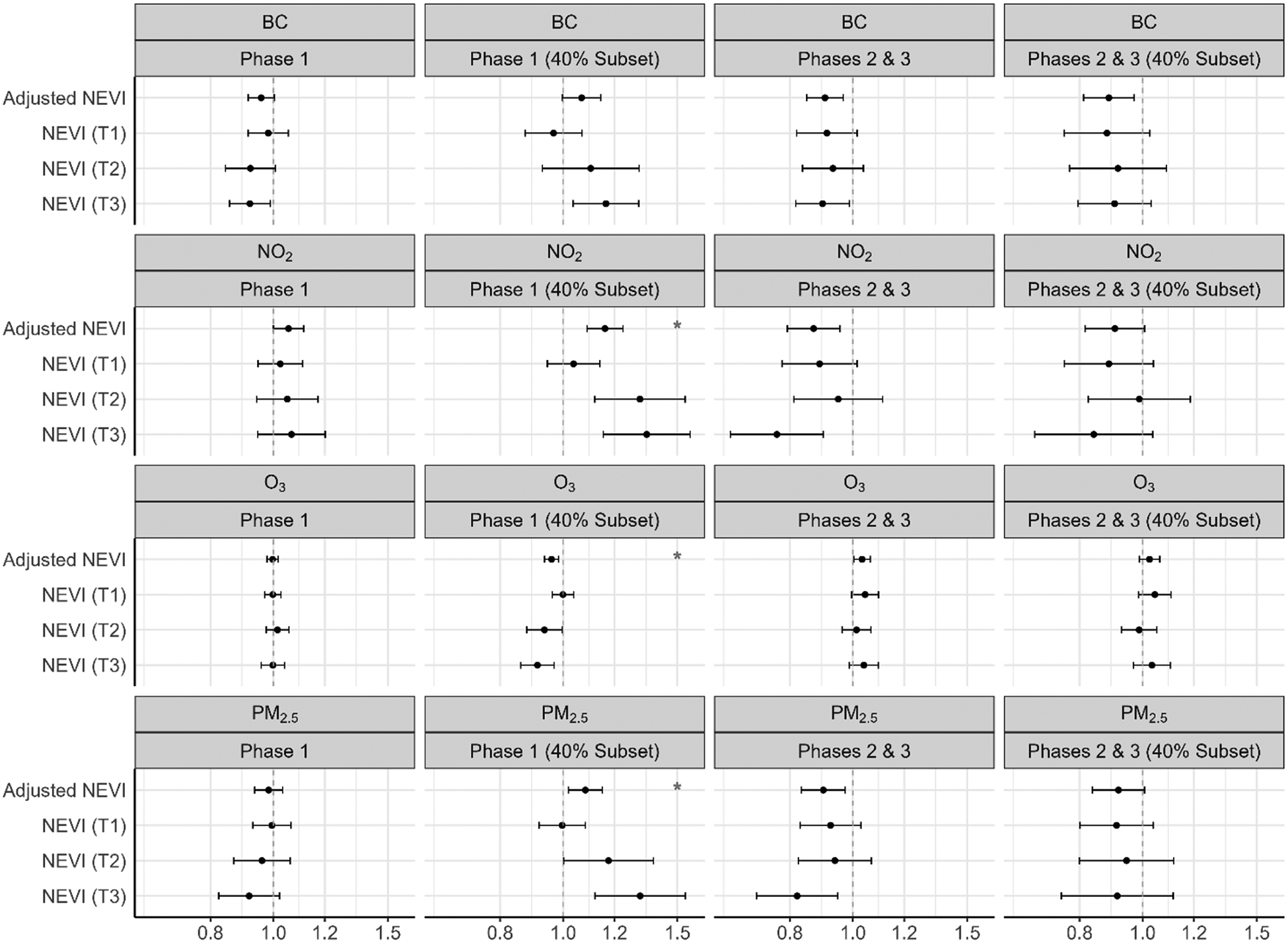
Chronic air pollution exposure (2009–2019) and ARDS risk, stratified by NEVI and phase.^1–2 1^Modified Poisson regression model adjusted for age, sex, body mass index (BMI), smoking status, asthma, diabetes and hypertension; red stars identify interaction between pollutant and NEVI (alpha < 0.05). ^2^Abbreviations: ARDS – acute respiratory distress syndrome; PM_2.5_ – fine particulate matter; BC – black carbon; NO_2_ – nitrogen dioxide; O_3_ – ozone; NEVI – Neighborhood Environmental Vulnerability Index; T1/T2/T3 – NEVI tertile strata with T1 as the lowest; Phase 1 – 3/2020–6/2020; Phases 2 & 3 – 7/2020–2/2021; 40 % Subset – greater hospital catchment.

Fig. 4 shows the risk ratios for ARDS associated with each pollutant, by phase and in the subpopulation living in areas with 40 % coverage of total COVID-19 hospitalizations. Within Phase 1 (40 % coverage), greater chronic exposure to BC, NO_2_, and PM_2.5_ was associated with an elevated risk of ARDS (BC: 1.16 (1.04–1.31); NO_2_: 1.35 (1.15–1.57); PM_2.5_: 1.31 (1.12–1.54)), and greater chronic exposure to O_3_ was associated with a reduced risk of ARDS (O_3_: 0.91 (0.86–0.97)), within the highest tertile of NEVI. Associations with O_3_ were inverse that of the other pollutants. Within Phases 2 and 3, results were generally null. Interactions between air pollutants and NEVI were statistically significant (p < 0.05) for NO_2_ (Phase 1 – 40 % Subset), O_3_ (Phase 1 – 40 % Subset), and PM_2.5_ (Phase 1 – 40 % Subset). The greatest estimated effect sizes of air pollutants (PM_2.5_, BC, and NO_2_) on ARDS risk were within the highest tertiles of NEVI. See Supplement Table 6 for numerical estimates.

#### 3.2.3. Pneumonia analyses

**Fig. 5.**
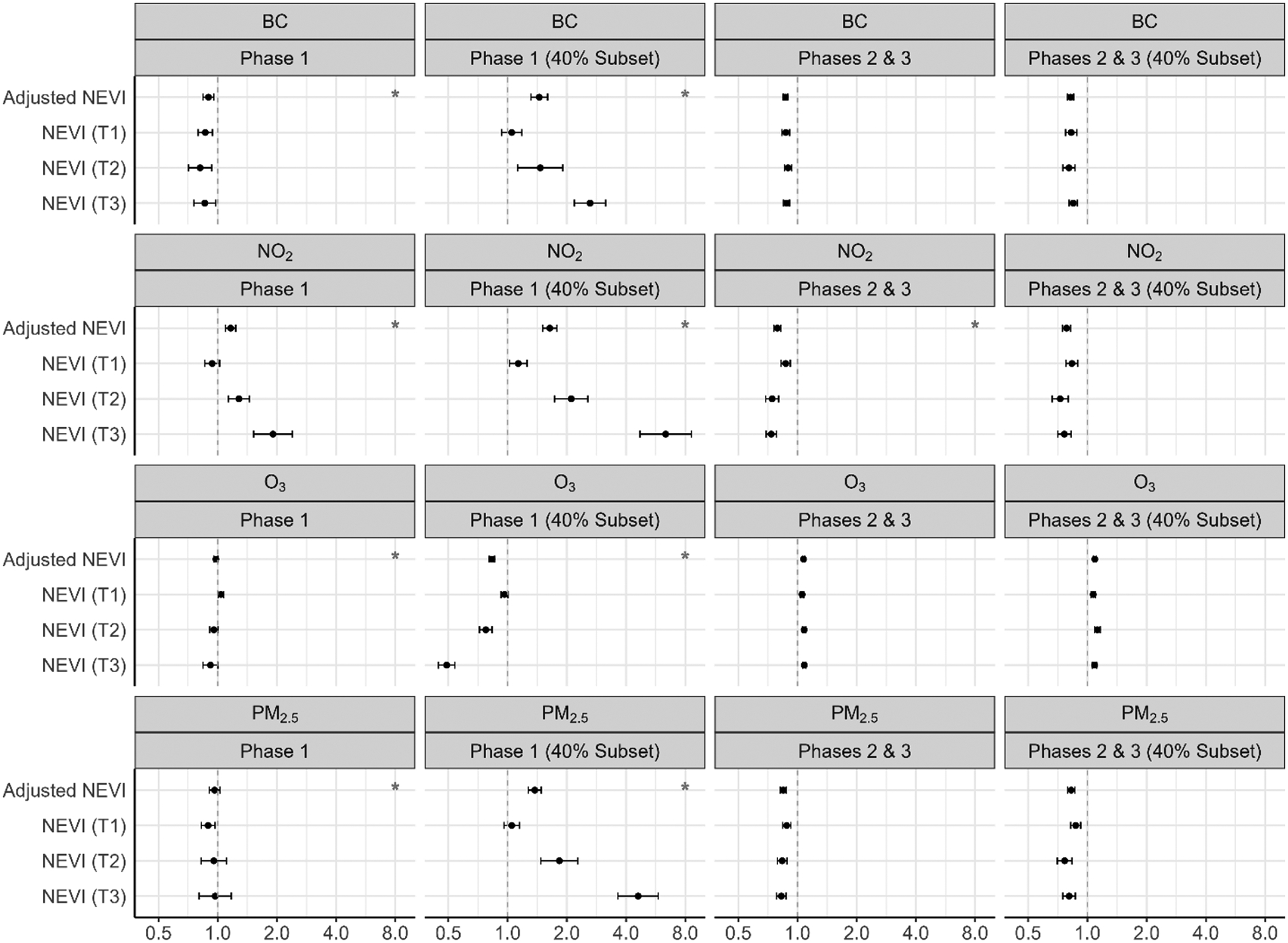
Chronic air pollution exposure (2009–2019) and pneumonia risk, stratified by NEVI and phase.^1–2 1^Modified Poisson regression model adjusted for age, sex, body mass index (BMI), smoking status, asthma, diabetes and hypertension; red stars identify interaction between pollutant and NEVI (alpha < 0.05). ^2^Abbreviations: PM_2.5_ – fine particulate matter; BC – black carbon; NO_2_ – nitrogen dioxide; O_3_ – ozone; NEVI – Neighborhood Environmental Vulnerability Index; T1/T2/T3 – NEVI tertile strata with T1 as the lowest; Phase 1 – 3/2020–6/2020; Phases 2 & 3 – 7/2020–2/2021; 40 % Subset – greater hospital catchment *Mechanical Ventilation Use Analyses*.

Fig. 5 shows the risk ratios for COVID-19 pneumonia associated with each pollutant, by phase and in the subpopulation living in areas with 40 % coverage of total COVID-19 hospitalizations. In Phase 1 (full population), greater chronic exposure to NO_2_ was associated with an elevated risk of pneumonia, within the highest NEVI tertile (NO_2_: 1.91 (1.52–2.40)). Within Phase 1 (40 % coverage), greater chronic exposure to BC, NO_2_, and PM_2.5_ was associated with an elevated risk of pneumonia (BC: 2.62 (2.19–3.15); NO_2_: 6.36 (4.71–8.60); PM_2.5_: 4.61 (3.64–5.84)), and greater chronic exposure to O_3_ was associated with a reduced risk of pneumonia (O_3_: 0.49 (0.44–0.54)), within the highest tertile of NEVI. Within Phases 2 and 3 (40 % coverage), greater chronic exposure to BC, NO_2_, and PM_2.5_ was associated with a reduced risk of pneumonia (BC: 0.85 (0.81–0.89); NO_2_: 0.76 (0.71–0.83); PM_2.5_: 0.81 (0.75–0.87)), and greater chronic exposure to O_3_ was associated with an increased risk of pneumonia (O_3_:1.09 (1.06–1.11)), within the highest tertile of NEVI. There was evidence that the relationship between chronic air pollution exposure and COVID-19 pneumonia yielded the highest effect sizes in areas of greater neighborhood environmental vulnerability, with interactions between air pollutants and NEVI being statistically significant (p < 0.05) for BC (Phase 1; Phase 1 – 40 % Subset), NO_2_ (Phase 1; Phase 1 – 40 % Subset; Phases 2 & 3), O_3_ (Phase 1; Phase 1 – 40 % Subset), and PM_2.5_ (Phase 1; Phase 1 – 40 % Subset). See Supplement Table 7 for numerical estimates.

#### 3.2.4. Ventilation use analyses

**Fig. 6.**
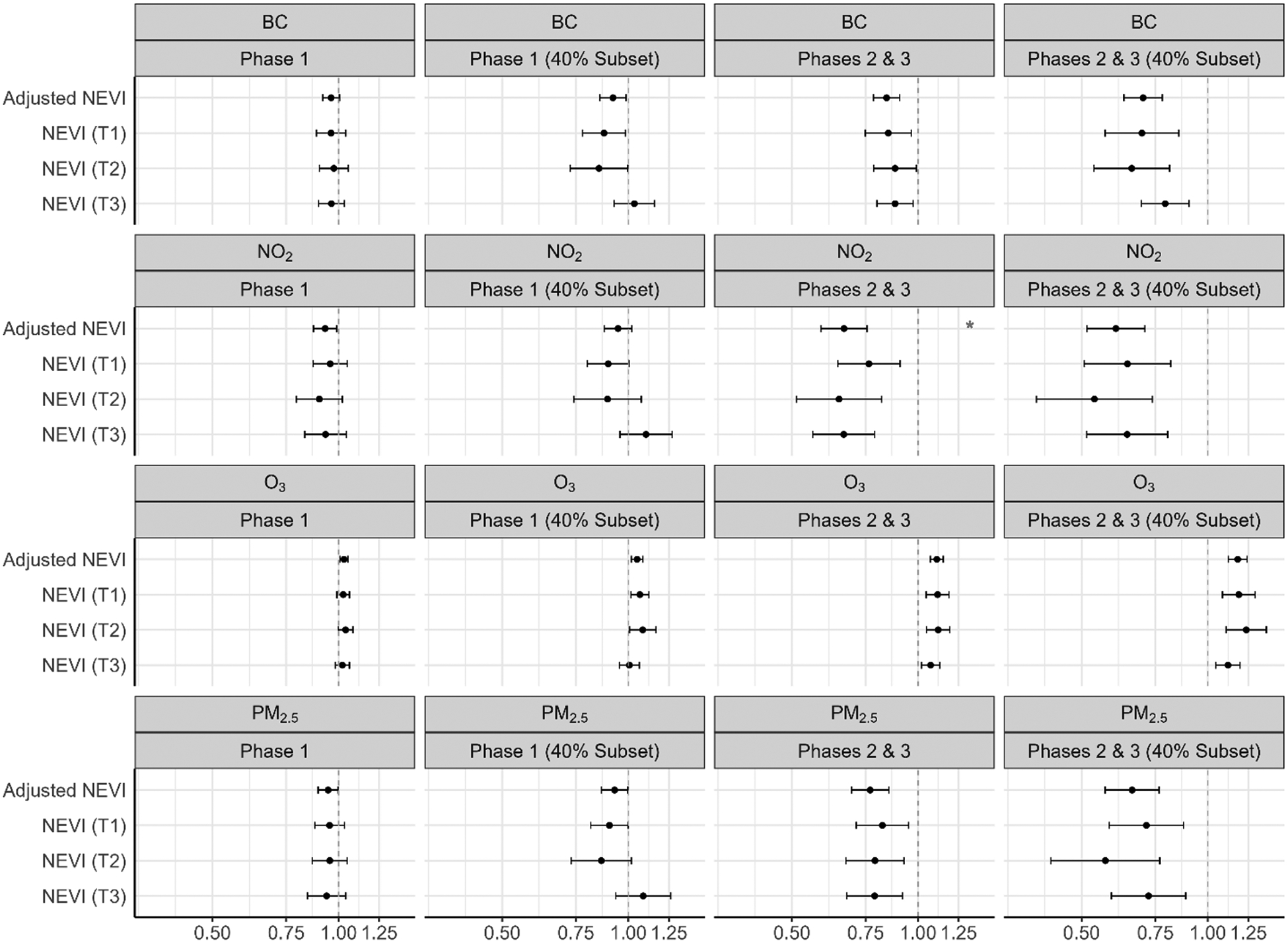
Chronic air pollution exposure (2009–2019) and ventilation risk, stratified by NEVI and phase.^1−2 1^ Modified Poisson regression model adjusted for age, sex, body mass index (BMI), smoking status, asthma, diabetes and hypertension; red stars identify interaction between pollutant and NEVI (alpha < 0.05). ^2^Abbreviations: PM_2.5_ – fine particulate matter; BC – black carbon; NO_2_ – nitrogen dioxide; O_3_ – ozone; NEVI – Neighborhood Environmental Vulnerability Index; T1/T2/T3 – NEVI tertile strata with T1 as the lowest; Phase 1 – 3/2020–6/2020; Phases 2 & 3 – 7/2020–2/2021; 40 % Subset – greater hospital catchment.

Fig. 6 shows the risk ratios for mechanical ventilation use associated with each pollutant, by phase and in the subpopulation living in areas with 40 % coverage of total COVID-19 hospitalizations. Within Phase 1 and Phase 1 (40 % coverage), results were generally null. Within Phases 2 and 3 (40 % coverage), greater chronic exposure to NO_2_, BC and PM_2.5_ was associated with reduced risk of mechanical ventilation use (BC: 0.79 (0.69–0.90); NO_2_: 0.64 (0.51–0.80); PM_2.5_: 0.72 (0.59–0.89)), and greater chronic exposure to O_3_ was associated with greater risk of mechanical ventilation use (O_3_: 1.12 (1.05–1.19)), within the highest tertile of NEVI. Effect estimates of greater magnitude were observed in Phases 2 and 3, in comparison to Phase 1. We saw minimal evidence of interaction between air pollutants and NEVI, with interaction being marginally significant (p ~ 0.05) for NO_2_ during Phases 2 and 3. See Supplement Table 8 for numerical estimates.

#### 3.2.5. Dialysis use analyses

**Fig. 7.**
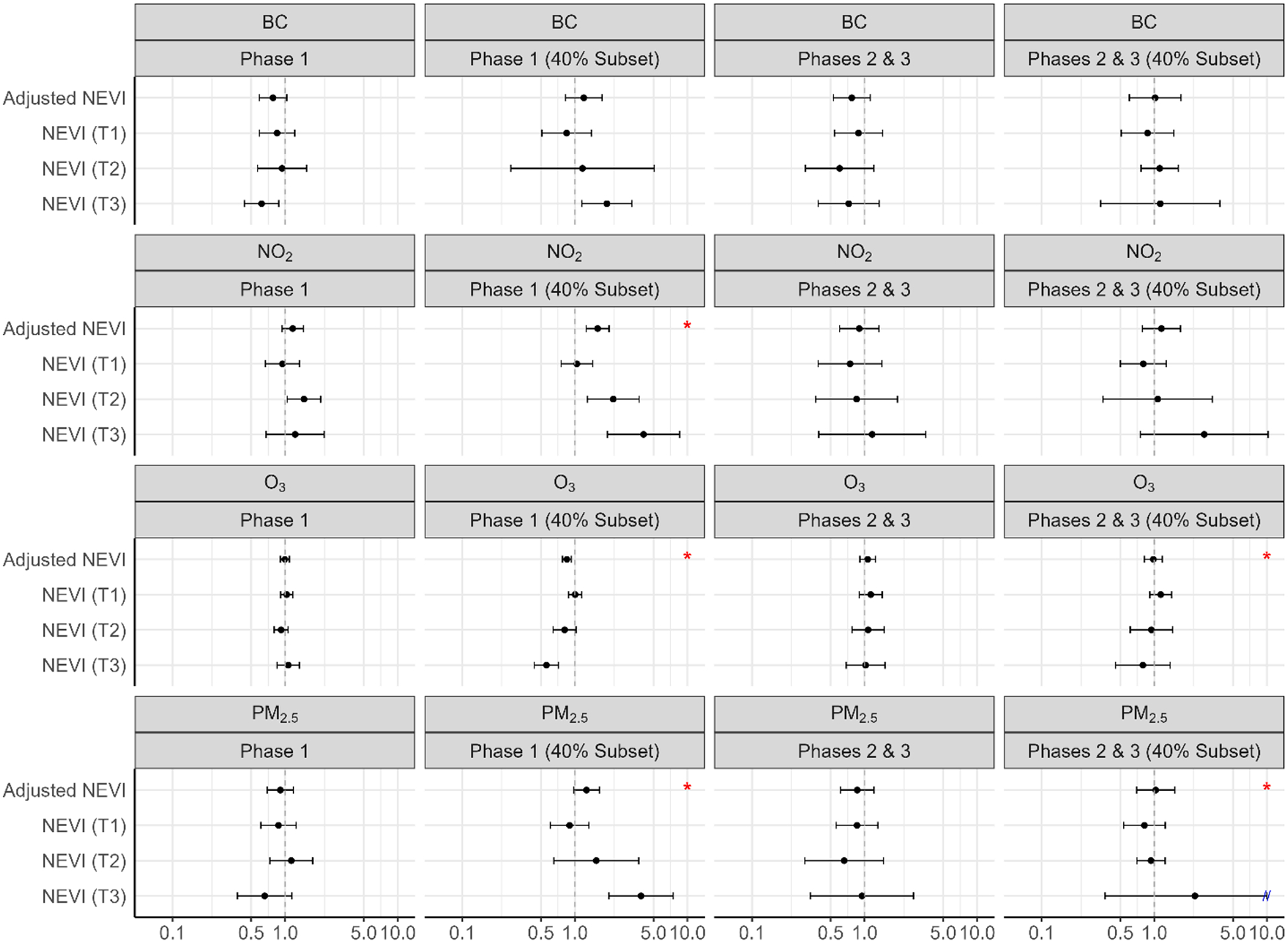
Chronic air pollution exposure (2009–2019) and dialysis risk, stratified by NEVI and phase.^1–3 1^ Modified Poisson regression model adjusted for age, sex, body mass index (BMI), smoking status, asthma, diabetes and hypertension; red stars identify interaction between pollutant and NEVI (alpha < 0.05). ^2^Abbreviations: PM_2.5_ – fine particulate matter; BC – black carbon; NO_2_ – nitrogen dioxide; O_3_ – ozone; NEVI – Neighborhood Environmental Vulnerability Index; T1/T2/T3 – NEVI tertile strata with T1 as the lowest; Phase 1 – 3/2020–6/2020; Phases 2 & 3 – 7/2020–2/2021; 40 % Subset – greater hospital catchment. ^3^Population was restricted to those with no prior dialysis, within the 11 years in the INSIGHT EHR data.

Fig. 7 shows the risk ratios for dialysis use associated with each pollutant, by phase and in the subpopulation living in areas with 40 % coverage of total COVID-19 hospitalizations. Within Phase 1 (40 % coverage), greater chronic exposure to BC, NO_2_, and PM_2.5_ was associated with an elevated risk of dialysis (BC: 2.64 (1.78–3.90); NO_2_: 7.02 (3.76–13.08); PM_2.5_: 5.80 (3.51–9.58)), and greater chronic exposure to O_3_ was associated with a reduced risk of dialysis (O_3_: 0.49 (0.40–0.59)), within the highest tertile of NEVI. Within Phases 2 and 3 (40 % coverage), greater chronic exposure to BC, NO_2_, and PM_2.5_ was associated with a greater risk of dialysis (BC: 1.94 (0.82–4.60); NO_2_: 4.15 (1.50–11.48); PM_2.5_: 3.08 (0.95–9.97)), and greater chronic exposure to O_3_ was associated with a reduced risk of dialysis (O_3_: 0.66 (0.45–0.96)), within the highest tertile of NEVI. Wider confidence intervals occurred due to small sample size for dialysis use. There was evidence that the relationship between chronic air pollution exposure and dialysis use yielded the highest effect sizes in areas of greater NEVI, with interactions between air pollutants and NEVI being statistically significant (p < 0.05) for NO_2_ (Phase 1 – 40 % Subset), O_3_ (Phase 1 – 40 % Subset; Phases 2 & 3 – 40 % Subset), and PM_2.5_ (Phase 1 – 40 % Subset; Phases 2 & 3 – 40 % Subset). See Supplement Table 9 for numerical estimates.

### 3.3. Sensitivity analyses

#### 3.3.1. Two-pollutant analyses

Constructing two-pollutant models for the relationship between BC, NO_2_, and PM_2.5_ and adverse COVID-19 morbidities, while simultaneously adjusting for O_3_, did not consistently change conclusions from single pollutant analyses. An exception was found in the relationship between chronic air pollution exposure and mechanical ventilation use. In single-pollutant models, we found that the relationship between chronic NO_2_, BC, and PM_2.5_ exposure and mechanical ventilation use was generally null in Phase 1. In two-pollutant models that adjusted for chronic O_3_ exposure, we found that the effect estimates for use of mechanical ventilation shifted to the other side of the null during Phase 1. For example, after adjusting for chronic O_3_ exposure, an increase in chronic NO_2_ exposure was associated with an increased adjusted risk of mechanical ventilation use (RR: 1.33 95 % CI: 1.11, 1.60). See Supplement Table 10 for numeric estimates for two-pollutant analyses.

#### 3.3.2. Racial/ethnic subgroup analyses

We only observed racial and ethnic disparities with Black populations and Hispanic populations having higher risk of COVID-19 pneumonia associated with greater pollutant exposure, after adjusting for NEVI. Formal effect measure modification analyses to compare Black and White populations, and to compare Hispanic and non-Hispanic populations demonstrated statistically significant differences across pollutants (p < 0.05). See Supplement Table 11 for numeric estimates for racial/ethnic subgroup analyses regarding the relationship between chronic air pollution exposure and risk of COVID-19 pneumonia, from March to June 2020 (within areas of greater hospital catchment). We did not find strong evidence of racial and ethnic disparities after adjusting for neighborhood environmental vulnerability, for the remaining adverse COVID-19 morbidities, including length of stay, ARDS, ventilation, and dialysis.

#### 3.3.3. Additive interaction analyses

In examining additive interaction between chronic air pollution exposure and neighborhood environmental vulnerability, we found evidence of synergistic interaction (p < 0.05). We found that the combined effect of an IQR increase in PM_2.5_ exposure and presence of a high NEVI score (i.e., upper NEVI tertile) on the risk of ARDS was greater than the sum of the individual effects. We similarly found that the combined effect of an IQR increase in BC, PM_2.5_, or NO_2_ exposure and presence of a high NEVI score (i.e., upper NEVI tertile) on the risk of dialysis use, pneumonia, and ventilation use was greater than the sum of the individual effects. See Supplement Table 12 for numeric estimates for RERI analyses between chronic air pollution exposure and neighborhood environmental vulnerability for risk of adverse COVID-19 morbidities, from March to June 2020 (within areas of greater hospital catchment).

#### 3.3.4. Selection bias analyses

As described in the methods, we used an available R package and corresponding SHINY app to assess the range of selection factors that could explain our results as a sensitivity analysis. We used 1.5 as our target estimate, the RR that we observed for ARDS associated with the highest quartile of NO_2_ exposure in categorical analyses within the Phase 1, 40 % coverage subsample. Supplement Fig. 2 demonstrates the results of this analysis. Of all the parameter combinations aligned with our data, approximately 22 % could explain our result of an RR of 1.5 through selection (n = 22,953/104,161). As shown in the plot, we see the plausible combinations of selection effects that could produce our estimate mainly occur when selection into the sample among the exposed is lower than baseline selection (i.e. *β*_*A*_ is negative). As evidenced by the colors of the dots, the plausible combinations when the selection into the sample is lower among the exposed primarily occurs when the baseline probability is greater than 10 %. Increasing the target risk ratio to align with the values that we observed with pneumonia or dialysis causes a smaller percentage of parameter combinations to plausibly explain our results, although the probable combinations remain as those where selection into the study population among the exposed is considerably lower than baseline.

We additionally conducted sensitivity analyses by testing rate. We stratified the population by the testing rate in their ZIP Code and reran a subset of length of stay analyses for Phases 2 and 3. We did not observe considerable differences in results (Supplement Table 13). We also did not observe considerable correlation between testing rate and average air pollution metrics for NYC Zip Codes, with Spearman correlation coefficients ranging from − 0.2 with O_3_ to 0.2 with PM_2.5_.

## 4. Discussion

Overall, we observed that chronic exposure to air pollutants BC, NO_2_, and PM_2.5_ was associated with greater risk of severe COVID-19 morbidities, such as ARDS, pneumonia and dialysis, but had no association with risk of mechanical ventilation and longer length of stay during the initial wave of the pandemic (3/2020–6/2020). In the latter wave (7/2020–2/2021), chronic BC, NO_2_, and PM_2.5_ exposure was not associated with adverse COVID-19 morbidities, except for mechanical ventilation, where chronic air pollution exposure was associated with reduced risk of ventilation use. Opposite results were consistently observed for O_3_, due to ozone’s strong negative correlations with the other pollutants. The relationship between chronic air pollution exposure and COVID-19 morbidities, including ARDS, pneumonia, and dialysis use, were stronger among individuals living in areas of higher neighborhood social and structural vulnerability. There was evidence of multiplicative interaction between chronic air pollution exposure and neighborhood environmental vulnerability for risk of ARDS, pneumonia, and dialysis use. There was additionally evidence of additive interaction between chronic air pollution exposure and neighborhood environmental vulnerability for risk of ARDS, pneumonia, ventilation use, and dialysis use. The relationship between chronic air pollution exposure and adverse COVID-19 morbidities was generally stronger among areas of greater neighborhood environmental vulnerability within the first wave of the pandemic, compared to subsequent pandemic periods. Overall, this study demonstrates that the effect of air pollution on COVID-19 morbidity is often greater in magnitude within communities affected by multiple social and structural factors.

Our results are consistent with prior literature on demonstrating positive associations between chronic air pollution exposure and COVID-19 morbidity. The epidemiological literature to date broadly suggests that greater air pollution exposure is associated with greater risk of adverse COVID-19 morbidity on an individual-level (Bozack et al., 2022; Lopez-Feldman et al., 2021; Bowe et al., 2021; Chen et al., 2022; Mendy et al., 2021; Beloconi and Vounatsou, 2023; Chen et al., 2021; Pegoraro et al., 2021; Lavigne et al., 2023; Hyman et al., 2023; Chen et al., 2022; English et al., 2022; Tian et al., 2021; Jerrett et al., 2023; Sheridan et al., 2022; Li et al., 2022). Zhang et al. (2023) used the National COVID-19 Surveillance System in Denmark, and found that chronic exposure to PM_2.5_ and NO_2_ is associated with greater risk of hospital admission (Zhang et al., 2023). Bozack et al. (2022) additionally used COVID-19 hospitalization data in NYC from March 8 to August 30, 2020, and found that chronic exposure to PM_2.5_ was associated with greater risk of ICU admission (Bozack et al., 2022). To our knowledge, there have been no studies that examined the relationship between chronic air pollution exposure and the use of mechanical ventilation after COVID-19 diagnosis; thereby, limiting comparison. De Weerdt et al. (2020) found some evidence that ambient air pollution exposure was associated with increased duration of mechanical ventilation use (De Weerdt et al., 2020). In regards to comparing our study time frame, we examined the initial wave of the pandemic and subsequent phases of the pandemic, before COVID-19 vaccines were publicly available. Similarly, our study was conducted within NYC, one of the geographies with an early and severe peak of COVID-19 followed by fewer cases in the summer and early fall months before the second peak in the winter 2020/2021 time period. Previous research have established that the pandemic patterns in NYC differed from the rest of the US (Pei et al., 2021), as Pei et al. (2021) estimated that NYC had lower population susceptibility following the initial peak (Pei et al., 2021).

In examining our study findings, we found different patterns across pollutants, adverse COVID-19 morbidities, and phases. Firstly, regarding study findings across pollutants, examining O_3_ exposure consistently provided opposite relationships than those observed for the other three pollutants (NO_2_, PM_2.5_, BC). Other studies have found similar patterns, both in COVID-19 related research and in other outcomes when examining health risks associated with air pollution (Zhang et al., 2023; Clougherty et al., 2021). In NYC, chronic O_3_ exposure is strongly and negatively correlated with the other pollutants (Clougherty et al., 2021). This occurs in that emissions from vehicular traffic, industry plants, and other sources of ambient air pollution exposure may contribute higher levels of NO (nitrogen oxide). NO reacts with ground-level O_3_ to create NO_2_ (nitrogen dioxide); in this case, the formation of nitrogen dioxide is linked to ground-level ozone depletion. In contrast, in the presence of sunlight, especially during the summer months, nitrogen dioxide and volatile organic compounds (emitted from ambient air pollution exposure sources) chemically react to form ground-level ozone. Therefore, the inverse relationship between NO_2_ and O_3_ concentration levels explain the inverse findings when comparing the respective relationships between NO_2_ and adverse COVID-19 risk to O_3_ and adverse COVID-19 risk (Zhang et al., 2023; Clougherty et al., 2021). We constructed two-pollutant models for the relationship between BC, NO_2_, and PM_2.5_ and adverse COVID-19 morbidities, while simultaneously adjusting for O_3_, to assess competing effects between pollutants, and we found that this did not consistently change conclusions from single pollutant analyses. Secondly, regarding study findings across adverse COVID-19 morbidities, we generally found that chronic air pollution exposure was associated with greater risk of adverse COVID-19 morbidities, except for longer length of stay and mechanical ventilation use. Regarding length of hospital stay, the analyses generally suggest that chronic air pollution exposure is not associated with length of stay for COVID-19 hospitalization. Regarding the relationship between chronic air pollution exposure and mechanical ventilation, we similarly found the association was null in Phase 1, and in Phases 2 & 3, we found that greater chronic air pollution exposure was associated with reduced risk of ventilation use. In explaining the findings, it is important to note that there was reduced risk of ventilation use in the latter phases, with approximately 8 % of the sample experiencing ventilation, as opposed to 16 % in Phase 1. Similarly, in the latter phases, there may have been supplementary factors at play in influencing the risk of ventilation use, such as individual engagement in masking and social distancing, and greater healthcare capacity, which may have similarly contributed to inverse associations between chronic air pollution exposure and ventilation use. Thirdly, regarding study findings across phases, in Phase 1 (full population and within hospital catchment), there is evidence that the relationship between chronic air pollution exposure and risk of ARDS and pneumonia is stronger among populations with greater neighborhood environmental vulnerability. In contrast, in Phases 2 & 3 (full population and within hospital catchment), there is no clear evidence that the relationship between chronic air pollution exposure and risk of ARDS and pneumonia is stronger among populations with greater neighborhood environmental vulnerability. These contradictory findings may occur, given that in the latter half of 2020, there similarly may be additional factors at play, in influencing the risk of adverse COVID-19 morbidities. These factors may include individual masking and social distancing behaviors. In addition, NYC was identified as the global COVID-19 epicenter from March to June 2020, exhibiting greater adverse COVID-19 burden, as opposed to the latter phases. In Phases 2 & 3, there was reduced adverse COVID-19 morbidity. Reduced adverse COVID-19 morbidity may have occurred, due to a harvesting effect in that those that experienced adverse COVID-19 morbidity in Phase 1 are less likely to experience adverse COVID-19 morbidity in Phases 2 & 3. As a result, there was a low adverse COVID-19 burden in Phases 2 & 3 as opposed to Phase 1, and thus, the relationships between chronic air pollution exposure, adverse COVID-19 morbidity, and neighborhood environmental vulnerability may have been more difficult to extrapolate.

Overall, it is important to examine whether the relationships between air pollution and adverse COVID-19 morbidities are modified by area-level vulnerability due to social and structural factors. To our knowledge, previous studies have often adjusted for individual social and structural factors, such as population-level median income (Chen et al., 2022), population density (Bowe et al., 2021), and population health behaviors, as confounders (Bowe et al., 2021). Previous studies have not examined the role of population-level vulnerability, defined by neighborhood-level social and structural factors, in the relationship between chronic air pollution exposure and adverse COVID-19 morbidities. Therefore, we aimed to assess whether neighborhood environmental vulnerability (NEVI) modifies the relationship between chronic air pollution exposure and adverse COVID-19 morbidities. It is important to note that the NEVI scores are intentionally standardized to NYC, preventing comparison with other US cities. A NEVI standardized to NYC can help researchers identify disinvested neighborhoods in NYC that may need more resources to mitigate environmental vulnerability, compared to other neighborhoods in NYC. Overall, the examination of effect modification by neighborhood factors enables us to better understand the effect of chronic air pollution exposure on COVID-19 morbidities. We also found that NEVI may help explain racial and ethnic disparities, as we conducted analyses in racial and ethnic subgroups after adjusting for NEVI and found no significant stratum-specific differences by race and ethnicity for adverse COVID-19 morbidities, except for pneumonia. Using NEVI, we can better allocate public health resources for population subgroups that may be most vulnerable to the effects of the environment on pandemic outcomes. In considering neighborhood environmental vulnerability, our study aims to fully flesh out the relationship between air pollution exposure and adverse COVID-19. In hypothesizing that neighborhood environmental vulnerability modifies the relationship between air pollution and adverse COVID-19, we propose that the effects of air pollution on adverse COVID-19 morbidity are not uniform across all populations and that neighborhood-level social and structural factors may lead an individual to be more vulnerable to the effects of chronic air pollution exposure on adverse COVID-19 morbidity. For example, poverty, overcrowding, and limited healthcare access on a community-level may lead individuals to be more susceptible to the effects of chronic air pollution exposure on health. Greater vulnerability to the effects of chronic air pollution on health may contribute to greater immune system dysregulation and worsening of existing health conditions, leading an individual to be at greater risk for adverse COVID-19 morbidity. Thus, in examining the relationship between environmental exposures and adverse health outcomes, there is an integral need to consider population-level vulnerability to these environmental exposures.

All conclusions from this research should be tempered with a thorough consideration of the limitations of the data. This analysis is restricted to hospitalized patients, introducing the potential for selection bias from a number of avenues. First, it is possible that the academic medical centers that made up our study population attracted more severe cases from areas that are typically outside of their catchment area. Our sensitivity analysis restricting the study population to only areas where at least 40 % of the total COVID-19 hospitalizations were contained in the data resource attempted to address this potential bias. We did observe differences in results, as we typically observed stronger effect estimates within the restricted population. Selection bias could also arise from restricting to hospitalized patients, essentially conditioning on a collider if air pollution not only increased the risk of the adverse COVID-19 morbidity, but also increased the risk of COVID-19 incidence (Griffith et al., 2020; Banack et al., 2018). As suggested by prior literature, we used the AscRtain package in R to run a series of simulations to determine the selection factors that could be needed to produce our observed results (Griffith et al., 2020; Hemani and Using, 2020). Generally, we observed that selection bias was more probable if the risk of hospitalization among those exposed to greater air pollution was lower than the baseline risk. Individuals from areas of higher air pollution were over-represented within our study population when compared to the total COVID-19 hospitalization data from the NYC DOHMH, suggesting that the risk of hospitalization among those exposed to greater air pollution was not lower than the baseline risk.

In addition to selection bias, the use of hospitalized patient data led to other limitations of the analysis. There were no direct measures of individual income status within the EHR data. While we did control for a comprehensive index of neighborhood-level social and structural drivers, such as neighborhood-level median income, the potential for residual confounding still exists. The harmonized EHR data also only included ZIP Code of residence, which limited the spatial resolution of air pollution estimates that could be used within the analysis. Thus, when linking with the address at admission to assign 11-year chronic air pollution exposure estimates, we did not consider if an individual previously moved, which could have led to exposure misclassification and a potential bias. Finally, we could not account for differences in hospital-based processes during the different phases of the pandemic. For example, due to the overwhelming number of admissions and the lockdowns in NYC, very few people were admitted to hospital for any other reasons than COVID-19 illness in the first phase. The later phases of the pandemic could potentially include individuals hospitalized for other primary reasons but then were diagnosed with asymptomatic COVID-19 during the admission. This could explain some of the discrepancies observed in our results between the different phases of the pandemic, along with the previously described differences in patterns between NYC and other geographic areas (Pei et al., 2021).

The use of harmonized EHR data across multiple institutions also presented a number of strengths. We had a large sample size that enabled the examination of a number of subpopulations of interest, most notably racial and ethnic minorities, and the direct examination of interaction between air pollution and neighborhood vulnerability. The greater geographic variability made possible by using data across medical centers enabled our examination of greater exposure contrasts than possible when including only a single hospital’s geographic catchment area. The ability to include records across institutions from the prior 11 years also reduced potential misclassification of chronic disease diagnoses compared to relying on the index hospitalization within a single institution’s medical records. Similarly, we were able to impute important confounders such as BMI and smoking by using historical records within the harmonized repository.

### 4.1. Implications of findings

We found that the relationship between chronic air pollution exposure and risk of adverse COVID-19 morbidities is stronger among areas of greater neighborhood-level vulnerability, and similarly, found evidence of synergistic interaction between chronic air pollution exposure and neighborhood environmental vulnerability in influencing risk of adverse COVID-19 morbidity on the additive scale. Overall, findings suggest that neighborhood-level social and structural factors can modify the effects of air pollution on adverse COVID-19 morbidity; thus, community-level environmental vulnerability should be considered when examining the relationship between chronic air pollution exposure and adverse COVID-19 morbidities. Regarding potential mechanisms underlying this relationship, neighborhood-level social and structural factors, such as low socioeconomic status, can induce chronic stress, which may affect immune responses and dysregulate gene expression; thereby, amplifying the harmful effects of ambient air pollution exposure on individual health and increasing the risk of adverse COVID-19 morbidities (Mattos Dos Santos and Isolation, 2020; Clougherty and Kubzansky, 2009). In terms of broader public health implications, our study suggests that public health resources and policies should target neighborhoods that experience greater environmental vulnerability, which may help mitigate pandemic outcomes on a community-level. Potential interventions may include air pollution reductions among communities with greater environmental vulnerability or addressing social and structural determinants, such as overcrowding, unemployment, or health behaviors, which can help mitigate environmental vulnerability. This study suggests that focusing on identifying and assisting populations that may be most vulnerable to the effects of air pollution on health can help ease population-level pandemic burden.

Regarding future research directions, pooled data that can amass larger sample sizes to improve precision and enable investigation of larger exposure contrasts may address some of the limitations of this work. In addition, future research should be conducted within cohorts that are less susceptible to the selection biases of hospitalized populations. We strongly encourage large-scale studies using harmonized data across systems powered to explicitly focus on examining the multi-way interactions between social and structural drivers of health, air pollution and COVID-19 morbidities to further investigate the inequities observed in COVID-19 morbidities and help encourage future pandemic preparedness on a population-level.

## Supplementary Material

1

Appendix A. Supplementary data

Supplementary data to this article can be found online at https://doi.org/10.1016/j.envint.2025.109660.

## Figures and Tables

**Table 1:** Demographic and health characteristics of INSIGHT hospitalization data for COVID-19, NYC 2020.^1,2,3,4^

	Overall 3/20–2/21 (n = 20,318)	Phase 1 3/20–6/20 (n = 11,652)	Phase 1 40 % Subset 3/20–6/20 (n = 7487)	Phases 2 & 3 7/20–2/21 (n = 8666)	Phases 2 & 3 40 % Subset 3/20–6/20 (n = 5600)
**Age, years**	64.2 (17.8)	63.9 (17.3)	65.1 (17.1)	64.5 (18.5)	66.0 (18.0)
**Sex**					
Male	10,673 (52.5 %)	6365 (54.6 %)	4065 (54.3 %)	4308 (49.7 %)	2813 (50.2 %)
Female	9645 (47.5 %)	5287 (45.4 %)	3422 (45.7 %)	4358 (50.3 %)	2787 (49.8 %)
**Race**					
White	5485 (27.0 %)	2876 (24.7 %)	1537 (20.5 %)	2609 (30.1 %)	1442 (25.8 %)
Black	4602 (22.6 %)	2947 (25.3 %)	2025 (27.0 %)	1655 (19.1 %)	1108 (19.8 %)
Asian	1233 (6.1 %)	538 (4.6 %)	289 (3.9 %)	695 (8.0 %)	487 (8.7 %)
American Indian & Alaskan Native	58 (0.3 %)	29 (0.2 %)	21 (0.3 %)	29 (0.3 %)	17 (0.3 %)
Native Hawaiian & Pacific Islander	47 (0.2 %)	28 (0.2 %)	17 (0.2 %)	19 (0.2 %)	13 (0.2 %)
Other	6738 (33.2 %)	3877 (33.3 %)	2706 (36.1 %)	2861 (33.0 %)	2050 (36.6 %)
Missing	2155 (10.6 %)	1357 (11.6 %)	892 (11.9 %)	798 (9.2 %)	483 (8.6 %)
**Ethnicity**					
Non-Hispanic	11,307 (55.7 %)	6303 (54.1 %)	3708 (49.5 %)	5004 (57.7 %)	3002 (53.6 %)
Hispanic	7123 (35.1 %)	4112 (35.3 %)	2970 (39.7 %)	3011 (34.7 %)	2191 (39.1 %)
Missing	1888 (9.3 %)	1237 (10.6 %)	809 (10.8 %)	651 (7.5 %)	407 (7.3 %)
**BMI**					
Underweight (<18.5)	919 (4.5 %)	569 (4.9 %)	329 (4.4 %)	350 (4.0 %)	248 (4.4 %)
Normal weight (≥18.5–24)	4718 (23.2 %)	2670 (22.9 %)	1750 (23.4 %)	2048 (23.6 %)	1336 (23.9 %)
Overweight (≥25–29)	6681 (32.9 %)	3784 (32.5 %)	2427 (32.4 %)	2897 (33.4 %)	1866 (33.3 %)
Obesity Class I (≥30–34)	4381 (21.6 %)	2527 (21.7 %)	1620 (21.6 %)	1854 (21.4 %)	1192 (21.3 %)
Obesity Class II (≥35–39)	2028 (10.0 %)	1198 (10.3 %)	775 (10.4 %)	830 (9.6 %)	517 (9.2 %)
Obesity Class III (40+)	1591 (7.8 %)	904 (7.8 %)	586 (7.8 %)	687 (7.9 %)	441 (7.9 %)
**Smoking status**					
Never smoked	17,976 (88.5 %)	10,290 (88.3 %)	6436 (86.0 %)	7686 (88.7 %)	4838 (86.4 %)
Ever smoked	2342 (11.5 %)	1362 (11.7 %)	1051 (14.0 %)	980 (11.3 %)	762 (13.6 %)
**Asthma**					
No	16,862 (83.0 %)	9702 (83.3 %)	6159 (82.3 %)	7160 (82.6 %)	4565 (81.5 %)
Yes	3456 (17.0 %)	1950 (16.7 %)	1328 (17.7 %)	1506 (17.4 %)	1035 (18.5 %)
**Diabetes**					
No	11,021 (54.2 %)	6124 (52.6 %)	3718 (49.7 %)	4897 (56.5 %)	2999 (53.6 %)
Yes	9297 (45.8 %)	5528 (47.4 %)	3769 (50.3 %)	3769 (43.5 %)	2601 (46.4 %)
**Hypertension**					
No	5821 (28.6 %)	3238 (27.8 %)	1854 (24.8 %)	2583 (29.8 %)	1481 (26.4 %)
Yes	14,497 (71.4 %)	8414 (72.2 %)	5633 (75.2 %)	6083 (70.2 %)	4119 (73.6 %)
**ARDS**					
No	17,497 (86.1 %)	9756 (83.7 %)	6287 (84.0 %)	7741 (89.3 %)	4948 (88.4 %)
Yes	2821 (13.9 %)	1896 (16.3 %)	1200 (16.0 %)	925 (10.7 %)	652 (11.6 %)
**Pneumonia**					
No	15,341 (75.5 %)	10,527 (90.3 %)	6997 (93.5 %)	4814 (55.6 %)	2923 (52.2 %)
Yes	4977 (24.5 %)	1125 (9.7 %)	490 (6.5 %)	3852 (44.4 %)	2677 (47.8 %)
**Dialysis**					
No	20,037 (98.6 %)	11,442 (98.2 %)	7380 (98.6 %)	8620 (98.2 %)	5560 (99.3 %)
Yes	281 (1.4 %)	210 (1.8 %)	107 (1.4 %)	71 (0.8 %)	40 (0.7 %)
**Ventilation**					
No	17,782 (87.5 %)	9814 (84.2 %)	6191 (82.7 %)	7968 (91.9 %)	5065 (90.4 %)
Yes	2536 (12.5 %)	1838 (15.8 %)	1296 (17.3 %)	698 (8.1 %)	535 (9.6 %)
**Deceased**					
No	16,866 (83.0 %)	9168 (78.7 %)	5702 (76.2 %)	7698 (88.8 %)	4865 (86.9 %)
Yes	3452 (17.0 %)	2484 (21.3 %)	1785 (23.8 %)	968 (11.2 %)	735 (13.1 %)
**Pollutants**					
PM_2.5_ (μg/m^3^)	9.0 (8.5–9.3)	8.9 (8.5–9.3)	9.1 (8.6–9.3)	9.0 (8.5–9.4)	9.1 (8.6–9.3)
BC (abs)	1.1 (0.9–1.2)	1.1 (0.9–1.2)	1.1 (1.0−1.1)	1.1 (0.9–1.2)	1.1 (1.0–1.2)
NO_2_ (ppb)	21.0 (19.0–23.2)	20.8 (19.0–23.0)	20.7 (18.6–22.1)	21.1 (19.0–23.2)	20.8 (19.0–23.0)
O_3_ (ppb)	30.4 (30.0–31.4)	30.4 (29.8–31.4)	30.4 (30.0–31.4)	30.4 (29.6–31.6)	30.4 (29.1–31.4)
**NEVI**					
Overall	3.7 (3.3–4.3)	3.7 (3.3–4.3)	3.8 (3.3–4.3)	3.7 (3.3–4.3)	3.7 (3.3–4.3)
Demographic	3.3 (2.8–3.7)	3.3 (2.8–3.7)	3.5 (2.9–3.7)	3.3 (2.7–3.7)	3.3 (2.9–3.7)
Economic	4.0 (3.5–5.0)	4.0 (3.5–5.0)	4.3 (3.5–4.8)	4.0 (3.4–5.0)	4.0 (3.5–5.0)
Residential	3.6 (3.2–3.9)	3.6 (3.2–3.9)	3.7 (3.2–4.2)	3.6 (3.2–3.9)	3.7 (3.2–4.2)
Health	4.1 (3.3–4.7)	4.1 (3.4–4.7)	4.2 (3.4–4.7)	4.1 (3.3–4.9)	4.1 (3.1–4.7)

1 INSIGHT harmonized data repository includes electronic health record data across metropolitan healthcare systems in the NYC metropolitan area.

2 Dialysis outcome is defined as first-time dialysis use after COVID-19 diagnosis.

3 Categorical variables reported in the count (percentage) format; age reported in the mean (standard deviation) format; ambient air pollutants and neighborhood environmental vulnerability index (NEVI) scores are presented using median and inter-quartile range.

4 Abbreviations: NEVI – Neighborhood Environmental Vulnerability Index; BMI – body mass index; PM_2.5_ – fine particulate matter; BC – black carbon; NO_2_ – nitrogen dioxide; O_3_ – ozone; μg/m^3^ – micrograms per cubic meter; abs – absorbance units; ppb – parts per billion.

## Data Availability

The data that has been used is confidential.
