# Supplementary material for: The relationship between chronic air pollution exposure, neighborhood environmental vulnerability, and adverse COVID-19 morbidities among hospitalized New York City residents
Source: Environ Int. Author manuscript; Available in PMC 2025 Sep 27. (PMC12476271; doi:10.1016/j.envint.2025.109660)
Supplement: 1 [file NIHMS2105992-supplement-1.docx]

**SUPPLEMENT**

**The Relationship between Chronic Air Pollution Exposure, Neighborhood Environmental Vulnerability, and Adverse COVID-19 Morbidities among Hospitalized New York City Residents**

**Supplement Tables**

**Supplement Table 1:** Features used to construct the Neighborhood Environmental Vulnerability Index……………..…...….…2

**Supplement Table 2a-d:** Analytical results when using categorical measures for air pollutants……………..…………..……..….…4

**Supplement Table 3a-d:** Analytical results when adjusting for individual domains of NEVI….........................................…...8

**Supplement Table 4:** Dispersion statistics for Poisson regression models regarding the relationship between chronic air pollutant exposure and ARDS, dialysis, pneumonia, and ventilation……..…………………………………………….………………….………12

**Supplement Table 5:** Effect estimates for the relationship between chronic air pollutant exposure and length of hospital stay, stratified by hospital phase and NEVI tertile……………………………………………………………………………….………………….………13

**Supplement Table 6:** Effect estimates for the relationship between chronic air pollutant exposure and ARDS risk, stratified by hospital phase and NEVI tertile…………………………………………………………………………………………………..……….……..14

**Supplement Table 7:** Effect estimates for the relationship between chronic air pollutant exposure and pneumonia risk, stratified by hospital phase and NEVI tertile………………………………………………….………………………………………..……………………..15

**Supplement Table 8:** Effect estimates for the relationship between chronic air pollutant exposure and ventilation risk, stratified by hospital phase and NEVI tertile……………………………………………………………………….……..…...................................16

**Supplement Table 9:** Effect estimates for the relationship between chronic air pollutant exposure and dialysis risk, stratified by hospital phase and NEVI tertile…………………………………………………..…………………......………………………………………17

**Supplement Table 10**: Effect estimates for two-pollutant analyses for the relationships between chronic PM_2.5_, BC, and NO_2_ exposure and adverse COVID-19 morbidities, when adjusting for O_3_………………………………………..……………..................18

**Supplement Table 11:** Effect estimates for the relationship between chronic air pollution exposure and COVID-19 pneumonia risk, within racial and ethnic subpopulations………………………………………..……………...........................................19

**Supplement Table 12**: RERI analyses between chronic air pollution exposure and neighborhood environmental vulnerability on adverse COVID-19 morbidities………………………………………………………………………………..……………..................20

**Supplement Table 13:** Effect estimates for the relationship between chronic air pollutant exposure and length of hospital stay, stratified by testing rate…………………………………………………………………………………..……………..…..…...................21

**Supplement Figures**

**Supplement Figure 1:** Spearman rank correlations between chronic air pollutant exposure and NEVI …………………..……..22

**Supplement Figure 2**: Sensitivity analyses for selection bias………………………………….………………………………………………….……23

**Supplement Table 1:** Features that were used to construct the Neighborhood Environmental Vulnerability Index, organized by domain and subdomain^1-3^

| **Domain** | **Subdomain (Number of Features,**  **Weight of Overall Index)** | **Features** |
| --- | --- | --- |
| **Demographics**  (7 subdomains,  12 features) | Age  (2 features, 1/28) | • Age under 18 (%) • Age 65 or older (%) |
|  | Female-led household  (1 feature, 1/28) | • Female-led households (%) |
|  | Immigration  (4 features, 1/28) | • Speak English "not at all" or "not well" (%) • Foreign-born (%) • Entered U.S. 2010 or later (%) • Not a U.S. citizen (%) |
|  | Disability  (1 feature, 1/28) | • With a disability (%) |
|  | Single Parent Households  (1 feature, 1/28) | • Children (age younger than 18) living with one parent (%) |
|  | Mobility  (2 features, 1/28) | • Walk, bicycle, or take public transportation, taxicab, other non-automobile means (%) • Aggregate travel time to work |
|  | Social Isolation  (1 feature, 1/28) | • Live alone (%) |
| **Economic Indicators** (6 subdomains,  7 features) | Income and poverty  (2 features, 1/24) | • Household Income (median) • Income in past 12 months below poverty level (%) |
|  | Occupation  (1 feature, 1/24) | • Work in service, natural resources, construction, maintenance, production, transportation, or material moving occupations (%) |
|  | Income inequality  (1 feature, 1/24) | • Gini Index of income inequality |
|  | Unemployment  (1 feature, 1/24) | • Unemployed among those in labor force aged 20-64 (%) |
|  | Education  (1 feature, 1/24) | • With less than high school education (%) |
|  | Vehicle availability  (1 feature, 1/24) | • % with no vehicle available (%) |
| **Residential Characteristics and Density** (7 subdomains,  8 features) | Population Density  (1 feature, 1/28) | • Population per square mile (%) |
|  | Group Quarters  (1 feature, 1/28) | • Live in group quarters (%) |
|  | Occupants per room  (1 feature, 1/28) | • More than one occupant per room (%) |
|  | Age of housing structure  (1 feature, 1/28) | • Age of housing in 2019 (years) |
|  | Units in housing structure  (2 features, 1/28) | • Living in structure with 1 attached housing unit, 2 or more units, or in a mobile home, boat, RV, or van (%)  • Living in structure with 20 or more units (%) |
|  | Changing residence  (1 feature, 1/28) | • Moved in the past year (%) |
|  | Housing vacancy  (1 feature, 1/28) | • Vacancy (%) |
| **Health Behaviors, Outcomes, Prevention Practices, and Access ^b^**  (4 subdomains,  27 features) | Unhealthy behaviors  (4 features, 1/16) | • Currently smoke • Binge drink • Have no leisure-time physical activity • Sleep for less than 7 hours per day |
|  | Health outcomes  (14 features, 1/16) | • High blood pressure, 2017 • High blood pressure medication use, among those with high blood pressure, 2017 • Obesity • Cancer, non-skin • Current asthma • Coronary heart disease • Stroke • COPD • Diabetes • High cholesterol, 2017 • Chronic Kidney Disease • Mental health not good for >=14 days • Physical health not good for >=14 days • All teeth lost |
|  | Prevention practices  (8 features, 1/16) | • Routine checkup within past year • Adult men aged 65 or older who are on date with clinical preventive services (flu shot past year, PPV shot ever, colorectal cancer screening) • Adult women aged 65 or older who are on date with clinical preventive services (flu shot past year, PPV shot ever, colorectal cancer screening, mammogram within past 2 years) • Visit to dentist or dental clinic • Cervical cancer screening among women aged 21-65 • Cholesterol screening, 2017 • Colonoscopy screening among adults aged 50-75 years • Mammography use among women aged 50-74 years |
|  | Health insurance access  (1 feature, 1/16) | • Lack of health insurance |

^1^ Data sources: U.S. Census American Community Survey (ACS) 2015-2019 5-year estimates, Centers for Disease Control and Prevention (CDC) 2020 data release from the PLACES Project, NEVI repository

^2^ Unless noted, crude prevalence estimates were used from models among adults aged 18 or older in 2018

^3^ Reference: Azan A., Kannoth S., Zhang C., Shafiq M., Chambers E.C., Sheffield P.E., et al. 2025. Neighborhood environmental vulnerability factors strongly drove COVID-19 fatality and excess all-cause mortality in New York City, while long-term air pollutant associations were weak and varied. Sci. Total Environ. 989, 179874.

| **Supplement Table 2a:** Risk ratio estimates for adverse COVID-19 for chronic air pollution (2009-2019) by quartiles (Phase 1: 3/2020 – 6/2020) | | | | | | | | | | | | | | | | | | | | | | | |
| --- | --- | --- | --- | --- | --- | --- | --- | --- | --- | --- | --- | --- | --- | --- | --- | --- | --- | --- | --- | --- | --- | --- | --- |
| **Outcome** | | **Pollutant** | | **RR** | | **95% CI** | | **Outcome** | | **Pollutant** | | **RR** | | **95% CI** | | **Outcome** | | **Pollutant** | | **RR** | | **95% CI** | |
| ARDS | | BC-Q1 | | 1 | |  | | Length of Stay | | BC-Q1 | | 1 | |  | | Ventilation | | BC-Q1 | | 1 | |  | |
| ARDS | | BC-Q2 | | 0.93 | | 0.80,1.08 | | Length of Stay | | BC-Q2 | | 0.98 | | 0.90,1.06 | | Ventilation | | BC-Q2 | | 0.93 | | 0.78,1.10 | |
| ARDS | | BC-Q3 | | 0.75 | | 0.66,0.86 | | Length of Stay | | BC-Q3 | | 0.96 | | 0.89,1.04 | | Ventilation | | BC-Q3 | | 1.06 | | 0.91,1.24 | |
| ARDS | | BC-Q4 | | 0.84 | | 0.72,0.98 | | Length of Stay | | BC-Q4 | | 0.96 | | 0.89,1.04 | | Ventilation | | BC-Q4 | | 0.90 | | 0.75,1.06 | |
| ARDS | | NO_2_-Q1 | | 1 | |  | | Length of Stay | | NO_2_-Q1 | | 1 | |  | | Ventilation | | NO_2_-Q1 | | 1 | |  | |
| ARDS | | NO_2_-Q2 | | 1.11 | | 0.98,1.25 | | Length of Stay | | NO_2_-Q2 | | 0.97 | | 0.92,1.03 | | Ventilation | | NO_2_-Q2 | | 1.04 | | 0.93,1.17 | |
| ARDS | | NO_2_-Q3 | | 1.35 | | 1.18,1.54 | | Length of Stay | | NO_2_-Q3 | | 1.01 | | 0.95,1.08 | | Ventilation | | NO_2_-Q3 | | 1.03 | | 0.90,1.17 | |
| ARDS | | NO_2_-Q4 | | 1.17 | | 1.03,1.34 | | Length of Stay | | NO_2_-Q4 | | 1.02 | | 0.95,1.09 | | Ventilation | | NO_2_-Q4 | | 0.89 | | 0.77,1.02 | |
| ARDS | | PM_2.5_-Q1 | | 1 | |  | | Length of Stay | | PM_2.5_-Q1 | | 1 | |  | | Ventilation | | PM_2.5_-Q1 | | 1 | |  | |
| ARDS | | PM_2.5_-Q2 | | 0.91 | | 0.78,1.07 | | Length of Stay | | PM_2.5_-Q2 | | 0.99 | | 0.91,1.08 | | Ventilation | | PM_2.5_-Q2 | | 1.08 | | 0.91,1.28 | |
| ARDS | | PM_2.5_-Q3 | | 0.83 | | 0.70,0.97 | | Length of Stay | | PM_2.5_-Q3 | | 0.98 | | 0.90,1.07 | | Ventilation | | PM_2.5_-Q3 | | 1.10 | | 0.92,1.32 | |
| ARDS | | PM_2.5_-Q4 | | 0.89 | | 0.75,1.06 | | Length of Stay | | PM_2.5_-Q4 | | 1.03 | | 0.94,1.13 | | Ventilation | | PM_2.5_-Q4 | | 0.91 | | 0.75,1.11 | |
| ARDS | | O_3_-Q1 | | 1 | |  | | Length of Stay | | O_3_-Q1 | | 1 | |  | | Ventilation | | O_3_-Q1 | | 1 | |  | |
| ARDS | | O_3_-Q2 | | 1.05 | | 0.94,1.18 | | Length of Stay | | O_3_-Q2 | | 1.00 | | 0.94,1.05 | | Ventilation | | O_3_-Q2 | | 1.21 | | 1.07,1.37 | |
| ARDS | | O_3_-Q3 | | 0.96 | | 0.85,1.08 | | Length of Stay | | O_3_-Q3 | | 0.94 | | 0.89,1.00 | | Ventilation | | O_3_-Q3 | | 1.24 | | 1.09,1.42 | |
| ARDS | | O_3_-Q4 | | 0.97 | | 0.84,1.13 | | Length of Stay | | O_3_-Q4 | | 1.03 | | 0.96,1.12 | | Ventilation | | O_3_-Q4 | | 1.16 | | 0.99,1.36 | |
| Dialysis | | BC-Q1 | | 1 | |  | | Pneumonia | | BC-Q1 | | 1 | |  | |  | |  | |  | |  | |
| Dialysis | | BC-Q2 | | 0.91 | | 0.61,1.35 | | Pneumonia | | BC-Q2 | | 0.77 | | 0.66,0.91 | |  | |  | |  | |  | |
| Dialysis | | BC-Q3 | | 0.28 | | 0.18,0.44 | | Pneumonia | | BC-Q3 | | 0.30 | | 0.25,0.36 | |  | |  | |  | |  | |
| Dialysis | | BC-Q4 | | 0.62 | | 0.39,0.99 | | Pneumonia | | BC-Q4 | | 0.74 | | 0.63,0.87 | |  | |  | |  | |  | |
| Dialysis | | NO_2_-Q1 | | 1 | |  | | Pneumonia | | NO_2_-Q1 | | 1 | |  | |  | |  | |  | |  | |
| Dialysis | | NO_2_-Q2 | | 1.02 | | 0.65,1.58 | | Pneumonia | | NO_2_-Q2 | | 0.90 | | 0.76,1.07 | |  | |  | |  | |  | |
| Dialysis | | NO_2_-Q3 | | 2.81 | | 1.78,4.44 | | Pneumonia | | NO_2_-Q3 | | 1.87 | | 1.57,2.23 | |  | |  | |  | |  | |
| Dialysis | | NO_2_-Q4 | | 1.94 | | 1.25,2.99 | | Pneumonia | | NO_2_-Q4 | | 1.65 | | 1.41,1.94 | |  | |  | |  | |  | |
| Dialysis | | PM_2.5_-Q1 | | 1 | |  | | Pneumonia | | PM_2.5_-Q1 | | 1 | |  | |  | |  | |  | |  | |
| Dialysis | | PM_2.5_-Q2 | | 0.74 | | 0.47,1.16 | | Pneumonia | | PM_2.5_-Q2 | | 0.52 | | 0.44,0.62 | |  | |  | |  | |  | |
| Dialysis | | PM_2.5_-Q3 | | 0.51 | | 0.30,0.85 | | Pneumonia | | PM_2.5_-Q3 | | 0.44 | | 0.37,0.53 | |  | |  | |  | |  | |
| Dialysis | | PM_2.5_-Q4 | | 0.79 | | 0.47,1.33 | | Pneumonia | | PM_2.5_-Q4 | | 0.70 | | 0.58,0.83 | |  | |  | |  | |  | |
| Dialysis | | O_3_-Q1 | | 1 | |  | | Pneumonia | | O_3_-Q1 | | 1 | |  | |  | |  | |  | |  | |
| Dialysis | | O_3_-Q2 | | 0.72 | | 0.51,1.02 | | Pneumonia | | O_3_-Q2 | | 0.71 | | 0.61,0.82 | |  | |  | |  | |  | |
| Dialysis | | O_3_-Q3 | | 0.47 | | 0.32,0.70 | | Pneumonia | | O_3_-Q3 | | 0.56 | | 0.47,0.65 | |  | |  | |  | |  | |
| Dialysis | | O_3_-Q4 | | 0.61 | | 0.39,0.96 | | Pneumonia | | O_3_-Q4 | | 0.83 | | 0.70,0.97 | |  | |  | |  | |  | |
| **Supplement Table 2b:**  Risk ratio estimates for adverse COVID-19 for chronic air pollution (2009-2019) by quartiles (Phases 2 & 3: 7/2020 – 2/2021) | | | | | | | | | | | | | | | | | | | | | | | |
| **Outcome** | | **Pollutant** | | **RR** | | **95% CI** | | **Outcome** | | **Pollutant** | | **RR** | | **95% CI** | | **Outcome** | | **Pollutant** | | **RR** | | **95% CI** | |
| ARDS | | BC-Q1 | | 1 | |  | | Length of Stay | | BC-Q1 | | 1 | |  | | Ventilation | | BC-Q1 | | 1 | |  | |
| ARDS | | BC-Q2 | | 0.91 | | 0.73,1.13 | | Length of Stay | | BC-Q2 | | 0.96 | | 0.88,1.04 | | Ventilation | | BC-Q2 | | 0.96 | | 0.73,1.28 | |
| ARDS | | BC-Q3 | | 0.92 | | 0.76,1.11 | | Length of Stay | | BC-Q3 | | 0.89 | | 0.83,0.96 | | Ventilation | | BC-Q3 | | 1.22 | | 0.96,1.56 | |
| ARDS | | BC-Q4 | | 0.71 | | 0.57,0.89 | | Length of Stay | | BC-Q4 | | 0.99 | | 0.92,1.08 | | Ventilation | | BC-Q4 | | 0.64 | | 0.48,0.84 | |
| ARDS | | NO_2_-Q1 | | 1 | |  | | Length of Stay | | NO_2_-Q1 | | 1 | |  | | Ventilation | | NO_2_-Q1 | | 1 | |  | |
| ARDS | | NO_2_-Q2 | | 0.92 | | 0.78,1.08 | | Length of Stay | | NO_2_-Q2 | | 0.99 | | 0.92,1.06 | | Ventilation | | NO_2_-Q2 | | 1.03 | | 0.85,1.24 | |
| ARDS | | NO_2_-Q3 | | 0.81 | | 0.67,0.98 | | Length of Stay | | NO_2_-Q3 | | 1.10 | | 1.02,1.18 | | Ventilation | | NO_2_-Q3 | | 0.76 | | 0.60,0.95 | |
| ARDS | | NO_2_-Q4 | | 0.70 | | 0.58,0.85 | | Length of Stay | | NO_2_-Q4 | | 1.18 | | 1.10,1.26 | | Ventilation | | NO_2_-Q4 | | 0.46 | | 0.36,0.59 | |
| ARDS | | PM_2.5_-Q1 | | 1 | |  | | Length of Stay | | PM_2.5_-Q1 | | 1 | |  | | Ventilation | | PM_2.5_-Q1 | | 1 | |  | |
| ARDS | | PM_2.5_-Q2 | | 0.99 | | 0.80,1.21 | | Length of Stay | | PM_2.5_-Q2 | | 0.92 | | 0.85,1.00 | | Ventilation | | PM_2.5_-Q2 | | 1.28 | | 0.98,1.67 | |
| ARDS | | PM_2.5_-Q3 | | 0.87 | | 0.70,1.08 | | Length of Stay | | PM_2.5_-Q3 | | 0.94 | | 0.86,1.02 | | Ventilation | | PM_2.5_-Q3 | | 1.09 | | 0.82,1.46 | |
| ARDS | | PM_2.5_-Q4 | | 0.69 | | 0.54,0.87 | | Length of Stay | | PM_2.5_-Q4 | | 1.03 | | 0.95,1.13 | | Ventilation | | PM_2.5_-Q4 | | 0.57 | | 0.41,0.79 | |
| ARDS | | O_3_-Q1 | | 1 | |  | | Length of Stay | | O_3_-Q1 | | 1 | |  | | Ventilation | | O_3_-Q1 | | 1 | |  | |
| ARDS | | O_3_-Q2 | | 1.20 | | 1.01,1.44 | | Length of Stay | | O_3_-Q2 | | 0.89 | | 0.84,0.95 | | Ventilation | | O_3_-Q2 | | 1.76 | | 1.39,2.22 | |
| ARDS | | O_3_-Q3 | | 1.20 | | 0.99,1.45 | | Length of Stay | | O_3_-Q3 | | 0.86 | | 0.81,0.92 | | Ventilation | | O_3_-Q3 | | 1.95 | | 1.53,2.49 | |
| ARDS | | O_3_-Q4 | | 1.30 | | 1.06,1.59 | | Length of Stay | | O_3_-Q4 | | 0.93 | | 0.86,1.00 | | Ventilation | | O_3_-Q4 | | 1.84 | | 1.41,2.40 | |
| Dialysis | | BC-Q1 | | 1 | |  | | Pneumonia | | BC-Q1 | | 1 | |  | |  | |  | |  | |  | |
| Dialysis | | BC-Q2 | | 0.55 | | 0.26,1.14 | | Pneumonia | | BC-Q2 | | 0.83 | | 0.76,0.90 | |  | |  | |  | |  | |
| Dialysis | | BC-Q3 | | 0.27 | | 0.13,0.57 | | Pneumonia | | BC-Q3 | | 0.89 | | 0.83,0.95 | |  | |  | |  | |  | |
| Dialysis | | BC-Q4 | | 0.48 | | 0.24,0.97 | | Pneumonia | | BC-Q4 | | 0.62 | | 0.58,0.68 | |  | |  | |  | |  | |
| Dialysis | | NO_2_-Q1 | | 1 | |  | | Pneumonia | | NO_2_-Q1 | | 1 | |  | |  | |  | |  | |  | |
| Dialysis | | NO_2_-Q2 | | 0.46 | | 0.22,0.98 | | Pneumonia | | NO_2_-Q2 | | 0.95 | | 0.90,1.01 | |  | |  | |  | |  | |
| Dialysis | | NO_2_-Q3 | | 1.26 | | 0.60,2.66 | | Pneumonia | | NO_2_-Q3 | | 0.77 | | 0.72,0.83 | |  | |  | |  | |  | |
| Dialysis | | NO_2_-Q4 | | 1.19 | | 0.63,2.26 | | Pneumonia | | NO_2_-Q4 | | 0.58 | | 0.54,0.63 | |  | |  | |  | |  | |
| Dialysis | | PM_2.5_-Q1 | | 1 | |  | | Pneumonia | | PM_2.5_-Q1 | | 1 | |  | |  | |  | |  | |  | |
| Dialysis | | PM_2.5_-Q2 | | 0.47 | | 0.23,0.97 | | Pneumonia | | PM_2.5_-Q2 | | 0.89 | | 0.83,0.96 | |  | |  | |  | |  | |
| Dialysis | | PM_2.5_-Q3 | | 0.44 | | 0.20,0.95 | | Pneumonia | | PM_2.5_-Q3 | | 0.81 | | 0.75,0.88 | |  | |  | |  | |  | |
| Dialysis | | PM_2.5_-Q4 | | 0.58 | | 0.27,1.26 | | Pneumonia | | PM_2.5_-Q4 | | 0.58 | | 0.53,0.63 | |  | |  | |  | |  | |
| Dialysis | | O_3_-Q1 | | 1 | |  | | Pneumonia | | O_3_-Q1 | | 1 | |  | |  | |  | |  | |  | |
| Dialysis | | O_3_-Q2 | | 0.63 | | 0.34,1.16 | | Pneumonia | | O_3_-Q2 | | 1.39 | | 1.29,1.50 | |  | |  | |  | |  | |
| Dialysis | | O_3_-Q3 | | 0.34 | | 0.16,0.74 | | Pneumonia | | O_3_-Q3 | | 1.53 | | 1.41,1.65 | |  | |  | |  | |  | |
| Dialysis | | O_3_-Q4 | | 0.79 | | 0.41,1.54 | | Pneumonia | | O_3_-Q4 | | 1.59 | | 1.47,1.73 | |  | |  | |  | |  | |
| **Supplement Table 2c:**  Risk ratio estimates for adverse COVID-19 for chronic air pollution (2009-2019) by quartiles (Phase 1 – 40% Subset: 3/2020 – 6/2020) | | | | | | | | | | | | | | | | | | | | | | | |
| **Outcome** | | **Pollutant** | | **RR** | | **95% CI** | | **Outcome** | | **Pollutant** | | **RR** | | **95% CI** | | **Outcome** | | **Pollutant** | | **RR** | | **95% CI** | |
| ARDS | | BC-Q1 | | 1 | |  | | Length of Stay | | BC-Q1 | | 1 | |  | | Ventilation | | BC-Q1 | | 1 | |  | |
| ARDS | | BC-Q2 | | 0.59 | | 0.41,0.84 | | Length of Stay | | BC-Q2 | | 1.16 | | 0.89,1.53 | | Ventilation | | BC-Q2 | | 0.64 | | 0.44,0.94 | |
| ARDS | | BC-Q3 | | 0.55 | | 0.40,0.77 | | Length of Stay | | BC-Q3 | | 1.10 | | 0.85,1.44 | | Ventilation | | BC-Q3 | | 0.70 | | 0.49,1.00 | |
| ARDS | | BC-Q4 | | 0.71 | | 0.51,1.00 | | Length of Stay | | BC-Q4 | | 1.03 | | 0.79,1.34 | | Ventilation | | BC-Q4 | | 0.64 | | 0.44,0.92 | |
| ARDS | | NO_2_-Q1 | | 1 | |  | | Length of Stay | | NO_2_-Q1 | | 1 | |  | | Ventilation | | NO_2_-Q1 | | 1 | |  | |
| ARDS | | NO_2_-Q2 | | 1.25 | | 1.06,1.47 | | Length of Stay | | NO_2_-Q2 | | 1.01 | | 0.94,1.09 | | Ventilation | | NO_2_-Q2 | | 1.05 | | 0.91,1.20 | |
| ARDS | | NO_2_-Q3 | | 1.63 | | 1.35,1.97 | | Length of Stay | | NO_2_-Q3 | | 1.08 | | 0.99,1.19 | | Ventilation | | NO_2_-Q3 | | 1.12 | | 0.95,1.31 | |
| ARDS | | NO_2_-Q4 | | 1.57 | | 1.32,1.87 | | Length of Stay | | NO_2_-Q4 | | 0.96 | | 0.88,1.04 | | Ventilation | | NO_2_-Q4 | | 0.98 | | 0.83,1.16 | |
| ARDS | | PM_2.5_-Q1 | | 1 | |  | | Length of Stay | | PM_2.5_-Q1 | | 1 | |  | | Ventilation | | PM_2.5_-Q1 | | 1 | |  | |
| ARDS | | PM_2.5_-Q2 | | 1.45 | | 0.21,9.87 | | Length of Stay | | PM_2.5_-Q2 | | 0.79 | | 0.39,1.58 | | Ventilation | | PM_2.5_-Q2 | | 0.84 | | 0.26,2.71 | |
| ARDS | | PM_2.5_-Q3 | | 1.45 | | 0.21,9.91 | | Length of Stay | | PM_2.5_-Q3 | | 0.76 | | 0.38,1.54 | | Ventilation | | PM_2.5_-Q3 | | 0.84 | | 0.26,2.72 | |
| ARDS | | PM_2.5_-Q4 | | 1.91 | | 0.28,13.0 | | Length of Stay | | PM_2.5_-Q4 | | 0.78 | | 0.39,1.57 | | Ventilation | | PM_2.5_-Q4 | | 0.73 | | 0.23,2.37 | |
| ARDS | | O_3_-Q1 | | 1 | |  | | Length of Stay | | O_3_-Q1 | | 1 | |  | | Ventilation | | O_3_-Q1 | | 1 | |  | |
| ARDS | | O_3_-Q2 | | 0.95 | | 0.83,1.09 | | Length of Stay | | O_3_-Q2 | | 1.07 | | 1.00,1.14 | | Ventilation | | O_3_-Q2 | | 1.19 | | 1.03,1.38 | |
| ARDS | | O_3_-Q3 | | 0.78 | | 0.67,0.91 | | Length of Stay | | O_3_-Q3 | | 1.03 | | 0.96,1.11 | | Ventilation | | O_3_-Q3 | | 1.23 | | 1.05,1.43 | |
| ARDS | | O_3_-Q4 | | 0.68 | | 0.53,0.87 | | Length of Stay | | O_3_-Q4 | | 1.08 | | 0.96,1.21 | | Ventilation | | O_3_-Q4 | | 1.20 | | 0.96,1.49 | |
| Dialysis | | BC-Q1 | | 1 | |  | | Pneumonia | | BC-Q1 | | 1 | |  | |  | |  | |  | |  | |
| Dialysis | | BC-Q2 | | 0.20 | | 0.09,0.47 | | Pneumonia | | BC-Q2 | | 0.45 | | 0.26,0.78 | |  | |  | |  | |  | |
| Dialysis | | BC-Q3 | | 0.09 | | 0.04,0.19 | | Pneumonia | | BC-Q3 | | 0.27 | | 0.16,0.45 | |  | |  | |  | |  | |
| Dialysis | | BC-Q4 | | 0.32 | | 0.15,0.67 | | Pneumonia | | BC-Q4 | | 0.87 | | 0.53,1.43 | |  | |  | |  | |  | |
| Dialysis | | NO_2_-Q1 | | 1 | |  | | Pneumonia | | NO_2_-Q1 | | 1 | |  | |  | |  | |  | |  | |
| Dialysis | | NO_2_-Q2 | | 4.49 | | 1.36,14.8 | | Pneumonia | | NO_2_-Q2 | | 2.40 | | 1.53,3.78 | |  | |  | |  | |  | |
| Dialysis | | NO_2_-Q3 | | 15.1 | | 4.25,53.6 | | Pneumonia | | NO_2_-Q3 | | 7.47 | | 4.69,11.9 | |  | |  | |  | |  | |
| Dialysis | | NO_2_-Q4 | | 14.8 | | 4.61,47.6 | | Pneumonia | | NO_2_-Q4 | | 7.94 | | 5.14,12.3 | |  | |  | |  | |  | |
| Dialysis | | PM_2.5_-Q1 | | 1 | |  | | Pneumonia | | PM_2.5_-Q1 | | 1 | |  | |  | |  | |  | |  | |
| Dialysis | | PM_2.5_-Q2 | | – | | – | | Pneumonia | | PM_2.5_-Q2 | | – | | – | |  | |  | |  | |  | |
| Dialysis | | PM_2.5_-Q3 | | – | | – | | Pneumonia | | PM_2.5_-Q3 | | – | | – | |  | |  | |  | |  | |
| Dialysis | | PM_2.5_-Q4 | | – | | – | | Pneumonia | | PM_2.5_-Q4 | | – | | – | |  | |  | |  | |  | |
| Dialysis | | O_3_-Q1 | | 1 | |  | | Pneumonia | | O_3_-Q1 | | 1 | |  | |  | |  | |  | |  | |
| Dialysis | | O_3_-Q2 | | 0.61 | | 0.40,0.91 | | Pneumonia | | O_3_-Q2 | | 0.57 | | 0.47,0.69 | |  | |  | |  | |  | |
| Dialysis | | O_3_-Q3 | | 0.17 | | 0.09,0.31 | | Pneumonia | | O_3_-Q3 | | 0.15 | | 0.11,0.20 | |  | |  | |  | |  | |
| Dialysis | | O_3_-Q4 | | 0.06 | | 0.01,0.41 | | Pneumonia | | O_3_-Q4 | | 0.08 | | 0.04,0.19 | |  | |  | |  | |  | |
| **Supplement Table 2d:**  Risk ratio estimates for adverse COVID-19 for chronic air pollution (2009-2019) by quartiles (Phases 2 & 3 – 40% Subset: 7/2020 – 2/2021) | | | | | | | | | | | | | | | | | | | | | | | |
| **Outcome** | | **Pollutant** | | **RR** | | **95% CI** | | **Outcome** | | **Pollutant** | | **RR** | | **95% CI** | | **Outcome** | | **Pollutant** | | **RR** | | **95% CI** | |
| ARDS | | BC-Q1 | | 1 | |  | | Length of Stay | | BC-Q1 | | 1 | |  | | Ventilation | | BC-Q1 | | 1 | |  | |
| ARDS | | BC-Q2 | | 0.95 | | 0.66,1.38 | | Length of Stay | | BC-Q2 | | 0.93 | | 0.79,1.09 | | Ventilation | | BC-Q2 | | 0.96 | | 0.62,1.47 | |
| ARDS | | BC-Q3 | | 0.86 | | 0.62,1.18 | | Length of Stay | | BC-Q3 | | 0.84 | | 0.73,0.96 | | Ventilation | | BC-Q3 | | 0.97 | | 0.66,1.41 | |
| ARDS | | BC-Q4 | | 0.74 | | 0.53,1.04 | | Length of Stay | | BC-Q4 | | 0.94 | | 0.82,1.09 | | Ventilation | | BC-Q4 | | 0.46 | | 0.30,0.70 | |
| ARDS | | NO_2_-Q1 | | 1 | |  | | Length of Stay | | NO_2_-Q1 | | 1 | |  | | Ventilation | | NO_2_-Q1 | | 1 | |  | |
| ARDS | | NO_2_-Q2 | | 0.87 | | 0.72,1.06 | | Length of Stay | | NO_2_-Q2 | | 1.04 | | 0.96,1.13 | | Ventilation | | NO_2_-Q2 | | 0.95 | | 0.77,1.16 | |
| ARDS | | NO_2_-Q3 | | 0.89 | | 0.70,1.13 | | Length of Stay | | NO_2_-Q3 | | 1.15 | | 1.04,1.27 | | Ventilation | | NO_2_-Q3 | | 0.77 | | 0.60,1.00 | |
| ARDS | | NO_2_-Q4 | | 0.77 | | 0.62,0.96 | | Length of Stay | | NO_2_-Q4 | | 1.16 | | 1.06,1.27 | | Ventilation | | NO_2_-Q4 | | 0.43 | | 0.32,0.58 | |
| ARDS | | PM_2.5_-Q1 | | 1 | |  | | Length of Stay | | PM_2.5_-Q1 | | 1 | |  | | Ventilation | | PM_2.5_-Q1 | | 1 | |  | |
| ARDS | | PM_2.5_-Q2 | | 1.00 | | 0.68,1.47 | | Length of Stay | | PM_2.5_-Q2 | | 0.89 | | 0.75,1.05 | | Ventilation | | PM_2.5_-Q2 | | 1.07 | | 0.69,1.64 | |
| ARDS | | PM_2.5_-Q3 | | 0.80 | | 0.54,1.17 | | Length of Stay | | PM_2.5_-Q3 | | 0.91 | | 0.77,1.07 | | Ventilation | | PM_2.5_-Q3 | | 0.79 | | 0.51,1.22 | |
| ARDS | | PM_2.5_-Q4 | | 0.77 | | 0.51,1.16 | | Length of Stay | | PM_2.5_-Q4 | | 1.00 | | 0.84,1.18 | | Ventilation | | PM_2.5_-Q4 | | 0.35 | | 0.21,0.58 | |
| ARDS | | O_3_-Q1 | | 1 | |  | | Length of Stay | | O_3_-Q1 | | 1 | |  | | Ventilation | | O_3_-Q1 | | 1 | |  | |
| ARDS | | O_3_-Q2 | | 1.25 | | 1.03,1.53 | | Length of Stay | | O_3_-Q2 | | 0.92 | | 0.86,1.00 | | Ventilation | | O_3_-Q2 | | 1.83 | | 1.40,2.40 | |
| ARDS | | O_3_-Q3 | | 1.08 | | 0.86,1.35 | | Length of Stay | | O_3_-Q3 | | 0.88 | | 0.82,0.96 | | Ventilation | | O_3_-Q3 | | 2.07 | | 1.58,2.72 | |
| ARDS | | O_3_-Q4 | | 1.31 | | 1.01,1.68 | | Length of Stay | | O_3_-Q4 | | 0.92 | | 0.83,1.02 | | Ventilation | | O_3_-Q4 | | 2.33 | | 1.72,3.15 | |
| Dialysis | | BC-Q1 | | 1 | |  | | Pneumonia | | BC-Q1 | | 1 | |  | |  | |  | |  | |  | |
| Dialysis | | BC-Q2 | | 0.25 | | 0.04,1.39 | | Pneumonia | | BC-Q2 | | 0.85 | | 0.76,0.96 | |  | |  | |  | |  | |
| Dialysis | | BC-Q3 | | 0.24 | | 0.07,0.83 | | Pneumonia | | BC-Q3 | | 0.82 | | 0.74,0.91 | |  | |  | |  | |  | |
| Dialysis | | BC-Q4 | | 0.72 | | 0.23,2.29 | | Pneumonia | | BC-Q4 | | 0.54 | | 0.48,0.61 | |  | |  | |  | |  | |
| Dialysis | | NO_2_-Q1 | | 1 | |  | | Pneumonia | | NO_2_-Q1 | | 1 | |  | |  | |  | |  | |  | |
| Dialysis | | NO_2_-Q2 | | 0.42 | | 0.13,1.37 | | Pneumonia | | NO_2_-Q2 | | 0.97 | | 0.91,1.04 | |  | |  | |  | |  | |
| Dialysis | | NO_2_-Q3 | | 1.71 | | 0.56,5.24 | | Pneumonia | | NO_2_-Q3 | | 0.86 | | 0.79,0.94 | |  | |  | |  | |  | |
| Dialysis | | NO_2_-Q4 | | 2.19 | | 0.87,5.48 | | Pneumonia | | NO_2_-Q4 | | 0.61 | | 0.55,0.67 | |  | |  | |  | |  | |
| Dialysis | | PM_2.5_-Q1 | | 1 | |  | | Pneumonia | | PM_2.5_-Q1 | | 1 | |  | |  | |  | |  | |  | |
| Dialysis | | PM_2.5_-Q2 | | 0.17 | | 0.04,0.80 | | Pneumonia | | PM_2.5_-Q2 | | 0.90 | | 0.80,1.02 | |  | |  | |  | |  | |
| Dialysis | | PM_2.5_-Q3 | | 0.29 | | 0.07,1.12 | | Pneumonia | | PM_2.5_-Q3 | | 0.82 | | 0.73,0.93 | |  | |  | |  | |  | |
| Dialysis | | PM_2.5_-Q4 | | 0.74 | | 0.20,2.70 | | Pneumonia | | PM_2.5_-Q4 | | 0.49 | | 0.43,0.57 | |  | |  | |  | |  | |
| Dialysis | | O_3_-Q1 | | 1 | |  | | Pneumonia | | O_3_-Q1 | | 1 | |  | |  | |  | |  | |  | |
| Dialysis | | O_3_-Q2 | | 0.46 | | 0.21,1.00 | | Pneumonia | | O_3_-Q2 | | 1.44 | | 1.32,1.56 | |  | |  | |  | |  | |
| Dialysis | | O_3_-Q3 | | 0.16 | | 0.05,0.56 | | Pneumonia | | O_3_-Q3 | | 1.60 | | 1.47,1.75 | |  | |  | |  | |  | |
| Dialysis | | O_3_-Q4 | | 0.52 | | 0.18,1.50 | | Pneumonia | | O_3_-Q4 | | 1.60 | | 1.44,1.76 | |  | |  | |  | |  | |

| **Supplement Table 3a**: Risk ratio estimates for continuous chronic air pollution (AP) exposure (2009-2019) and adverse COVID-19 analyses, adjusting for individual NEVI domains^1-2^ [Phase 1: 3/2020-6/2020]   \| **NEVI** \| **Outcome** \| **Pollutant** \| **RR** \| **95% CI** \| **NEVI** \| **Outcome** \| **Pollutant** \| **RR** \| **95%** \| \| --- \| --- \| --- \| --- \| --- \| --- \| --- \| --- \| --- \| --- \| \| Demo \| ARDS \| BC \| 0.98 \| 0.94,1.02 \| Res \| ARDS \| BC \| 0.89 \| 0.84,0.94 \| \| Demo \| ARDS \| NO_2_ \| 1.02 \| 0.96,1.07 \| Res \| ARDS \| NO_2_ \| 1.03 \| 0.96,1.11 \| \| Demo \| ARDS \| PM_2.5_ \| 0.98 \| 0.94,1.03 \| Res \| ARDS \| PM_2.5_ \| 0.93 \| 0.87,1.00 \| \| Demo \| ARDS \| O_3_ \| 1.01 \| 0.99,1.03 \| Res \| ARDS \| O_3_ \| 1.02 \| 0.99,1.05 \| \| Demo \| Dialysis \| BC \| 0.93 \| 0.81,1.07 \| Res \| Dialysis \| BC \| 0.63 \| 0.51,0.78 \| \| Demo \| Dialysis \| NO_2_ \| 1.10 \| 0.95,1.27 \| Res \| Dialysis \| NO_2_ \| 1.19 \| 0.96,1.46 \| \| Demo \| Dialysis \| PM_2.5_ \| 0.97 \| 0.85,1.11 \| Res \| Dialysis \| PM_2.5_ \| 0.80 \| 0.60,1.07 \| \| Demo \| Dialysis \| O_3_ \| 0.99 \| 0.94,1.05 \| Res \| Dialysis \| O_3_ \| 1.02 \| 0.91,1.14 \| \| Demo \| Length of Stay \| BC \| 1.00 \| 0.98,1.02 \| Res \| Length of Stay \| BC \| 1.00 \| 0.98,1.03 \| \| Demo \| Length of Stay \| NO_2_ \| 1.02 \| 0.99,1.05 \| Res \| Length of Stay \| NO_2_ \| 1.02 \| 0.98,1.06 \| \| Demo \| Length of Stay \| PM_2.5_ \| 1.02 \| 0.99,1.04 \| Res \| Length of Stay \| PM_2.5_ \| 1.02 \| 0.99,1.05 \| \| Demo \| Length of Stay \| O_3_ \| 0.99 \| 0.98,1.00 \| Res \| Length of Stay \| O_3_ \| 0.99 \| 0.98,1.01 \| \| Demo \| Pneumonia \| BC \| 0.93 \| 0.88,0.98 \| Res \| Pneumonia \| BC \| 0.76 \| 0.71,0.83 \| \| Demo \| Pneumonia \| NO_2_ \| 1.07 \| 1.00,1.13 \| Res \| Pneumonia \| NO_2_ \| 1.31 \| 1.20,1.43 \| \| Demo \| Pneumonia \| PM_2.5_ \| 0.95 \| 0.90,1.00 \| Res \| Pneumonia \| PM_2.5_ \| 0.91 \| 0.83,1.00 \| \| Demo \| Pneumonia \| O_3_ \| 1.00 \| 0.98,1.02 \| Res \| Pneumonia \| O_3_ \| 0.96 \| 0.92,1.00 \| \| Demo \| Ventilation \| BC \| 0.97 \| 0.93,1.01 \| Res \| Ventilation \| BC \| 0.96 \| 0.91,1.01 \| \| Demo \| Ventilation \| NO_2_ \| 0.93 \| 0.88,0.99 \| Res \| Ventilation \| NO_2_ \| 0.91 \| 0.84,0.98 \| \| Demo \| Ventilation \| PM_2.5_ \| 0.95 \| 0.90,1.00 \| Res \| Ventilation \| PM_2.5_ \| 0.93 \| 0.88,1.00 \| \| Demo \| Ventilation \| O_3_ \| 1.03 \| 1.01,1.05 \| Res \| Ventilation \| O_3_ \| 1.05 \| 1.02,1.08 \| \| Econ \| ARDS \| BC \| 0.96 \| 0.91,1.00 \| Health \| ARDS \| BC \| 0.94 \| 0.90,0.98 \| \| Econ \| ARDS \| NO_2_ \| 1.06 \| 1.00,1.12 \| Health \| ARDS \| NO_2_ \| 1.04 \| 0.98,1.10 \| \| Econ \| ARDS \| PM_2.5_ \| 0.98 \| 0.94,1.04 \| Health \| ARDS \| PM_2.5_ \| 0.97 \| 0.92,1.02 \| \| Econ \| ARDS \| O_3_ \| 1.00 \| 0.98,1.02 \| Health \| ARDS \| O_3_ \| 1.00 \| 0.98,1.02 \| \| Econ \| Dialysis \| BC \| 0.82 \| 0.67,1.00 \| Health \| Dialysis \| BC \| 0.79 \| 0.66,0.94 \| \| Econ \| Dialysis \| NO_2_ \| 1.24 \| 1.06,1.44 \| Health \| Dialysis \| NO_2_ \| 1.18 \| 0.99,1.39 \| \| Econ \| Dialysis \| PM_2.5_ \| 0.97 \| 0.81,1.17 \| Health \| Dialysis \| PM_2.5_ \| 0.91 \| 0.75,1.11 \| \| Econ \| Dialysis \| O_3_ \| 0.97 \| 0.91,1.03 \| Health \| Dialysis \| O_3_ \| 0.99 \| 0.92,1.06 \| \| Econ \| Length of Stay \| BC \| 1.00 \| 0.98,1.02 \| Health \| Length of Stay \| BC \| 1.01 \| 0.98,1.03 \| \| Econ \| Length of Stay \| NO_2_ \| 1.01 \| 0.98,1.05 \| Health \| Length of Stay \| NO_2_ \| 1.02 \| 0.99,1.05 \| \| Econ \| Length of Stay \| PM_2.5_ \| 1.01 \| 0.99,1.04 \| Health \| Length of Stay \| PM_2.5_ \| 1.02 \| 0.99,1.05 \| \| Econ \| Length of Stay \| O_3_ \| 1.00 \| 0.99,1.01 \| Health \| Length of Stay \| O_3_ \| 0.99 \| 0.98,1.00 \| \| Econ \| Pneumonia \| BC \| 0.89 \| 0.84,0.96 \| Health \| Pneumonia \| BC \| 0.83 \| 0.78,0.88 \| \| Econ \| Pneumonia \| NO_2_ \| 1.18 \| 1.12,1.26 \| Health \| Pneumonia \| NO_2_ \| 1.11 \| 1.03,1.19 \| \| Econ \| Pneumonia \| PM_2.5_ \| 0.97 \| 0.91,1.04 \| Health \| Pneumonia \| PM_2.5_ \| 0.89 \| 0.83,0.96 \| \| Econ \| Pneumonia \| O_3_ \| 0.97 \| 0.95,1.00 \| Health \| Pneumonia \| O_3_ \| 1.00 \| 0.98,1.03 \| \| Econ \| Ventilation \| BC \| 0.96 \| 0.92,1.01 \| Health \| Ventilation \| BC \| 0.96 \| 0.92,1.01 \| \| Econ \| Ventilation \| NO_2_ \| 0.93 \| 0.87,0.99 \| Health \| Ventilation \| NO_2_ \| 0.93 \| 0.88,1.00 \| \| Econ \| Ventilation \| PM_2.5_ \| 0.94 \| 0.89,1.00 \| Health \| Ventilation \| PM_2.5_ \| 0.95 \| 0.90,1.00 \| \| Econ \| Ventilation \| O_3_ \| 1.03 \| 1.01,1.05 \| Health \| Ventilation \| O_3_ \| 1.03 \| 1.01,1.05 \|   ^1^ Modified Poisson models for ARDS, dialysis, pneumonia, ventilation; Cox proportional hazards models for length of stay  ^2^ Abbreviations: Demo – NEVI Demographics Score; Econ – NEVI Economics Score; Res – NEVI Residential Score; Health – NEVI Health Score  **Supplement Table 3b**: Risk ratio estimates for continuous chronic air pollution (AP) exposure (2009-2019) and adverse COVID-19 analyses, adjusting for individual NEVI domains^1-2^ [Phases 2 & 3: 7/2020 – 2/2021]   \| **NEVI** \| **Outcome** \| **Pollutant** \| **RR** \| **95% CI** \| **NEVI** \| **Outcome** \| **Pollutant** \| **RR** \| **95%** \| \| --- \| --- \| --- \| --- \| --- \| --- \| --- \| --- \| --- \| --- \| \| Demo \| ARDS \| BC \| 0.92 \| 0.87,0.98 \| Res \| ARDS \| BC \| 0.90 \| 0.84,0.97 \| \| Demo \| ARDS \| NO_2_ \| 0.88 \| 0.80,0.96 \| Res \| ARDS \| NO_2_ \| 0.84 \| 0.75,0.94 \| \| Demo \| ARDS \| PM_2.5_ \| 0.91 \| 0.84,0.98 \| Res \| ARDS \| PM_2.5_ \| 0.89 \| 0.81,0.97 \| \| Demo \| ARDS \| O_3_ \| 1.03 \| 1.00,1.06 \| Res \| ARDS \| O_3_ \| 1.04 \| 1.00,1.08 \| \| Demo \| Dialysis \| BC \| 0.85 \| 0.68,1.08 \| Res \| Dialysis \| BC \| 0.59 \| 0.43,0.80 \| \| Demo \| Dialysis \| NO_2_ \| 0.96 \| 0.75,1.23 \| Res \| Dialysis \| NO_2_ \| 0.76 \| 0.52,1.13 \| \| Demo \| Dialysis \| PM_2.5_ \| 0.88 \| 0.71,1.10 \| Res \| Dialysis \| PM_2.5_ \| 0.62 \| 0.41,0.95 \| \| Demo \| Dialysis \| O_3_ \| 1.02 \| 0.93,1.13 \| Res \| Dialysis \| O_3_ \| 1.13 \| 0.96,1.34 \| \| Demo \| Length of Stay \| BC \| 1.01 \| 0.99,1.04 \| Res \| Length of Stay \| BC \| 1.00 \| 0.97,1.02 \| \| Demo \| Length of Stay \| NO_2_ \| 1.05 \| 1.02,1.08 \| Res \| Length of Stay \| NO_2_ \| 1.07 \| 1.03,1.12 \| \| Demo \| Length of Stay \| PM_2.5_ \| 1.02 \| 1.00,1.05 \| Res \| Length of Stay \| PM_2.5_ \| 1.02 \| 0.99,1.06 \| \| Demo \| Length of Stay \| O_3_ \| 0.99 \| 0.98,1.00 \| Res \| Length of Stay \| O_3_ \| 0.99 \| 0.98,1.00 \| \| Demo \| Pneumonia \| BC \| 0.87 \| 0.85,0.89 \| Res \| Pneumonia \| BC \| 0.89 \| 0.87,0.92 \| \| Demo \| Pneumonia \| NO_2_ \| 0.80 \| 0.77,0.83 \| Res \| Pneumonia \| NO_2_ \| 0.78 \| 0.74,0.81 \| \| Demo \| Pneumonia \| PM_2.5_ \| 0.85 \| 0.82,0.88 \| Res \| Pneumonia \| PM_2.5_ \| 0.86 \| 0.82,0.89 \| \| Demo \| Pneumonia \| O_3_ \| 1.07 \| 1.06,1.08 \| Res \| Pneumonia \| O_3_ \| 1.08 \| 1.06,1.10 \| \| Demo \| Ventilation \| BC \| 0.85 \| 0.79,0.91 \| Res \| Ventilation \| BC \| 0.90 \| 0.83,0.97 \| \| Demo \| Ventilation \| NO_2_ \| 0.72 \| 0.64,0.80 \| Res \| Ventilation \| NO_2_ \| 0.66 \| 0.57,0.75 \| \| Demo \| Ventilation \| PM_2.5_ \| 0.79 \| 0.71,0.87 \| Res \| Ventilation \| PM_2.5_ \| 0.80 \| 0.72,0.89 \| \| Demo \| Ventilation \| O_3_ \| 1.09 \| 1.06,1.13 \| Res \| Ventilation \| O_3_ \| 1.11 \| 1.07,1.16 \| \| Econ \| ARDS \| BC \| 0.91 \| 0.85,0.97 \| Health \| ARDS \| BC \| 0.91 \| 0.86,0.97 \| \| Econ \| ARDS \| NO_2_ \| 0.87 \| 0.79,0.95 \| Health \| ARDS \| NO_2_ \| 0.88 \| 0.80,0.96 \| \| Econ \| ARDS \| PM_2.5_ \| 0.90 \| 0.83,0.97 \| Health \| ARDS \| PM_2.5_ \| 0.91 \| 0.84,0.98 \| \| Econ \| ARDS \| O_3_ \| 1.03 \| 1.00,1.06 \| Health \| ARDS \| O_3_ \| 1.03 \| 1.00,1.06 \| \| Econ \| Dialysis \| BC \| 0.78 \| 0.58,1.04 \| Health \| Dialysis \| BC \| 0.78 \| 0.61,1.00 \| \| Econ \| Dialysis \| NO_2_ \| 1.00 \| 0.76,1.31 \| Health \| Dialysis \| NO_2_ \| 0.95 \| 0.72,1.25 \| \| Econ \| Dialysis \| PM_2.5_ \| 0.86 \| 0.65,1.13 \| Health \| Dialysis \| PM_2.5_ \| 0.82 \| 0.64,1.07 \| \| Econ \| Dialysis \| O_3_ \| 1.01 \| 0.91,1.13 \| Health \| Dialysis \| O_3_ \| 1.03 \| 0.93,1.15 \| \| Econ \| Length of Stay \| BC \| 1.01 \| 0.99,1.03 \| Health \| Length of Stay \| BC \| 1.00 \| 0.98,1.02 \| \| Econ \| Length of Stay \| NO_2_ \| 1.06 \| 1.03,1.09 \| Health \| Length of Stay \| NO_2_ \| 1.05 \| 1.01,1.08 \| \| Econ \| Length of Stay \| PM_2.5_ \| 1.03 \| 1.00,1.05 \| Health \| Length of Stay \| PM_2.5_ \| 1.02 \| 0.99,1.04 \| \| Econ \| Length of Stay \| O_3_ \| 0.99 \| 0.98,1.00 \| Health \| Length of Stay \| O_3_ \| 0.99 \| 0.98,1.00 \| \| Econ \| Pneumonia \| BC \| 0.87 \| 0.85,0.89 \| Health \| Pneumonia \| BC \| 0.88 \| 0.86,0.90 \| \| Econ \| Pneumonia \| PM_2.5_ \| 0.85 \| 0.82,0.88 \| Health \| Pneumonia \| PM_2.5_ \| 0.86 \| 0.83,0.89 \| \| Econ \| Pneumonia \| NO_2_ \| 0.79 \| 0.76,0.82 \| Health \| Pneumonia \| NO_2_ \| 0.80 \| 0.77,0.83 \| \| Econ \| Pneumonia \| O_3_ \| 1.07 \| 1.06,1.09 \| Health \| Pneumonia \| O_3_ \| 1.07 \| 1.06,1.08 \| \| Econ \| Ventilation \| BC \| 0.85 \| 0.79,0.91 \| Health \| Ventilation \| BC \| 0.87 \| 0.82,0.93 \| \| Econ \| Ventilation \| NO_2_ \| 0.66 \| 0.58,0.75 \| Health \| Ventilation \| NO_2_ \| 0.70 \| 0.63,0.79 \| \| Econ \| Ventilation \| PM_2.5_ \| 0.77 \| 0.70,0.85 \| Health \| Ventilation \| PM_2.5_ \| 0.81 \| 0.73,0.89 \| \| Econ \| Ventilation \| O_3_ \| 1.11 \| 1.07,1.15 \| Health \| Ventilation \| O_3_ \| 1.09 \| 1.06,1.13 \|   ^1^ Modified Poisson models for ARDS, dialysis, pneumonia, ventilation; Cox proportional hazards models for length of stay ^2^ Abbreviations: Demo – NEVI Demographics Score; Econ – NEVI Economics Score; Res – NEVI Residential Score; Health – NEVI Health Score  **Supplement Table 3c**: Risk ratio estimates for continuous chronic air pollution (AP) exposure (2009-2019) and adverse COVID-19 analyses, adjusting for individual NEVI domains^1-2^ [Phase 1 – 40% Subset: 3/2020-6/2020]   \| **NEVI** \| **Outcome** \| **Pollutant** \| **RR** \| **95% CI** \| **NEVI** \| **Outcome** \| **Pollutant** \| **RR** \| **95%** \| \| --- \| --- \| --- \| --- \| --- \| --- \| --- \| --- \| --- \| --- \| \| Demo \| ARDS \| BC \| 1.02 \| 0.96,1.09 \| Res \| ARDS \| BC \| 1.01 \| 0.92,1.10 \| \| Demo \| ARDS \| NO_2_ \| 1.07 \| 0.99,1.14 \| Res \| ARDS \| NO_2_ \| 1.14 \| 1.05,1.23 \| \| Demo \| ARDS \| PM_2.5_ \| 1.02 \| 0.96,1.08 \| Res \| ARDS \| PM_2.5_ \| 1.05 \| 0.98,1.13 \| \| Demo \| ARDS \| O_3_ \| 0.99 \| 0.96,1.02 \| Res \| ARDS \| O_3_ \| 0.98 \| 0.94,1.01 \| \| Demo \| Dialysis \| BC \| 1.06 \| 0.87,1.30 \| Res \| Dialysis \| BC \| 0.99 \| 0.68,1.43 \| \| Demo \| Dialysis \| NO_2_ \| 1.21 \| 1.00,1.45 \| Res \| Dialysis \| NO_2_ \| 1.52 \| 1.26,1.83 \| \| Demo \| Dialysis \| PM_2.5_ \| 1.04 \| 0.87,1.24 \| Res \| Dialysis \| PM_2.5_ \| 1.19 \| 0.96,1.48 \| \| Demo \| Dialysis \| O_3_ \| 0.94 \| 0.87,1.00 \| Res \| Dialysis \| O_3_ \| 0.87 \| 0.80,0.95 \| \| Demo \| Length of Stay \| BC \| 0.99 \| 0.96,1.03 \| Res \| Length of Stay \| BC \| 1.00 \| 0.95,1.04 \| \| Demo \| Length of Stay \| NO_2_ \| 1.00 \| 0.96,1.05 \| Res \| Length of Stay \| NO_2_ \| 1.00 \| 0.96,1.05 \| \| Demo \| Length of Stay \| PM_2.5_ \| 1.01 \| 0.97,1.04 \| Res \| Length of Stay \| PM_2.5_ \| 1.01 \| 0.97,1.04 \| \| Demo \| Length of Stay \| O_3_ \| 1.00 \| 0.98,1.01 \| Res \| Length of Stay \| O_3_ \| 1.00 \| 0.98,1.02 \| \| Demo \| Pneumonia \| BC \| 1.23 \| 1.12,1.34 \| Res \| Pneumonia \| BC \| 1.23 \| 1.08,1.39 \| \| Demo \| Pneumonia \| NO_2_ \| 1.31 \| 1.20,1.42 \| Res \| Pneumonia \| NO_2_ \| 1.45 \| 1.33,1.59 \| \| Demo \| Pneumonia \| PM_2.5_ \| 1.14 \| 1.06,1.23 \| Res \| Pneumonia \| PM_2.5_ \| 1.23 \| 1.13,1.33 \| \| Demo \| Pneumonia \| O_3_ \| 0.90 \| 0.87,0.93 \| Res \| Pneumonia \| O_3_ \| 0.87 \| 0.84,0.90 \| \| Demo \| Ventilation \| BC \| 0.92 \| 0.86,0.99 \| Res \| Ventilation \| BC \| 0.91 \| 0.84,0.99 \| \| Demo \| Ventilation \| NO_2_ \| 0.93 \| 0.86,1.00 \| Res \| Ventilation \| NO_2_ \| 0.95 \| 0.87,1.03 \| \| Demo \| Ventilation \| PM_2.5_ \| 0.92 \| 0.85,0.98 \| Res \| Ventilation \| PM_2.5_ \| 0.92 \| 0.85,1.00 \| \| Demo \| Ventilation \| O_3_ \| 1.06 \| 1.02,1.09 \| Res \| Ventilation \| O_3_ \| 1.08 \| 1.03,1.13 \| \| Econ \| ARDS \| BC \| 1.07 \| 1.00,1.14 \| Health \| ARDS \| BC \| 1.06 \| 0.99,1.14 \| \| Econ \| ARDS \| NO_2_ \| 1.16 \| 1.09,1.24 \| Health \| ARDS \| NO_2_ \| 1.17 \| 1.09,1.25 \| \| Econ \| ARDS \| PM_2.5_ \| 1.09 \| 1.02,1.15 \| Health \| ARDS \| PM_2.5_ \| 1.09 \| 1.02,1.16 \| \| Econ \| ARDS \| O_3_ \| 0.96 \| 0.93,0.98 \| Health \| ARDS \| O_3_ \| 0.96 \| 0.93,0.99 \| \| Econ \| Dialysis \| BC \| 1.30 \| 0.99,1.71 \| Health \| Dialysis \| BC \| 1.27 \| 0.94,1.72 \| \| Econ \| Dialysis \| NO_2_ \| 1.73 \| 1.45,2.05 \| Health \| Dialysis \| NO_2_ \| 1.82 \| 1.46,2.27 \| \| Econ \| Dialysis \| PM_2.5_ \| 1.38 \| 1.14,1.67 \| Health \| Dialysis \| PM_2.5_ \| 1.40 \| 1.10,1.77 \| \| Econ \| Dialysis \| O_3_ \| 0.83 \| 0.77,0.88 \| Health \| Dialysis \| O_3_ \| 0.81 \| 0.74,0.88 \| \| Econ \| Length of Stay \| BC \| 0.99 \| 0.95,1.02 \| Health \| Length of Stay \| BC \| 1.00 \| 0.96,1.03 \| \| Econ \| Length of Stay \| NO_2_ \| 0.99 \| 0.96,1.03 \| Health \| Length of Stay \| NO_2_ \| 1.00 \| 0.96,1.04 \| \| Econ \| Length of Stay \| PM_2.5_ \| 1.00 \| 0.97,1.04 \| Health \| Length of Stay \| PM_2.5_ \| 1.01 \| 0.98,1.05 \| \| Econ \| Length of Stay \| O_3_ \| 1.00 \| 0.99,1.02 \| Health \| Length of Stay \| O_3_ \| 1.00 \| 0.98,1.01 \| \| Econ \| Pneumonia \| BC \| 1.47 \| 1.33,1.63 \| Health \| Pneumonia \| BC \| 1.42 \| 1.27,1.59 \| \| Econ \| Pneumonia \| PM_2.5_ \| 1.67 \| 1.54,1.81 \| Health \| Pneumonia \| PM_2.5_ \| 1.67 \| 1.51,1.85 \| \| Econ \| Pneumonia \| NO_2_ \| 1.40 \| 1.30,1.51 \| Health \| Pneumonia \| NO_2_ \| 1.37 \| 1.25,1.50 \| \| Econ \| Pneumonia \| O_3_ \| 0.83 \| 0.80,0.85 \| Health \| Pneumonia \| O_3_ \| 0.82 \| 0.79,0.86 \| \| Econ \| Ventilation \| BC \| 0.92 \| 0.85,0.99 \| Health \| Ventilation \| BC \| 0.93 \| 0.86,1.00 \| \| Econ \| Ventilation \| NO_2_ \| 0.94 \| 0.87,1.02 \| Health \| Ventilation \| NO_2_ \| 0.96 \| 0.88,1.04 \| \| Econ \| Ventilation \| PM_2.5_ \| 0.93 \| 0.86,1.00 \| Health \| Ventilation \| PM_2.5_ \| 0.94 \| 0.87,1.02 \| \| Econ \| Ventilation \| O_3_ \| 1.05 \| 1.02,1.08 \| Health \| Ventilation \| O_3_ \| 1.04 \| 1.01,1.08 \|   ^1^ Modified Poisson models for ARDS, dialysis, pneumonia, ventilation; Cox proportional hazards models for length of stay  ^2^ Abbreviations: Demo – NEVI Demographics Score; Econ – NEVI Economics Score; Res – NEVI Residential Score; Health – NEVI Health Score  **Supplement Table 3d**: Risk ratio estimates for continuous chronic air pollution (AP) exposure (2009-2019) and adverse COVID-19 analyses, adjusting for individual NEVI domains^1-2^ [Phases 2 & 3 – 40% Subset: 7/2020 – 2/2021]   \| **NEVI** \| **Outcome** \| **Pollutant** \| **RR** \| **95% CI** \| **NEVI** \| **Outcome** \| **Pollutant** \| **RR** \| **95%** \| \| --- \| --- \| --- \| --- \| --- \| --- \| --- \| --- \| --- \| --- \| \| Demo \| ARDS \| BC \| 0.90 \| 0.82,0.97 \| Res \| ARDS \| BC \| 0.88 \| 0.80,0.97 \| \| Demo \| ARDS \| NO_2_ \| 0.90 \| 0.82,1.00 \| Res \| ARDS \| NO_2_ \| 0.89 \| 0.79,1.01 \| \| Demo \| ARDS \| PM_2.5_ \| 0.91 \| 0.84,1.00 \| Res \| ARDS \| PM_2.5_ \| 0.91 \| 0.82,1.00 \| \| Demo \| ARDS \| O_3_ \| 1.03 \| 0.99,1.06 \| Res \| ARDS \| O_3_ \| 1.03 \| 0.98,1.08 \| \| Demo \| Dialysis \| BC \| 1.15 \| 0.83,1.59 \| Res \| Dialysis \| BC \| 0.93 \| 0.59,1.47 \| \| Demo \| Dialysis \| NO_2_ \| 1.20 \| 0.89,1.61 \| Res \| Dialysis \| NO_2_ \| 1.02 \| 0.72,1.45 \| \| Demo \| Dialysis \| PM_2.5_ \| 1.05 \| 0.81,1.36 \| Res \| Dialysis \| PM_2.5_ \| 0.89 \| 0.62,1.28 \| \| Demo \| Dialysis \| O_3_ \| 0.95 \| 0.84,1.07 \| Res \| Dialysis \| O_3_ \| 1.03 \| 0.87,1.23 \| \| Demo \| Length of Stay \| BC \| 1.02 \| 0.99,1.06 \| Res \| Length of Stay \| BC \| 1.03 \| 0.99,1.07 \| \| Demo \| Length of Stay \| NO_2_ \| 1.05 \| 1.01,1.09 \| Res \| Length of Stay \| NO_2_ \| 1.08 \| 1.04,1.13 \| \| Demo \| Length of Stay \| PM_2.5_ \| 1.03 \| 0.99,1.06 \| Res \| Length of Stay \| PM_2.5_ \| 1.04 \| 1.01,1.08 \| \| Demo \| Length of Stay \| O_3_ \| 0.99 \| 0.98,1.00 \| Res \| Length of Stay \| O_3_ \| 0.98 \| 0.96,0.99 \| \| Demo \| Pneumonia \| BC \| 0.82 \| 0.80,0.85 \| Res \| Pneumonia \| BC \| 0.83 \| 0.80,0.86 \| \| Demo \| Pneumonia \| NO_2_ \| 0.78 \| 0.75,0.82 \| Res \| Pneumonia \| NO_2_ \| 0.77 \| 0.73,0.82 \| \| Demo \| Pneumonia \| PM_2.5_ \| 0.83 \| 0.80,0.87 \| Res \| Pneumonia \| PM_2.5_ \| 0.83 \| 0.79,0.87 \| \| Demo \| Pneumonia \| O_3_ \| 1.09 \| 1.07,1.11 \| Res \| Pneumonia \| O_3_ \| 1.11 \| 1.09,1.13 \| \| Demo \| Ventilation \| BC \| 0.72 \| 0.65,0.79 \| Res \| Ventilation \| BC \| 0.75 \| 0.67,0.83 \| \| Demo \| Ventilation \| NO_2_ \| 0.64 \| 0.55,0.73 \| Res \| Ventilation \| NO_2_ \| 0.62 \| 0.52,0.73 \| \| Demo \| Ventilation \| PM_2.5_ \| 0.68 \| 0.59,0.78 \| Res \| Ventilation \| PM_2.5_ \| 0.69 \| 0.59,0.79 \| \| Demo \| Ventilation \| O_3_ \| 1.16 \| 1.11,1.21 \| Res \| Ventilation \| O_3_ \| 1.20 \| 1.13,1.27 \| \| Econ \| ARDS \| BC \| 0.88 \| 0.81,0.97 \| Health \| ARDS \| BC \| 0.91 \| 0.83,0.99 \| \| Econ \| ARDS \| NO_2_ \| 0.90 \| 0.81,1.00 \| Health \| ARDS \| NO_2_ \| 0.93 \| 0.83,1.04 \| \| Econ \| ARDS \| PM_2.5_ \| 0.92 \| 0.84,1.01 \| Health \| ARDS \| PM_2.5_ \| 0.94 \| 0.85,1.04 \| \| Econ \| ARDS \| O_3_ \| 1.03 \| 0.99,1.06 \| Health \| ARDS \| O_3_ \| 1.01 \| 0.98,1.05 \| \| Econ \| Dialysis \| BC \| 1.21 \| 0.84,1.75 \| Health \| Dialysis \| BC \| 1.22 \| 0.82,1.81 \| \| Econ \| Dialysis \| NO_2_ \| 1.34 \| 1.00,1.78 \| Health \| Dialysis \| NO_2_ \| 1.37 \| 0.98,1.92 \| \| Econ \| Dialysis \| PM_2.5_ \| 1.15 \| 0.87,1.51 \| Health \| Dialysis \| PM_2.5_ \| 1.15 \| 0.84,1.57 \| \| Econ \| Dialysis \| O_3_ \| 0.91 \| 0.80,1.03 \| Health \| Dialysis \| O_3_ \| 0.90 \| 0.78,1.04 \| \| Econ \| Length of Stay \| BC \| 1.03 \| 1.00,1.06 \| Health \| Length of Stay \| BC \| 1.02 \| 0.99,1.06 \| \| Econ \| Length of Stay \| NO_2_ \| 1.06 \| 1.03,1.10 \| Health \| Length of Stay \| NO_2_ \| 1.06 \| 1.02,1.10 \| \| Econ \| Length of Stay \| PM_2.5_ \| 1.04 \| 1.00,1.07 \| Health \| Length of Stay \| PM_2.5_ \| 1.03 \| 0.99,1.06 \| \| Econ \| Length of Stay \| O_3_ \| 0.98 \| 0.97,1.00 \| Health \| Length of Stay \| O_3_ \| 0.99 \| 0.97,1.00 \| \| Econ \| Pneumonia \| BC \| 0.82 \| 0.79,0.85 \| Health \| Pneumonia \| BC \| 0.83 \| 0.80,0.86 \| \| Econ \| Pneumonia \| PM_2.5_ \| 0.78 \| 0.75,0.82 \| Health \| Pneumonia \| PM_2.5_ \| 0.79 \| 0.76,0.83 \| \| Econ \| Pneumonia \| NO_2_ \| 0.83 \| 0.79,0.86 \| Health \| Pneumonia \| NO_2_ \| 0.84 \| 0.80,0.87 \| \| Econ \| Pneumonia \| O_3_ \| 1.09 \| 1.07,1.11 \| Health \| Pneumonia \| O_3_ \| 1.09 \| 1.07,1.10 \| \| Econ \| Ventilation \| BC \| 0.70 \| 0.63,0.78 \| Health \| Ventilation \| BC \| 0.73 \| 0.65,0.81 \| \| Econ \| Ventilation \| NO_2_ \| 0.60 \| 0.51,0.70 \| Health \| Ventilation \| NO_2_ \| 0.62 \| 0.53,0.73 \| \| Econ \| Ventilation \| PM_2.5_ \| 0.66 \| 0.57,0.76 \| Health \| Ventilation \| PM_2.5_ \| 0.68 \| 0.59,0.79 \| \| Econ \| Ventilation \| O_3_ \| 1.18 \| 1.12,1.24 \| Health \| Ventilation \| O_3_ \| 1.16 \| 1.10,1.22 \|   ^1^ Modified Poisson models for ARDS, dialysis, pneumonia, ventilation; Cox proportional hazards models for length of stay  ^2^ Abbreviations: Demo – NEVI Demographics Score; Econ – NEVI Economics Score; Res – NEVI Residential Score; Health – NEVI Health Score |
| --- | --- | --- | --- | --- | --- | --- | --- | --- | --- | --- | --- | --- | --- | --- | --- | --- | --- | --- | --- | --- | --- | --- | --- | --- | --- | --- | --- | --- | --- | --- | --- | --- | --- | --- | --- | --- | --- | --- | --- | --- | --- | --- | --- | --- | --- | --- | --- | --- | --- | --- | --- | --- | --- | --- | --- | --- | --- | --- | --- | --- | --- | --- | --- | --- | --- | --- | --- | --- | --- | --- | --- | --- | --- | --- | --- | --- | --- | --- | --- | --- | --- | --- | --- | --- | --- | --- | --- | --- | --- | --- | --- | --- | --- | --- | --- | --- | --- | --- | --- | --- | --- | --- | --- | --- | --- | --- | --- | --- | --- | --- | --- | --- | --- | --- | --- | --- | --- | --- | --- | --- | --- | --- | --- | --- | --- | --- | --- | --- | --- | --- | --- | --- | --- | --- | --- | --- | --- | --- | --- | --- | --- | --- | --- | --- | --- | --- | --- | --- | --- | --- | --- | --- | --- | --- | --- | --- | --- | --- | --- | --- | --- | --- | --- | --- | --- | --- | --- | --- | --- | --- | --- | --- | --- | --- | --- | --- | --- | --- | --- | --- | --- | --- | --- | --- | --- | --- | --- | --- | --- | --- | --- | --- | --- | --- | --- | --- | --- | --- | --- | --- | --- | --- | --- | --- | --- | --- | --- | --- | --- | --- | --- | --- | --- | --- | --- | --- | --- | --- | --- | --- | --- | --- | --- | --- | --- | --- | --- | --- | --- | --- | --- | --- | --- | --- | --- | --- | --- | --- | --- | --- | --- | --- | --- | --- | --- | --- | --- | --- | --- | --- | --- | --- | --- | --- | --- | --- | --- | --- | --- | --- | --- | --- | --- | --- | --- | --- | --- | --- | --- | --- | --- | --- | --- | --- | --- | --- | --- | --- | --- | --- | --- | --- | --- | --- | --- | --- | --- | --- | --- | --- | --- | --- | --- | --- | --- | --- | --- | --- | --- | --- | --- | --- | --- | --- | --- | --- | --- | --- | --- | --- | --- | --- | --- | --- | --- | --- | --- | --- | --- | --- | --- | --- | --- | --- | --- | --- | --- | --- | --- | --- | --- | --- | --- | --- | --- | --- | --- | --- | --- | --- | --- | --- | --- | --- | --- | --- | --- | --- | --- | --- | --- | --- | --- | --- | --- | --- | --- | --- | --- | --- | --- | --- | --- | --- | --- | --- | --- | --- | --- | --- | --- | --- | --- | --- | --- | --- | --- | --- | --- | --- | --- | --- | --- | --- | --- | --- | --- | --- | --- | --- | --- | --- | --- | --- | --- | --- | --- | --- | --- | --- | --- | --- | --- | --- | --- | --- | --- | --- | --- | --- | --- | --- | --- | --- | --- | --- | --- | --- | --- | --- | --- | --- | --- | --- | --- | --- | --- | --- | --- | --- | --- | --- | --- | --- | --- | --- | --- | --- | --- | --- | --- | --- | --- | --- | --- | --- | --- | --- | --- | --- | --- | --- | --- | --- | --- | --- | --- | --- | --- | --- | --- | --- | --- | --- | --- | --- | --- | --- | --- | --- | --- | --- | --- | --- | --- | --- | --- | --- | --- | --- | --- | --- | --- | --- | --- | --- | --- | --- | --- | --- | --- | --- | --- | --- | --- | --- | --- | --- | --- | --- | --- | --- | --- | --- | --- | --- | --- | --- | --- | --- | --- | --- | --- | --- | --- | --- | --- | --- | --- | --- | --- | --- | --- | --- | --- | --- | --- | --- | --- | --- | --- | --- | --- | --- | --- | --- | --- | --- | --- | --- | --- | --- | --- | --- | --- | --- | --- | --- | --- | --- | --- | --- | --- | --- | --- | --- | --- | --- | --- | --- | --- | --- | --- | --- | --- | --- | --- | --- | --- | --- | --- | --- | --- | --- | --- | --- | --- | --- | --- | --- | --- | --- | --- | --- | --- | --- | --- | --- | --- | --- | --- | --- | --- | --- | --- | --- | --- | --- | --- | --- | --- | --- | --- | --- | --- | --- | --- | --- | --- | --- | --- | --- | --- | --- | --- | --- | --- | --- | --- | --- | --- | --- | --- | --- | --- | --- | --- | --- | --- | --- | --- | --- | --- | --- | --- | --- | --- | --- | --- | --- | --- | --- | --- | --- | --- | --- | --- | --- | --- | --- | --- | --- | --- | --- | --- | --- | --- | --- | --- | --- | --- | --- | --- | --- | --- | --- | --- | --- | --- | --- | --- | --- | --- | --- | --- | --- | --- | --- | --- | --- | --- | --- | --- | --- | --- | --- | --- | --- | --- | --- | --- | --- | --- | --- | --- | --- | --- | --- | --- | --- | --- | --- | --- | --- | --- | --- | --- | --- | --- | --- | --- | --- | --- | --- | --- | --- | --- | --- | --- | --- | --- | --- | --- | --- | --- | --- | --- | --- | --- | --- | --- | --- | --- | --- | --- | --- | --- | --- | --- | --- | --- | --- | --- | --- | --- | --- | --- | --- | --- | --- | --- | --- | --- | --- | --- | --- | --- | --- | --- | --- | --- | --- | --- | --- | --- | --- | --- | --- | --- | --- | --- | --- | --- | --- | --- | --- | --- | --- | --- | --- | --- | --- | --- | --- | --- | --- | --- | --- | --- | --- | --- | --- | --- | --- | --- | --- | --- | --- | --- | --- | --- | --- | --- | --- | --- | --- | --- | --- | --- | --- | --- | --- | --- | --- | --- | --- | --- | --- | --- | --- | --- | --- | --- | --- | --- | --- | --- | --- | --- | --- | --- | --- | --- | --- | --- | --- | --- | --- | --- | --- | --- | --- | --- | --- | --- | --- | --- | --- | --- | --- | --- | --- | --- | --- | --- | --- | --- | --- | --- | --- | --- | --- | --- | --- | --- | --- | --- | --- | --- | --- | --- | --- | --- | --- | --- | --- | --- | --- | --- | --- | --- | --- | --- | --- | --- | --- | --- | --- | --- | --- | --- | --- | --- | --- | --- | --- | --- | --- | --- | --- | --- | --- | --- | --- | --- | --- | --- | --- | --- | --- | --- | --- | --- | --- | --- | --- | --- | --- | --- | --- | --- | --- | --- | --- | --- | --- | --- | --- | --- | --- | --- | --- | --- | --- | --- | --- | --- | --- | --- | --- | --- | --- | --- | --- | --- | --- | --- | --- | --- | --- | --- | --- | --- | --- | --- | --- | --- | --- | --- | --- | --- | --- | --- | --- | --- | --- | --- | --- | --- | --- | --- | --- | --- | --- | --- | --- | --- | --- | --- | --- | --- | --- | --- | --- | --- | --- | --- | --- | --- | --- | --- | --- | --- | --- | --- | --- | --- | --- | --- | --- | --- | --- | --- | --- | --- | --- | --- | --- | --- | --- | --- | --- | --- | --- | --- | --- | --- | --- | --- | --- | --- | --- | --- | --- | --- | --- | --- | --- | --- | --- | --- | --- | --- | --- | --- | --- | --- | --- | --- | --- | --- | --- | --- | --- | --- | --- | --- | --- | --- | --- | --- | --- | --- | --- | --- | --- | --- | --- | --- | --- | --- | --- | --- | --- | --- | --- | --- | --- | --- | --- | --- | --- | --- | --- | --- | --- | --- | --- | --- | --- | --- | --- | --- | --- | --- | --- | --- | --- | --- | --- | --- | --- | --- | --- | --- | --- | --- | --- | --- | --- | --- | --- | --- | --- | --- | --- | --- | --- | --- | --- | --- | --- | --- | --- | --- | --- | --- | --- | --- | --- | --- | --- | --- | --- | --- | --- | --- | --- | --- | --- | --- | --- | --- | --- | --- | --- | --- | --- | --- | --- | --- | --- | --- | --- | --- | --- | --- | --- | --- | --- | --- | --- | --- | --- | --- | --- | --- | --- | --- | --- | --- | --- | --- | --- | --- | --- | --- | --- | --- | --- | --- | --- | --- | --- | --- | --- | --- | --- | --- | --- | --- | --- | --- | --- | --- | --- | --- | --- | --- | --- | --- | --- | --- | --- | --- | --- | --- | --- | --- | --- | --- | --- | --- | --- | --- | --- | --- | --- | --- | --- | --- | --- | --- | --- | --- | --- | --- | --- | --- | --- | --- | --- | --- | --- | --- | --- | --- | --- | --- | --- | --- | --- | --- | --- | --- | --- | --- | --- | --- | --- | --- | --- | --- | --- | --- | --- | --- | --- | --- | --- | --- | --- | --- | --- | --- | --- | --- | --- | --- | --- | --- | --- | --- | --- | --- | --- | --- | --- | --- | --- | --- | --- | --- | --- | --- | --- | --- | --- | --- | --- | --- | --- | --- | --- | --- | --- | --- | --- | --- | --- | --- | --- | --- | --- | --- | --- | --- | --- | --- | --- | --- | --- | --- | --- | --- | --- | --- | --- | --- | --- | --- | --- | --- | --- | --- | --- | --- | --- | --- | --- | --- | --- | --- | --- | --- | --- | --- | --- | --- | --- | --- | --- | --- | --- | --- | --- | --- | --- | --- | --- | --- | --- | --- | --- | --- | --- | --- | --- | --- | --- | --- | --- | --- | --- | --- | --- | --- | --- | --- | --- | --- | --- | --- | --- | --- | --- | --- | --- | --- | --- | --- | --- | --- | --- | --- | --- | --- | --- | --- | --- | --- | --- | --- | --- | --- | --- | --- | --- | --- | --- | --- | --- | --- | --- | --- | --- | --- | --- | --- | --- | --- | --- | --- | --- | --- | --- | --- | --- | --- | --- | --- | --- | --- | --- | --- | --- | --- | --- | --- | --- | --- | --- | --- | --- | --- | --- | --- | --- | --- | --- | --- | --- | --- | --- | --- | --- | --- | --- | --- | --- | --- | --- | --- | --- | --- | --- | --- | --- | --- | --- | --- | --- | --- | --- | --- | --- | --- | --- | --- | --- | --- | --- | --- | --- | --- | --- | --- | --- | --- | --- | --- | --- | --- | --- | --- | --- | --- | --- | --- | --- | --- | --- | --- | --- | --- | --- | --- | --- | --- | --- | --- | --- | --- | --- | --- | --- | --- | --- | --- | --- | --- | --- | --- | --- | --- | --- | --- | --- | --- | --- | --- | --- | --- | --- | --- | --- | --- | --- | --- | --- | --- | --- | --- | --- | --- | --- | --- | --- | --- | --- | --- | --- | --- | --- | --- | --- | --- | --- | --- | --- | --- | --- | --- | --- | --- | --- | --- | --- | --- | --- | --- | --- | --- | --- | --- | --- | --- | --- | --- | --- | --- | --- | --- | --- | --- | --- | --- | --- | --- | --- | --- | --- | --- | --- | --- | --- | --- | --- | --- | --- | --- | --- | --- | --- | --- | --- | --- | --- | --- | --- | --- | --- | --- | --- | --- | --- | --- | --- | --- | --- | --- | --- | --- | --- | --- | --- | --- | --- | --- | --- | --- | --- | --- | --- | --- | --- | --- | --- | --- | --- | --- | --- | --- | --- | --- | --- | --- | --- | --- | --- | --- | --- | --- | --- | --- | --- | --- | --- | --- | --- |

**Supplement Table 4:** Dispersion statistics for Poisson regression models examining the relationship between chronic ambient air pollution exposure (2009-2019) and adverse COVID-19 morbidities, including ARDS, dialysis, pneumonia, and ventilation^1-3^

| **Outcome** | **Pollutant** | **Phase** | | | |
| --- | --- | --- | --- | --- | --- |
|  |  | **Phase 1** | **Phase 1 – 40% Subset** | **Phase 2 & 3** | **Phase 2 & 3 – 40% Subset** |
| ARDS | BC | 0.571 | 0.568 | 0.457 | 0.478 |
|  | NO_2_ | 0.571 | 0.567 | 0.457 | 0.479 |
|  | PM_2.5_ | 0.571 | 0.568 | 0.457 | 0.479 |
|  | O_3_ | 0.571 | 0.567 | 0.457 | 0.479 |
| Dialysis | BC | 0.147 | 0.125 | 0.079 | 0.068 |
|  | NO_2_ | 0.148 | 0.123 | 0.08 | 0.068 |
|  | PM_2.5_ | 0.148 | 0.125 | 0.08 | 0.068 |
|  | O_3_ | 0.148 | 0.124 | 0.08 | 0.068 |
| Pneumonia | BC | 0.435 | 0.34 | 0.683 | 0.674 |
|  | NO_2_ | 0.434 | 0.335 | 0.679 | 0.673 |
|  | PM_2.5_ | 0.436 | 0.341 | 0.682 | 0.676 |
|  | O_3_ | 0.436 | 0.335 | 0.681 | 0.673 |
| Ventilation | BC | 0.559 | 0.583 | 0.378 | 0.415 |
|  | NO_2_ | 0.559 | 0.583 | 0.375 | 0.413 |
|  | PM_2.5_ | 0.559 | 0.583 | 0.377 | 0.415 |
|  | O_3_ | 0.559 | 0.582 | 0.377 | 0.414 |

^1^ Dispersion statistic represents the ratio of residual deviance to corresponding degrees of freedom (must be less than 1 to suggest no overdispersion)

^2^ Poisson regression models adjusted for age, sex, body mass index (BMI), smoking status, asthma, diabetes, hypertension, and neighborhood environmental vulnerability index (NEVI)

^3^ Abbreviations: Phase 1 – 3/2020-6/2020; Phases 2 & 3 – 7/2020-2/2021; 40% Subset – greater hospital catchment

**Supplement Table 5:** Risk ratio estimates for the relationship between chronic air pollution exposure (2009-2019) and length of COVID-19 hospitalization stay, stratified by phase and tertile of neighborhood environmental vulnerability index (NEVI)^1-2^

| Outcome | Poll. | NEVI  (Crude, Adjusted, Stratified) | Phase 1  Estimate (95%CI) | p-value for EMM^b^ | Phase 1  40% Subset Estimate (95%CI) | p-value  for EMM^b^ | Phases 2 & 3  Estimate (95%CI) | p-value  for EMM^b^ | Phases 2 & 3  40% Subset  Estimate (95%CI) | p-value for EMM^b^ |
| --- | --- | --- | --- | --- | --- | --- | --- | --- | --- | --- |
| Length of Stay | BC | Crude | 1 (0.98, 1.02) |  | 0.99 (0.95, 1.02) |  | 0.99 (0.97, 1.01) |  | 1.03 (1, 1.06) |  |
|  |  | Adj. NEVI | 1 (0.98, 1.02) |  | 0.99 (0.96, 1.02) |  | 1.01 (0.99, 1.04) |  | 1.03 (1, 1.06) |  |
|  |  | NEVI (T1) | 1 (0.96, 1.03) | 0.661 | 1 (0.95, 1.05) | 0.232 | 1 (0.96, 1.04) | 0.139 | 0.99 (0.94, 1.04) | 0.006 |
|  |  | NEVI (T2) | 1.02 (0.98, 1.06) |  | 1.04 (0.96, 1.12) |  | 1.01 (0.97, 1.05) |  | 1.07 (1.01, 1.14) |  |
|  |  | NEVI (T3) | 1.01 (0.97, 1.05) |  | 0.96 (0.9, 1.02) |  | 1.01 (0.97, 1.05) |  | 1.03 (0.98, 1.09) |  |
| Length of Stay | NO₂ | Crude | 1.02 (0.99, 1.05) |  | 0.99 (0.96, 1.03) |  | 1.05 (1.02, 1.08) |  | 1.07 (1.03, 1.11) |  |
|  |  | Adj. NEVI | 1.02 (0.99, 1.05) |  | 1 (0.96, 1.03) |  | 1.06 (1.03, 1.09) |  | 1.06 (1.02, 1.1) |  |
|  |  | NEVI (T1) | 1 (0.95, 1.04) | 0.219 | 1 (0.95, 1.05) | 0.472 | 1.01 (0.96, 1.05) | <0.001 | 1.01 (0.96, 1.07) | 0.001 |
|  |  | NEVI (T2) | 1.06 (1, 1.12) |  | 1.04 (0.94, 1.14) |  | 1.1 (1.03, 1.16) |  | 1.12 (1.04, 1.2) |  |
|  |  | NEVI (T3) | 1.03 (0.97, 1.1) |  | 0.97 (0.9, 1.05) |  | 1.13 (1.06, 1.21) |  | 1.12 (1.03, 1.21) |  |
| Length of Stay | O₃ | Crude | 1 (0.99, 1.01) |  | 1.01 (0.99, 1.02) |  | 0.99 (0.98, 1) |  | 0.98 (0.97, 0.99) |  |
|  |  | Adj. NEVI | 1 (0.99, 1.01) |  | 1 (0.99, 1.02) |  | 0.99 (0.98, 1) |  | 0.98 (0.97, 1) |  |
|  |  | NEVI (T1) | 1 (0.98, 1.01) | 0.836 | 0.99 (0.97, 1.01) | 0.023 | 0.99 (0.98, 1.01) | 0.112 | 0.99 (0.97, 1.01) | 0.135 |
|  |  | NEVI (T2) | 0.98 (0.96, 1) |  | 0.98 (0.94, 1.01) |  | 0.99 (0.97, 1) |  | 0.97 (0.94, 0.99) |  |
|  |  | NEVI (T3) | 1 (0.98, 1.02) |  | 1.03 (1, 1.06) |  | 0.99 (0.97, 1.01) |  | 0.99 (0.96, 1.02) |  |
| Length of Stay | PM_2.5_ | Crude | 1.01 (0.99, 1.04) |  | 1 (0.97, 1.03) |  | 1.02 (0.99, 1.05) |  | 1.05 (1.01, 1.08) |  |
|  |  | Adj. NEVI | 1.02 (0.99, 1.04) |  | 1 (0.97, 1.04) |  | 1.03 (1, 1.05) |  | 1.03 (1, 1.07) |  |
|  |  | NEVI (T1) | 1 (0.97, 1.04) | 0.144 | 1 (0.96, 1.04) | 0.9 | 1 (0.97, 1.04) | 0.012 | 1 (0.96, 1.05) | 0.004 |
|  |  | NEVI (T2) | 1.04 (0.99, 1.09) |  | 1.05 (0.97, 1.14) |  | 1.04 (0.99, 1.09) |  | 1.09 (1.02, 1.16) |  |
|  |  | NEVI (T3) | 1.04 (0.99, 1.1) |  | 0.99 (0.91, 1.08) |  | 1.05 (0.99, 1.11) |  | 1.06 (0.98, 1.15) |  |

^1^ Cox proportional hazards regression models adjusted for age, sex, body mass index (BMI), smoking status, asthma, diabetes and hypertension

^2^ Abbreviations: PM_2.5_ – fine particulate matter; BC – black carbon; NO_2_ – nitrogen dioxide; O_3_ – ozone; NEVI – Neighborhood Environmental Vulnerability Index; EMM – effect measure modification by NEVI; Crude – Unadjusted for NEVI; Adj. NEVI – Adjusted for NEVI; T1/T2/T3 – NEVI Tertile strata (NEVI T1 identified as lowest tertile); Phase 1 – 3/2020-6/2020; Phases 2 & 3 – 7/2020-2/2021; 40% Subset – greater hospital catchment

**Supplement Table 6:** Risk ratio estimates for the relationship between chronic air pollution exposure (2009-2019) and ARDS risk, stratified by phase and tertile of neighborhood environmental vulnerability index (NEVI)^1-2^

| Outcome | Poll. | NEVI  (Crude, Adjusted, Stratified) | Phase 1  Estimate (95%CI) | p-value for EMM^b^ | Phase 1  40% Subset Estimate (95%CI) | p-value  for EMM^b^ | Phases 2 & 3  Estimate (95%CI) | p-value  for EMM^b^ | Phases 2 & 3  40% Subset  Estimate (95%CI) | p-value for EMM^b^ |
| --- | --- | --- | --- | --- | --- | --- | --- | --- | --- | --- |
| ARDS | BC | Crude | 0.92 (0.88, 0.97) |  | 1.03 (0.95, 1.1) |  | 0.9 (0.85, 0.95) |  | 0.87 (0.8, 0.94) |  |
|  |  | Adj. NEVI | 0.96 (0.91, 1) |  | 1.07 (1, 1.14) |  | 0.91 (0.85, 0.97) |  | 0.89 (0.81, 0.97) |  |
|  |  | NEVI (T1) | 0.98 (0.91, 1.05) | 0.255 | 0.97 (0.87, 1.07) | 0.062 | 0.91 (0.82, 1.02) | 0.181 | 0.88 (0.76, 1.03) | 0.588 |
|  |  | NEVI (T2) | 0.92 (0.84, 1.01) |  | 1.1 (0.93, 1.31) |  | 0.93 (0.84, 1.04) |  | 0.92 (0.77, 1.09) |  |
|  |  | NEVI (T3) | 0.92 (0.86, 0.99) |  | 1.16 (1.04, 1.31) |  | 0.9 (0.82, 0.99) |  | 0.91 (0.8, 1.03) |  |
| ARDS | NO₂ | Crude | 1.02 (0.96, 1.08) |  | 1.12 (1.05, 1.2) |  | 0.84 (0.77, 0.92) |  | 0.87 (0.78, 0.96) |  |
|  |  | Adj. NEVI | 1.06 (1, 1.11) |  | 1.16 (1.09, 1.24) |  | 0.87 (0.79, 0.96) |  | 0.91 (0.82, 1.01) |  |
|  |  | NEVI (T1) | 1.02 (0.95, 1.11) | 0.467 | 1.04 (0.95, 1.14) | 0.009 | 0.89 (0.78, 1.02) | 0.072 | 0.89 (0.76, 1.04) | 0.622 |
|  |  | NEVI (T2) | 1.05 (0.94, 1.17) |  | 1.31 (1.12, 1.54) |  | 0.95 (0.81, 1.11) |  | 0.99 (0.82, 1.18) |  |
|  |  | NEVI (T3) | 1.07 (0.95, 1.2) |  | 1.35 (1.15, 1.57) |  | 0.76 (0.65, 0.9) |  | 0.84 (0.68, 1.04) |  |
| ARDS | O₃ | Crude | 1.01 (0.99, 1.03) |  | 0.98 (0.95, 1) |  | 1.04 (1.01, 1.07) |  | 1.04 (1.01, 1.08) |  |
|  |  | Adj. NEVI | 1 (0.98, 1.02) |  | 0.96 (0.94, 0.98) |  | 1.03 (1, 1.06) |  | 1.03 (0.99, 1.06) |  |
|  |  | NEVI (T1) | 1 (0.97, 1.03) | 0.583 | 1 (0.96, 1.04) | 0.006 | 1.04 (1, 1.1) | 0.766 | 1.04 (0.99, 1.11) | 0.88 |
|  |  | NEVI (T2) | 1.01 (0.97, 1.06) |  | 0.94 (0.88, 1) |  | 1.01 (0.96, 1.07) |  | 0.99 (0.93, 1.05) |  |
|  |  | NEVI (T3) | 1 (0.96, 1.04) |  | 0.91 (0.86, 0.97) |  | 1.04 (0.99, 1.09) |  | 1.03 (0.97, 1.1) |  |
| ARDS | PM_2.5_ | Crude | 0.95 (0.9, 1.01) |  | 1.05 (0.99, 1.12) |  | 0.88 (0.81, 0.95) |  | 0.88 (0.81, 0.96) |  |
|  |  | Adj. NEVI | 0.98 (0.94, 1.03) |  | 1.08 (1.02, 1.15) |  | 0.9 (0.83, 0.97) |  | 0.92 (0.84, 1.01) |  |
|  |  | NEVI (T1) | 0.99 (0.93, 1.06) | 0.252 | 1 (0.92, 1.08) | 0.008 | 0.92 (0.83, 1.03) | 0.06 | 0.91 (0.8, 1.04) | 0.845 |
|  |  | NEVI (T2) | 0.96 (0.87, 1.06) |  | 1.17 (1, 1.38) |  | 0.94 (0.82, 1.07) |  | 0.94 (0.8, 1.12) |  |
|  |  | NEVI (T3) | 0.92 (0.82, 1.02) |  | 1.31 (1.12, 1.54) |  | 0.82 (0.71, 0.95) |  | 0.91 (0.75, 1.11) |  |

^1^ Modified Poisson regression models adjusted for age, sex, body mass index (BMI), smoking status, asthma, diabetes and hypertension

^2^ Abbreviations: PM_2.5_ – fine particulate matter; BC – black carbon; NO_2_ – nitrogen dioxide; O_3_ – ozone; ARDS – acute respiratory distress syndrome; NEVI – Neighborhood Environmental Vulnerability Index; EMM – effect measure modification by NEVI; Crude – Unadjusted for NEVI; Adj. NEVI – Adjusted for NEVI; T1/T2/T3 – NEVI Tertile strata (NEVI T1 identified as lowest tertile); Phase 1 – 3/2020-6/2020; Phases 2 & 3 – 7/2020-2/2021; 40% Subset – greater hospital catchment

**Supplement Table 7:** Risk ratio estimates for the relationship between chronic air pollution exposure (2009-2019) and COVID-19 pneumonia risk, stratified by phase and tertile of neighborhood environmental vulnerability index (NEVI)^1-2^

| Outcome | Poll. | NEVI  (Crude, Adjusted, Stratified) | Phase 1  Estimate (95%CI) | p-value for EMM^b^ | Phase 1  40% Subset Estimate (95%CI) | p-value  for EMM^b^ | Phases 2 & 3  Estimate (95%CI) | p-value  for EMM^b^ | Phases 2 & 3  40% Subset  Estimate (95%CI) | p-value for EMM^b^ |
| --- | --- | --- | --- | --- | --- | --- | --- | --- | --- | --- |
| Pneumonia | BC | Crude | 0.82 (0.76, 0.88) |  | 1.57 (1.43, 1.73) |  | 0.89 (0.87, 0.91) |  | 0.82 (0.79, 0.85) |  |
|  |  | Adj. NEVI | 0.9 (0.84, 0.96) |  | 1.45 (1.31, 1.6) |  | 0.87 (0.85, 0.89) |  | 0.82 (0.79, 0.85) |  |
|  |  | NEVI (T1) | 0.86 (0.79, 0.94) | 0.045 | 1.05 (0.93, 1.18) | <0.001 | 0.87 (0.83, 0.91) | 0.58 | 0.83 (0.77, 0.88) | 0.547 |
|  |  | NEVI (T2) | 0.81 (0.71, 0.93) |  | 1.47 (1.13, 1.91) |  | 0.89 (0.86, 0.93) |  | 0.81 (0.75, 0.86) |  |
|  |  | NEVI (T3) | 0.86 (0.76, 0.98) |  | 2.62 (2.19, 3.15) |  | 0.88 (0.85, 0.91) |  | 0.85 (0.81, 0.89) |  |
| Pneumonia | NO₂ | Crude | 1.21 (1.12, 1.3) |  | 1.76 (1.64, 1.88) |  | 0.79 (0.76, 0.82) |  | 0.77 (0.74, 0.81) |  |
|  |  | Adj. NEVI | 1.16 (1.1, 1.24) |  | 1.64 (1.51, 1.78) |  | 0.79 (0.76, 0.82) |  | 0.78 (0.75, 0.82) |  |
|  |  | NEVI (T1) | 0.94 (0.86, 1.02) | <0.001 | 1.13 (1.02, 1.25) | <0.001 | 0.87 (0.83, 0.92) | 0.001 | 0.83 (0.78, 0.89) | 0.183 |
|  |  | NEVI (T2) | 1.28 (1.13, 1.45) |  | 2.11 (1.73, 2.56) |  | 0.74 (0.69, 0.8) |  | 0.73 (0.66, 0.8) |  |
|  |  | NEVI (T3) | 1.91 (1.52, 2.4) |  | 6.36 (4.71, 8.6) |  | 0.74 (0.69, 0.78) |  | 0.76 (0.71, 0.83) |  |
| Pneumonia | O₃ | Crude | 0.98 (0.95, 1.01) |  | 0.81 (0.79, 0.83) |  | 1.07 (1.06, 1.08) |  | 1.09 (1.08, 1.11) |  |
|  |  | Adj. NEVI | 0.98 (0.96, 1) |  | 0.83 (0.81, 0.86) |  | 1.07 (1.06, 1.09) |  | 1.09 (1.07, 1.11) |  |
|  |  | NEVI (T1) | 1.04 (1.01, 1.07) | <0.001 | 0.96 (0.93, 1) | <0.001 | 1.05 (1.03, 1.08) | 0.069 | 1.07 (1.04, 1.1) | 0.177 |
|  |  | NEVI (T2) | 0.96 (0.91, 1) |  | 0.77 (0.72, 0.83) |  | 1.08 (1.06, 1.1) |  | 1.12 (1.09, 1.16) |  |
|  |  | NEVI (T3) | 0.92 (0.84, 1) |  | 0.49 (0.44, 0.54) |  | 1.08 (1.06, 1.1) |  | 1.09 (1.06, 1.11) |  |
| Pneumonia | PM_2.5_ | Crude | 0.94 (0.86, 1.02) |  | 1.47 (1.38, 1.56) |  | 0.85 (0.82, 0.88) |  | 0.82 (0.78, 0.85) |  |
|  |  | Adj. NEVI | 0.96 (0.91, 1.03) |  | 1.37 (1.28, 1.48) |  | 0.85 (0.82, 0.88) |  | 0.83 (0.79, 0.86) |  |
|  |  | NEVI (T1) | 0.89 (0.82, 0.97) | 0.001 | 1.05 (0.96, 1.15) | <0.001 | 0.88 (0.84, 0.92) | 0.148 | 0.87 (0.82, 0.92) | 0.096 |
|  |  | NEVI (T2) | 0.96 (0.82, 1.11) |  | 1.83 (1.48, 2.27) |  | 0.84 (0.79, 0.88) |  | 0.77 (0.7, 0.83) |  |
|  |  | NEVI (T3) | 0.97 (0.8, 1.17) |  | 4.61 (3.64, 5.84) |  | 0.83 (0.78, 0.87) |  | 0.81 (0.75, 0.87) |  |

^1^ Modified Poisson regression models adjusted for age, sex, body mass index (BMI), smoking status, asthma, diabetes and hypertension

^2^ Abbreviations: PM_2.5_ – fine particulate matter; BC – black carbon; NO_2_ – nitrogen dioxide; O_3_ – ozone; NEVI – Neighborhood Environmental Vulnerability Index; EMM – effect measure modification by NEVI; Crude – Unadjusted for NEVI; Adj. NEVI – Adjusted for NEVI; T1/T2/T3 – NEVI Tertile strata (NEVI T1 identified as lowest tertile); Phase 1 – 3/2020-6/2020; Phases 2 & 3 – 7/2020-2/2021; 40% Subset – greater hospital catchment

**Supplement Table 8:** Risk ratio estimates for the relationship between chronic air pollution exposure (2009-2019) and ventilation use risk, stratified by phase and tertile of neighborhood environmental vulnerability index (NEVI)^1-2^

| Outcome | Poll. | NEVI  (Crude, Adjusted, Stratified) | Phase 1  Estimate (95%CI) | p-value for EMM^b^ | Phase 1  40% Subset Estimate (95%CI) | p-value  for EMM^b^ | Phases 2 & 3  Estimate (95%CI) | p-value  for EMM^b^ | Phases 2 & 3  40% Subset  Estimate (95%CI) | p-value for EMM^b^ |
| --- | --- | --- | --- | --- | --- | --- | --- | --- | --- | --- |
| Ventilation | BC | Crude | 0.95 (0.91, 0.99) |  | 0.87 (0.82, 0.94) |  | 0.89 (0.84, 0.95) |  | 0.72 (0.66, 0.79) |  |
|  |  | Adj. NEVI | 0.96 (0.92, 1.01) |  | 0.92 (0.86, 0.99) |  | 0.84 (0.78, 0.9) |  | 0.7 (0.63, 0.78) |  |
|  |  | NEVI (T1) | 0.96 (0.89, 1.04) | 0.381 | 0.88 (0.78, 0.98) | 0.099 | 0.85 (0.75, 0.96) | 0.28 | 0.7 (0.57, 0.85) | 0.734 |
|  |  | NEVI (T2) | 0.97 (0.9, 1.05) |  | 0.85 (0.73, 1) |  | 0.88 (0.78, 0.99) |  | 0.66 (0.53, 0.81) |  |
|  |  | NEVI (T3) | 0.96 (0.9, 1.03) |  | 1.03 (0.93, 1.15) |  | 0.88 (0.8, 0.97) |  | 0.79 (0.69, 0.9) |  |
| Ventilation | NO₂ | Crude | 0.89 (0.84, 0.95) |  | 0.89 (0.83, 0.96) |  | 0.7 (0.64, 0.77) |  | 0.59 (0.52, 0.68) |  |
|  |  | Adj. NEVI | 0.93 (0.87, 0.99) |  | 0.95 (0.88, 1.02) |  | 0.67 (0.59, 0.76) |  | 0.6 (0.51, 0.71) |  |
|  |  | NEVI (T1) | 0.95 (0.87, 1.05) | 0.37 | 0.9 (0.8, 1) | 0.08 | 0.76 (0.64, 0.91) | 0.048 | 0.64 (0.51, 0.82) | 0.909 |
|  |  | NEVI (T2) | 0.9 (0.79, 1.02) |  | 0.89 (0.74, 1.07) |  | 0.65 (0.51, 0.82) |  | 0.54 (0.39, 0.74) |  |
|  |  | NEVI (T3) | 0.93 (0.83, 1.04) |  | 1.1 (0.96, 1.27) |  | 0.66 (0.56, 0.79) |  | 0.64 (0.51, 0.8) |  |
| Ventilation | O₃ | Crude | 1.04 (1.02, 1.06) |  | 1.07 (1.04, 1.11) |  | 1.1 (1.07, 1.13) |  | 1.18 (1.13, 1.23) |  |
|  |  | Adj. NEVI | 1.03 (1.01, 1.05) |  | 1.05 (1.02, 1.08) |  | 1.11 (1.07, 1.15) |  | 1.18 (1.12, 1.24) |  |
|  |  | NEVI (T1) | 1.03 (0.99, 1.06) | 0.889 | 1.07 (1.01, 1.12) | 0.296 | 1.11 (1.05, 1.19) | 0.724 | 1.19 (1.08, 1.3) | 0.861 |
|  |  | NEVI (T2) | 1.04 (1, 1.08) |  | 1.08 (1.01, 1.16) |  | 1.12 (1.05, 1.19) |  | 1.24 (1.11, 1.38) |  |
|  |  | NEVI (T3) | 1.02 (0.98, 1.06) |  | 1.01 (0.95, 1.06) |  | 1.07 (1.02, 1.13) |  | 1.12 (1.05, 1.19) |  |
| Ventilation | PM_2.5_ | Crude | 0.92 (0.87, 0.97) |  | 0.88 (0.81, 0.94) |  | 0.8 (0.74, 0.86) |  | 0.65 (0.58, 0.74) |  |
|  |  | Adj. NEVI | 0.94 (0.89, 1) |  | 0.93 (0.86, 1) |  | 0.77 (0.69, 0.85) |  | 0.66 (0.57, 0.77) |  |
|  |  | NEVI (T1) | 0.95 (0.88, 1.03) | 0.305 | 0.9 (0.81, 1) | 0.113 | 0.82 (0.71, 0.95) | 0.109 | 0.71 (0.58, 0.88) | 0.422 |
|  |  | NEVI (T2) | 0.95 (0.87, 1.05) |  | 0.86 (0.73, 1.02) |  | 0.79 (0.67, 0.93) |  | 0.57 (0.42, 0.77) |  |
|  |  | NEVI (T3) | 0.94 (0.84, 1.04) |  | 1.09 (0.93, 1.26) |  | 0.79 (0.68, 0.92) |  | 0.72 (0.59, 0.89) |  |

^1^ Modified Poisson regression models adjusted for age, sex, body mass index (BMI), smoking status, asthma, diabetes and hypertension

^2^ Abbreviations: PM_2.5_ – fine particulate matter; BC – black carbon; NO_2_ – nitrogen dioxide; O_3_ – ozone; NEVI – Neighborhood Environmental Vulnerability Index; EMM – effect measure modification by NEVI; Crude – Unadjusted for NEVI; Adj. NEVI – Adjusted for NEVI; T1/T2/T3 – NEVI Tertile strata (NEVI T1 identified as lowest tertile); Phase 1 – 3/2020-6/2020; Phases 2 & 3 – 7/2020-2/2021; 40% Subset – greater hospital catchment

**Supplement Table 9:** Risk ratio estimates for the relationship between chronic air pollution exposure (2009-2019) and dialysis use risk, stratified by phase and tertile of neighborhood environmental vulnerability index (NEVI)^1-2^

| Outcome | Poll. | NEVI  (Crude, Adjusted, Stratified) | Phase 1  Estimate (95%CI) | p-value for EMM^b^ | Phase 1  40% Subset Estimate (95%CI) | p-value  for EMM^b^ | Phases 2 & 3  Estimate (95%CI) | p-value  for EMM^b^ | Phases 2 & 3  40% Subset  Estimate (95%CI) | p-value for EMM^b^ |
| --- | --- | --- | --- | --- | --- | --- | --- | --- | --- | --- |
| Dialysis | BC | Crude | 0.76 (0.64, 0.91) |  | 1.21 (0.91, 1.61) |  | 0.78 (0.61, 1.00) |  | 1.16 (0.85, 1.59) |  |
|  |  | Adj. NEVI | 0.84 (0.69, 1.01) |  | 1.29 (0.99, 1.67) |  | 0.79 (0.59, 1.04) |  | 1.22 (0.84, 1.76) |  |
|  |  | NEVI (T1) | 0.99 (0.79, 1.24) | 0.087 | 1.00 (0.73, 1.37) | <0.001 | 0.85 (0.55, 1.32) | 0.632 | 0.95 (0.60, 1.51) | 0.008 |
|  |  | NEVI (T2) | 0.69 (0.47, 1.01) |  | 0.49 (0.10, 2.38) |  | 0.56 (0.34, 0.93) |  | 0.73 (0.27, 1.98) |  |
|  |  | NEVI (T3) | 0.69 (0.52, 0.92) |  | 2.64 (1.78, 3.90) |  | 0.84 (0.52, 1.35) |  | 1.94 (0.82, 4.60) |  |
| Dialysis | NO₂ | Crude | 1.14 (0.95, 1.36) |  | 1.58 (1.36, 1.83) |  | 0.93 (0.70, 1.24) |  | 1.21 (0.97, 1.52) |  |
|  |  | Adj. NEVI | 1.22 (1.05, 1.41) |  | 1.69 (1.41, 2.01) |  | 1.00 (0.76, 1.30) |  | 1.35 (1.00, 1.81) |  |
|  |  | NEVI (T1) | 1.09 (0.88, 1.36) | 0.064 | 1.15 (0.90, 1.47) | <0.001 | 0.83 (0.51, 1.35) | 0.093 | 0.93 (0.62, 1.40) | 0.004 |
|  |  | NEVI (T2) | 1.16 (0.85, 1.57) |  | 1.76 (1.12, 2.76) |  | 0.80 (0.45, 1.41) |  | 0.99 (0.52, 1.90) |  |
|  |  | NEVI (T3) | 1.56 (0.96, 2.54) |  | 7.02 (3.76, 13.1) |  | 1.66 (0.73, 3.76) |  | 4.15 (1.50, 11.5) |  |
| Dialysis | O₃ | Crude | 1.00 (0.93, 1.07) |  | 0.85 (0.80, 0.91) |  | 1.03 (0.93, 1.15) |  | 0.94 (0.85, 1.03) |  |
|  |  | Adj. NEVI | 0.97 (0.91, 1.03) |  | 0.83 (0.78, 0.89) |  | 1.01 (0.91, 1.13) |  | 0.91 (0.80, 1.03) |  |
|  |  | NEVI (T1) | 0.98 (0.90, 1.07) | 0.200 | 0.96 (0.87, 1.06) | <0.001 | 1.08 (0.91, 1.29) | 0.048 | 1.05 (0.88, 1.25) | 0.001 |
|  |  | NEVI (T2) | 1.01 (0.88, 1.14) |  | 0.88 (0.72, 1.09) |  | 1.07 (0.86, 1.33) |  | 0.98 (0.76, 1.27) |  |
|  |  | NEVI (T3) | 0.97 (0.80, 1.19) |  | 0.49 (0.40, 0.59) |  | 0.90 (0.66, 1.22) |  | 0.66 (0.45, 0.96) |  |
| Dialysis | PM_2.5_ | Crude | 0.88 (0.71, 1.10) |  | 1.29 (1.08, 1.54) |  | 0.81 (0.61, 1.08) |  | 1.07 (0.85, 1.34) |  |
|  |  | Adj. NEVI | 0.97 (0.82, 1.16) |  | 1.34 (1.11, 1.63) |  | 0.86 (0.66, 1.12) |  | 1.15 (0.87, 1.52) |  |
|  |  | NEVI (T1) | 1.02 (0.83, 1.25) | 0.360 | 1.05 (0.82, 1.33) | <0.001 | 0.86 (0.58, 1.27) | 0.519 | 0.91 (0.64, 1.31) | 0.005 |
|  |  | NEVI (T2) | 0.82 (0.53, 1.26) |  | 0.91 (0.30, 2.81) |  | 0.63 (0.35, 1.14) |  | 0.86 (0.43, 1.69) |  |
|  |  | NEVI (T3) | 0.80 (0.51, 1.24) |  | 5.80 (3.51, 9.58) |  | 1.00 (0.49, 2.07) |  | 3.08 (0.95, 9.97) |  |

^1^ Modified Poisson regression models adjusted for age, sex, body mass index (BMI), smoking status, asthma, diabetes and hypertension

^2^ Abbreviations: PM_2.5_ – fine particulate matter; BC – black carbon; NO_2_ – nitrogen dioxide; O_3_ – ozone; NEVI – Neighborhood Environmental Vulnerability Index; EMM – effect measure modification by NEVI; Crude – Unadjusted for NEVI; Adj. NEVI – Adjusted for NEVI; T1/T2/T3 – NEVI Tertile strata (NEVI T1 identified as lowest tertile); Phase 1 – 3/2020-6/2020; Phases 2 & 3 – 7/2020-2/2021; 40% Subset – greater hospital catchment

**Supplement Table 10:** Two-pollutant models: Risk ratio estimates for chronic air pollution exposure and adverse COVID-19 morbidity analyses, adjusting for O₃^1-2^

|  |  | **Phase 1** | | **Phases 2&3** | |
| --- | --- | --- | --- | --- | --- |
| Outcome | Poll. 1 | Poll. 1 Estimate (95%CI) | O_3_ Estimate (95%CI) | Pollutant 1 Estimate (95%CI) | O_3_ Estimate (95%CI) |
| ARDS | BC | 0.85 (0.78, 0.92) | 0.94 (0.9, 0.97) | 0.87 (0.77, 0.99) | 0.98 (0.92, 1.04) |
|  | NO₂ | 1.32 (1.14, 1.52) | 1.09 (1.04, 1.14) | 0.76 (0.63, 0.92) | 0.95 (0.89, 1.02) |
|  | PM_2.5_ | 0.9 (0.81, 1) | 0.96 (0.92, 1) | 0.86 (0.73, 1.01) | 0.98 (0.92, 1.04) |
| Dialysis | BC | 0.44 (0.32, 0.6) | 0.7 (0.62, 0.8) | 0.43 (0.25, 0.75) | 0.73 (0.58, 0.93) |
|  | NO₂ | 2.54 (1.56, 4.14) | 1.31 (1.1, 1.55) | 1.28 (0.59, 2.74) | 1.09 (0.83, 1.45) |
|  | PM_2.5_ | 0.63 (0.43, 0.92) | 0.81 (0.69, 0.95) | 0.55 (0.3, 1.03) | 0.82 (0.62, 1.08) |
| Length of Stay | BC | 0.98 (0.94, 1.02) | 0.99 (0.97, 1) | 0.97 (0.93, 1.01) | 0.98 (0.96, 1) |
|  | NO₂ | 1.02 (0.95, 1.09) | 1 (0.98, 1.02) | 1.22 (1.12, 1.32) | 1.05 (1.02, 1.08) |
|  | PM_2.5_ | 1.03 (0.97, 1.08) | 1 (0.98, 1.03) | 1.02 (0.96, 1.08) | 1 (0.97, 1.02) |
| Pneumonia | BC | 0.55 (0.49, 0.63) | 0.78 (0.74, 0.82) | 0.97 (0.92, 1.01) | 1.06 (1.04, 1.08) |
|  | NO₂ | 2.26 (1.82, 2.81) | 1.27 (1.18, 1.37) | 0.8 (0.74, 0.87) | 1.01 (0.98, 1.03) |
|  | PM_2.5_ | 0.63 (0.54, 0.74) | 0.82 (0.77, 0.88) | 0.96 (0.9, 1.03) | 1.06 (1.04, 1.08) |
| Ventilation | BC | 1.04 (0.95, 1.13) | 1.04 (1.01, 1.08) | 1.05 (0.91, 1.21) | 1.13 (1.06, 1.21) |
|  | NO₂ | 1.02 (0.89, 1.17) | 1.03 (0.99, 1.08) | 0.6 (0.48, 0.75) | 0.96 (0.9, 1.03) |
|  | PM_2.5_ | 1.02 (0.92, 1.13) | 1.04 (0.99, 1.08) | 0.9 (0.74, 1.09) | 1.07 (1, 1.15) |
|  |  | **Phase 1, 40% Subset** | | **Phases 2&3, 40% Subset** | |
| Outcome | Poll. | Poll. 1 Estimate (95%CI) | O_3_ Estimate (95%CI) | Pollutant 1 Estimate (95%CI) | O_3_ Estimate (95%CI) |
| ARDS | BC | 0.94 (0.82, 1.06) | 0.94 (0.89, 0.99) | 0.76 (0.63, 0.91) | 0.93 (0.86, 1) |
|  | NO₂ | 1.4 (1.16, 1.7) | 1.08 (1.01, 1.16) | 0.81 (0.63, 1.03) | 0.96 (0.87, 1.05) |
|  | PM_2.5_ | 0.97 (0.83, 1.12) | 0.95 (0.89, 1.01) | 0.84 (0.68, 1.04) | 0.96 (0.88, 1.05) |
| Dialysis | BC | 0.49 (0.27, 0.88) | 0.65 (0.53, 0.79) | 0.9 (0.36, 2.26) | 0.87 (0.62, 1.22) |
|  | NO₂ | 1.93 (1.12, 3.32) | 1.05 (0.86, 1.29) | 1.54 (0.52, 4.55) | 1.05 (0.69, 1.62) |
|  | PM_2.5_ | 0.48 (0.26, 0.89) | 0.61 (0.47, 0.79) | 0.58 (0.22, 1.54) | 0.97 (0.95, 1) |
| Length of Stay | BC | 0.98 (0.92, 1.04) | 0.99 (0.97, 1.02) | 0.97 (0.91, 1.04) | 0.97 (0.95, 1) |
|  | NO₂ | 0.99 (0.91, 1.08) | 1 (0.97, 1.03) | 1.16 (1.04, 1.28) | 1.03 (1, 1.07) |
|  | PM_2.5_ | 1.04 (0.96, 1.11) | 1.01 (0.98, 1.05) | 0.98 (0.9, 1.06) | 0.98 (0.94, 1.01) |
| Pneumonia | BC | 0.8 (0.62, 1.03) | 0.77 (0.7, 0.84) | 0.93 (0.87, 0.99) | 1.06 (1.03, 1.09) |
|  | NO₂ | 1.35 (1, 1.82) | 0.93 (0.83, 1.04) | 0.9 (0.82, 0.99) | 1.06 (1.02, 1.09) |
|  | PM_2.5_ | 0.55 (0.41, 0.74) | 0.64 (0.56, 0.74) | 1.04 (0.95, 1.13) | 1.11 (1.07, 1.14) |
| Ventilation | BC | 1.02 (0.91, 1.15) | 1.06 (1.01, 1.11) | 0.88 (0.72, 1.07) | 1.13 (1.03, 1.23) |
|  | NO₂ | 1.33 (1.11, 1.6) | 1.15 (1.08, 1.23) | 0.7 (0.53, 0.92) | 1.06 (0.97, 1.17) |
|  | PM_2.5_ | 1.09 (0.95, 1.26) | 1.08 (1.02, 1.15) | 0.85 (0.65, 1.12) | 1.12 (1.01, 1.23) |

^1^ Modified Poisson models used for ARDS, dialysis, pneumonia, ventilation; Cox proportional hazards models used for length of stay. Models adjusted for age, sex, body mass index (BMI), smoking status, asthma, diabetes, hypertension, and NEVI

^2^ Abbreviations: PM_2.5_ – fine particulate matter; BC – black carbon; NO_2_ – nitrogen dioxide; O_3_ – ozone; ARDS – Acute Respiratory Distress Syndrome; Poll. – Pollutant; NEVI – Neighborhood Environmental Vulnerability Index; Phase 1 – 3/2020-6/2020; Phases 2 & 3 – 7/2020-2/2021; 40% Subset – greater hospital catchment

**Supplement Table 11:** Risk ratio estimates for the relationship between chronic air pollution exposure and COVID-19 pneumonia risk, within racial and ethnic subpopulations of interest from March to June 2020 (within greater hospital catchment)^1-3^

| **Pollutant** | **Subpopulation** | **Estimate 95% CI** | **p-value for interaction** | |
| --- | --- | --- | --- | --- |
| BC | Black | 1.95 (1.57, 2.43) | 0.0004 |  |
|  | White | 1.09 (0.93, 1.27) |  |  |
| NO_2_ | Black | 2.04 (1.69, 2.47) | 0.001 |  |
|  | White | 1.24 (1.09, 1.43) |  |  |
| PM_2.5_ | Black | 1.68 (1.43, 1.98) | 0.001 |  |
|  | White | 1.09 (0.96, 1.24) |  |  |
| O_3_ | Black | 0.76 (0.71, 0.81) | 0.0004 |  |
|  | White | 0.92 (0.88, 0.97) |  |  |
| BC | Hispanic | 1.72 (1.43, 2.08) | 0.07 |  |
|  | Non-Hispanic | 1.39 (1.22, 1.58) |  |  |
| NO_2_ | Hispanic | 2.20 (1.87, 2.59) | 0.004 |  |
|  | Non-Hispanic | 1.57 (1.42, 1.74) |  |  |
| PM_2.5_ | Hispanic | 1.67 (1.43, 1.94) | 0.04 |  |
|  | Non-Hispanic | 1.34 (1.22, 1.47) |  |  |
| O_3_ | Hispanic | 0.74 (0.70, 0.79) | 0.002 |  |
|  | Non-Hispanic | 0.85 (0.82, 0.88) |  |  |

^1^ Risk ratios estimated from modified Poisson regression models, adjusted for age, sex, body mass index (BMI), smoking status, asthma, diabetes, hypertension, and NEVI in the 40% hospital catchment subset for Phase 1 (3/2020-6/2020)

^2^ Formal test of interaction was conducted by including an interaction term between the subpopulation variable of interest and pollutant exposure; statistical significance of the interaction term was determined using an alpha level of 0.05

^3^ Abbreviations: PM_2.5_ – fine particulate matter; BC – black carbon; NO_2_ – nitrogen dioxide; O_3_ – ozone; NEVI – Neighborhood Environmental Vulnerability Index; Phase 1 – 3/2020-6/2020; 40% Subset – greater hospital catchment

**Supplement Table 12:** Relative excess risk due to interaction (RERI) analyses between chronic air pollution exposure and neighborhood environmental vulnerability on adverse COVID-19 morbidities from March to June 2020 (within greater hospital catchment)^1-3^

| **Outcome** | **Poll.** | **Ref Base Poll.**  **and Low NEVI** | **Poll. IQR**  **Increase Only** | **High NEVI**  **Only** | **Poll. IQR Increase**  **and High NEVI** | **RERI** |
| --- | --- | --- | --- | --- | --- | --- |
| ARDS | BC – 2 abs | 1 | 1.02 (0.93, 1.12) | 0.91 (0.80, 1.04) | 1.06 (0.93, 1.21) | 0.13 (-0.02, 0.27) |
|  | NO₂ – 5 ppb | 1 | 1.13 (1.05, 1.21) | 0.99 (0.89, 1.10) | 1.33 (1.10, 1.61) | 0.21 (-0.01, 0.44) |
|  | PM_2.5_ – 1 µg/m^3^ | 1 | 1.06 (0.98, 1.13) | 0.96 (0.86, 1.07) | 1.26 (1.06, 1.51) | 0.25 (0.04, 0.46) |
|  | O_3_ – 1 ppb | 1 | 0.97 (0.94, 1.00) | 0.95 (0.86, 1.06) | 0.87 (0.76, 1.00) | -0.05 (-0.11, 0.005) |
| Dialysis | BC – 2 abs | 1 | 0.93 (0.58, 1.50) | 0.43 (0.26, 0.73) | 1.14 (0.75,1.75) | 0.78 (0.31, 1.24) |
|  | NO₂ – 5 ppb | 1 | 1.41 (1.14, 1.75) | 0.71 (0.47, 1.06) | 4.96 (2.72,9.06) | 3.84 (0.94, 6.74) |
|  | PM_2.5_ – 1 µg/m^3^ | 1 | 1.14 (0.86, 1.50) | 0.58 (0.37, 0.89) | 3.33 (2.02,5.49) | 2.62 (1.08, 4.15) |
|  | O_3_ – 1 ppb | 1 | 0.89 (0.82, 0.97) | 0.54 (0.35, 0.84) | 0.26 (0.15,0.46) | -0.17 (-0.29, -0.05) |
| Length of Stay | BC – 2 abs | 1 | 1.01 (0.97, 1.05) | 1.06 (0.99, 1.14) | 1.02 (0.95, 1.09) | -0.05 (-0.13, 0.03) |
|  | NO₂ – 5 ppb | 1 | 1.00 (0.96, 1.05) | 1.04 (0.98, 1.10) | 1.01 (0.91, 1.11) | -0.03 (-0.12, 0.05) |
|  | PM_2.5_ – 1 µg/m^3^ | 1 | 1.01 (0.97, 1.04) | 1.04 (0.98, 1.10) | 1.03 (0.94,1.14) | -0.01 (-0.11, 0.08) |
|  | O_3_ – 1 ppb | 1 | 0.99 (0.98, 1.01) | 1.05 (0.99, 1.11) | 1.08 (1.01,1.15) | 0.04 (-0.001, 0.07) |
| Pneumonia | BC – 2 abs | 1 | 1.24 (1.09, 1.40) | 0.48 (0.38, 0.62) | 1.26 (1.03, 1.55) | 0.54 (0.35, 0.74) |
|  | NO₂ – 5 ppb | 1 | 1.42 (1.31, 1.55) | 0.69 (0.57, 0.84) | 4.41 (3.32, 5.85) | 3.29 (2.08, 4.50) |
|  | PM_2.5_ – 1 µg/m^3^ | 1 | 1.25 (1.15, 1.36) | 0.62 (0.51, 0.76) | 2.87 (2.26, 3.65) | 1.99 (1.36, 2.62) |
|  | O_3_ – 1 ppb | 1 | 0.88 (0.85, 0.91) | 0.52 (0.42, 0.64) | 0.25 (0.19, 0.34) | -0.15 (-0.19, -0.10) |
| Ventilation | BC – 2 abs | 1 | 0.87 (0.79, 0.95) | 0.94 (0.83, 1.05) | 0.97 (0.86, 1.10) | 0.17 (0.03, 0.30) |
|  | NO₂ – 5 ppb | 1 | 0.89 (0.81, 0.98) | 1.03 (0.93, 1.14) | 1.13 (0.94, 1.36) | 0.21 (0.03, 0.40) |
|  | PM_2.5_ – 1 µg/m^3^ | 1 | 0.89 (0.82, 0.97) | 0.98 (0.88, 1.09) | 1.06 (0.89, 1.26) | 0.19 (0.02, 0.37) |
|  | O_3_ – 1 ppb | 1 | 1.07 (1.02, 1.11) | 1.01 (0.91, 1.12) | 1.02 (0.90, 1.15) | -0.06 (-0.13, 0.01) |

^1^ Modified Poisson models used for ARDS, dialysis, pneumonia, ventilation; Cox proportional hazards models used for length of stay. Models adjusted for age, sex, body mass index (BMI), smoking status, asthma, diabetes, hypertension, NEVI, and interaction with NEVI

^2^ NEVI score operationalized dichotomously [High NEVI: upper tertile (3.49-4.99) & N=4560; Low NEVI: lower two tertiles (2.14-3.48) & N=2794]

^3^ Abbreviations: PM_2.5_ – fine particulate matter; BC – black carbon; NO_2_ – nitrogen dioxide; O_3_ – ozone; µg/m^3^ – micrograms per cubic meter; abs – absorbance units; ppb – parts per billion**;** Poll – Pollutant; NEVI – Neighborhood Environmental Vulnerability Index; RERI – relative excess risk due to interaction; IQR – interquartile range (as specified by the pollutant column); Phase 1 – 3/2020-6/2020; 40% Subset – greater hospital catchment

**Supplement Table 13:** Hazard ratios and corresponding 95% confidence intervals for length of hospitalization stay during Phases 2 & 3 (7/2020-2/2021), stratified by tertile of area-level COVID-19 testing^1, 2^

|  | **Testing Tertile 1** | | **Testing Tertile 2** | | **Testing Tertile 3** | |
| --- | --- | --- | --- | --- | --- | --- |
|  | **HR** | **95% CI** | **HR** | **95% CI** | **HR** | **95% CI** |
| **BC** | 1.04 | 0.96, 1.12 | 1.04 | 0.99, 1.09 | 1.04 | 1.00, 1.09 |
| **NO_2_** | 1.18 | 1.00, 1.39 | 1.18 | 1.10, 1.26 | 1.07 | 1.03, 1.11 |
| **PM_2.5_** | 1.10 | 0.97, 1.25 | 1.11 | 1.04, 1.18 | 1.05 | 1.01, 1.08 |
| **O_3_** | 0.98 | 0.94, 1.02 | 0.98 | 0.96, 1.00 | 0.98 | 0.96, 0.99 |

^1^ Testing tertiles were calculated from aggregated weekly testing data from the NYC Department of Health and Mental Hygiene

^2^ Abbreviations: PM_2.5_ – fine particulate matter; BC – black carbon; NO_2_ – nitrogen dioxide; O_3_ – ozone

**Supplement Figure 1:** Correlation heatmap identifying Spearman correlations between overall NEVI score, individual NEVI domain scores and chronic air pollution (2009-2019) metrics for New York City^1^


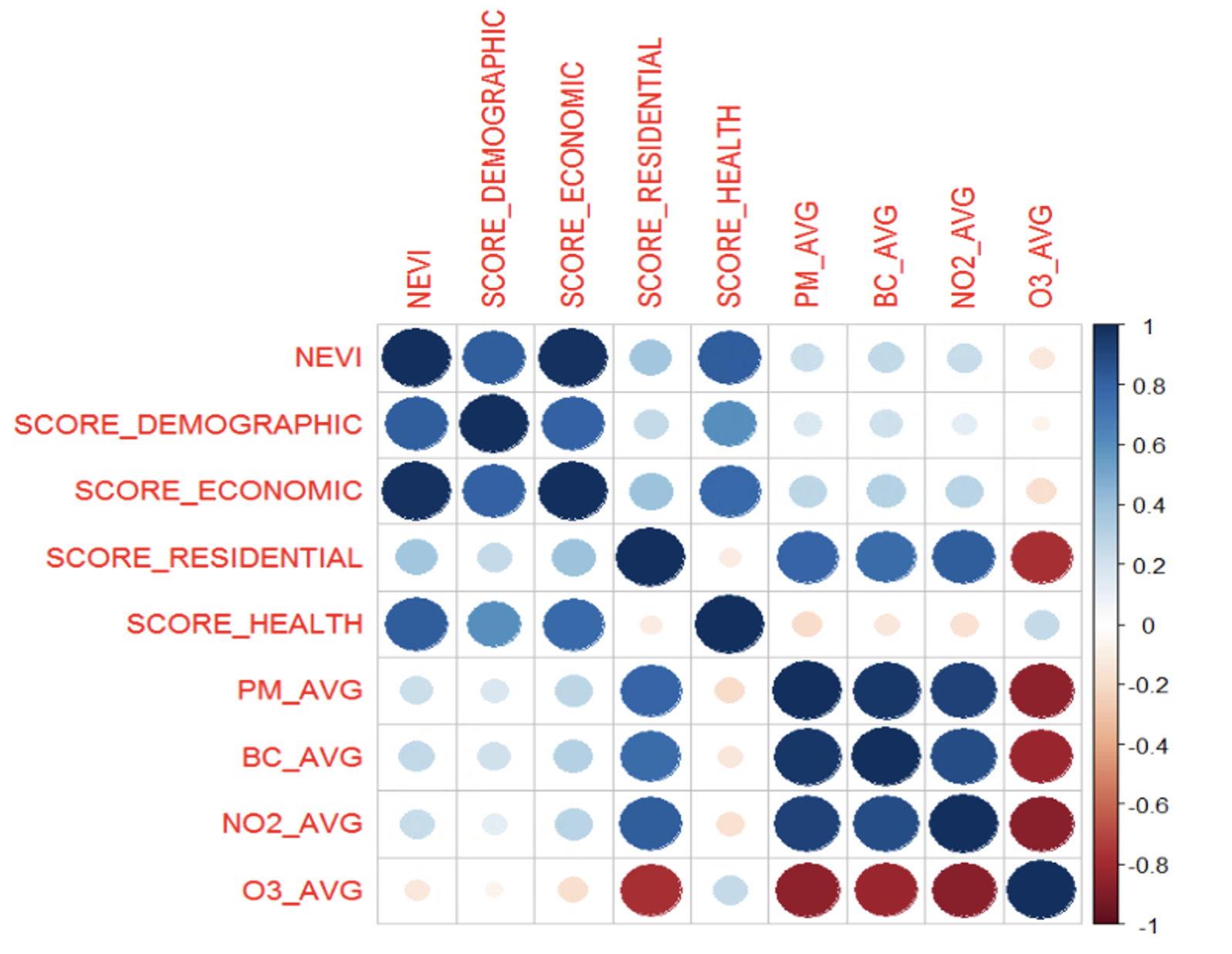


^1^ Abbreviations: NEVI – Neighborhood Environmental Vulnerability Index; SCORE_DEMOGRAPHIC – Demographic NEVI Score; SCORE_ECONOMIC – Economic NEVI Score; SCORE_RESIDENTIAL – Residential NEVI Score; SCORE_HEALTH – Health NEVI Score; PM_AVG – 2009-2019 average for fine particulate matter (PM_2.5_) expressed in µg/m^3^; BC_AVG – 2009-2019 average for black carbon (BC) expressed in absorbance units; NO2_AVG – 2009-2019 average for nitrogen dioxide (NO_2_) expressed in parts per billion (ppb); O3_AVG – 2009-2019 average for ozone (O_3_) expressed in parts per billion (ppb)

**Supplement Figure 2:** Graphical depiction of results from simulations to determine the selection factors that could explain the study observed results related to ARDS risk^1^


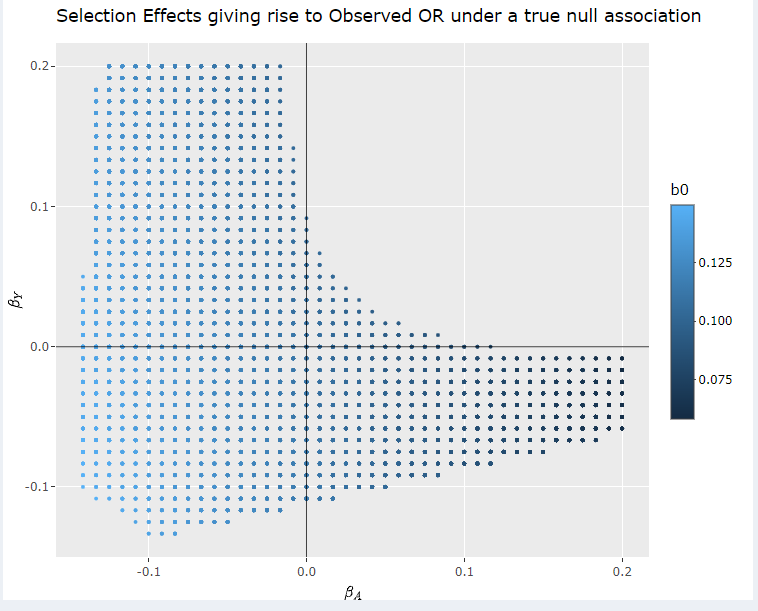


^1^ Figure was created using AscRtain, an R package and SHINY app. The location of dots indicates areas where selection factors could plausibly explain the observed study results. The x-axis shows selection differential based on exposure and y-axis shows selection values differential based on outcome. The lack of points within the upper-right quadrant of the plot suggests that selection bias is less likely of an explanation of results if the selection of the exposed population (i.e. those with higher air pollution) into hospital was greater than the baseline risk of hospitalization in the population.
